# In Search of Wasserman’s Catenane

**DOI:** 10.1021/jacs.3c01939

**Published:** 2023-04-25

**Authors:** Andrei S. Baluna, Albano Galan, David A. Leigh, Gareth D. Smith, Justin T. J. Spence, Daniel J. Tetlow, Iñigo J. Vitorica-Yrezabal, Min Zhang

**Affiliations:** † Department of Chemistry, University of Manchester, Oxford Road, Manchester M13 9PL, United Kingdom; ‡ School of Chemistry and Molecular Engineering, East China Normal University, 200062 Shanghai, China

## Abstract

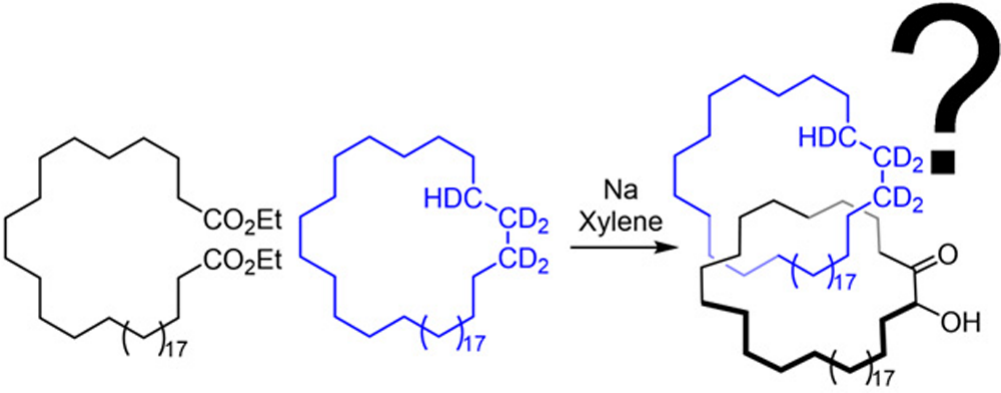

We repeat the earliest claimed [2]catenane synthesis, reported by Wasserman over 60 years ago, in order to ascertain whether or not a nontemplate, statistical synthesis by acyloin macrocyclization does indeed form mechanically interlocked rings. The lack of direct experimental evidence for Wasserman’s catenane has led to it being described as a “prophetic compound”, a technical term used in patents for claimed molecules that have not yet been synthesized. Contemporary synthetic methods were used to reconstruct Wasserman’s deuterium-labeled macrocycle and other building blocks on the 10–100 g reaction scale necessary to generate, in principle, ∼1 mg of catenane. Modern spectrometric and spectroscopic tools and chemical techniques (including tandem mass spectrometry, deuterium nuclear magnetic resonance (NMR) spectroscopy, and fluorescent tag labeling) were brought to bear in an effort to detect, isolate, and prove the structure of a putative [2]catenane consisting of a 34-membered cyclic hydrocarbon mechanically linked with a 34-membered cyclic α-hydroxyketone.

## Introduction

**Scheme 1:**
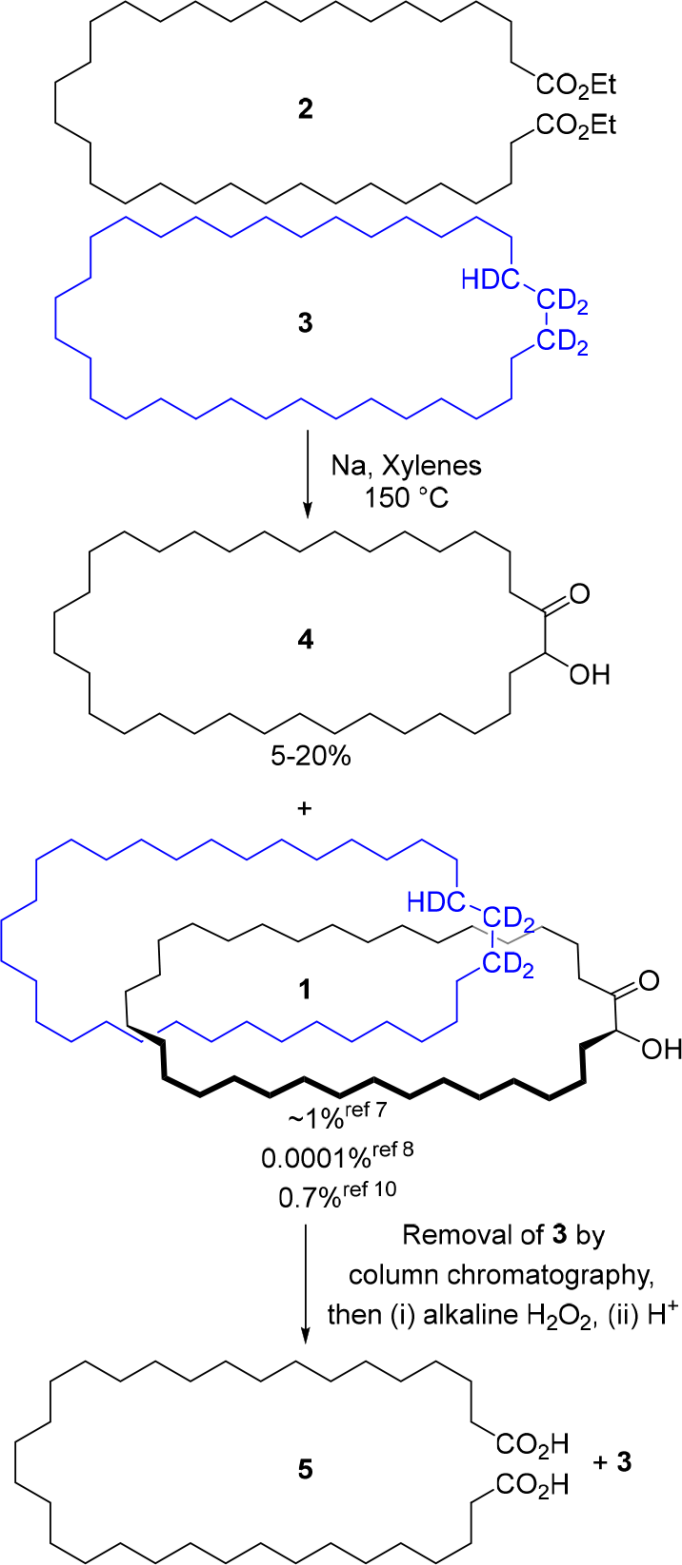
Wasserman’s Claimed^7^ 1960 Synthesis of [2]Catenane **1** by Statistical Threading and Macrocyclization of Alkyl Diester **2** through Deuterium-Labeled Cyclohydrocarbon **3** via Acyloin Condensation The experimental evidence for the interlocked structure in 1960^7^ was that following removal of **3** from the product mixture from the acyloin condensation by chromatography, oxidation (to cleave the acyloin group) released detectable quantities of **3**. It was argued that the newly released **3** could only arise from it having been interlocked with **4** in a catenane (i.e., **1**).^7,8^

In recent decades, mechanically linked molecules^1−6^ have become structures of broad and general interest. The first synthesis of an interlocked molecule—a molecular Hopf link or [2]catenane^1−4^—was claimed by Edel Wasserman in a *Communication to the Editor*, published in the *Journal of the American Chemical Society* in 1960.^7^ Wasserman’s “statistical approach”,^8^ carried out at Bell Telephone Laboratories with Louis Barash,^9^ sought to achieve the synthesis of catenane **1** (Scheme 1) by macrocyclization of diester **2** (through sodium-mediated acyloin condensation) using deuterated cyclohydrocarbon **3** as a cosolvent with a mixture of xylenes.^7^ The claimed yield for **1** was very low (variously reported as 0.0001%,^8^ 0.7%,^10^ or ∼1%^7^). Furthermore, the evidence for the catenane structure was indirect:^7^ the reaction mixture from the acyloin condensation was passed through a silica chromatography column, eluting with pentane to remove **3**. Acyloin **4** and other reaction products were then eluted together from the column with diethyl ether and methanol. Analysis of this purportedly **3**-free semipurified product mixture by infrared (IR) spectroscopy indicated the presence of deuterium, which—if all of **3** had indeed been removed by chromatography—could reasonably be attributed to **1** having been formed in the acyloin condensation reaction.^7^ The presence of **1** in this mixture was also claimed^7^ to be “*demonstrated by its chromatographic behavior, infrared spectrum, m.p. and mixed m.p.*”, but no experimental data was disclosed (which was not uncommon for *Communications* at the time).

The seemingly **3**-free product mixture was then oxidized with alkaline hydrogen peroxide to cleave the acyloin functional group (Scheme 1).^7^ This afforded diacid **5**, but deuterated macrocycle **3** was also isolated from this oxidation reaction. If all of the excess **3** had been removed through the earlier chromatography step, **3** could only arise from it being released intact from a compound produced by the original acyloin macrocyclization reaction. In other words, the presence of **3**, apparently liberated by oxidative cleavage of the acyloin macrocycle, was evidence that catenane **1** was formed in the original macrocyclization acyloin condensation reaction (Scheme 1).^7^

The lack of provision of any experimental characterization data for **1**, the very low yield (and the substantial variations in what that yield actually was in different reports by Wasserman), and the sole, indirect evidence for the formation of at most ∼1 mg of catenane resting on the complete removal of a 10 g excess of deuterated starting material all meant that the claimed synthesis of catenane **1** has long been viewed with varying degrees of skepticism. These views were crystallized by Brückner^11^ following the award of the 2016 Nobel Prize in Chemistry, which in some respects^11,12^ conflated synthetic molecular machinery with catenane and rotaxane (and knot) chemistry. In a scholarly Perspective on the early work on mechanically interlocked molecules by Lüttringhaus and Schill,^13^ he detailed and assessed Wasserman’s claims of statistical catenane synthesis.^11^ Brückner concluded that the “*scarcity, inconsistency, and even incredibility of pertinent data*” for **1** indicates that it should be considered as a “prophetic compound”,^11^ a technical term^14^ in patents used for molecules claimed without them having already been synthesized.

Given the historical significance of the landmark first synthesis of a catenane and that the statistical synthesis of catenanes has rarely been explored since,^15,16^ we decided to repeat Wasserman’s synthesis using modern synthetic methods, instrumentation, and purification and characterization techniques to try and determine whether catenane **1** had really been made or not. Of course, one cannot prove a negative. So, if we were unable to detect catenane formation, it would not prove that the reaction did not occur, but it would at least allow us to report whether or not we were able to reproduce the findings of the seminal 1960 paper.^7^

## Results and Discussion

### Scalable Synthesis and Structural Characterization of Wasserman’s Catenane Building Blocks

**Scheme 2:**
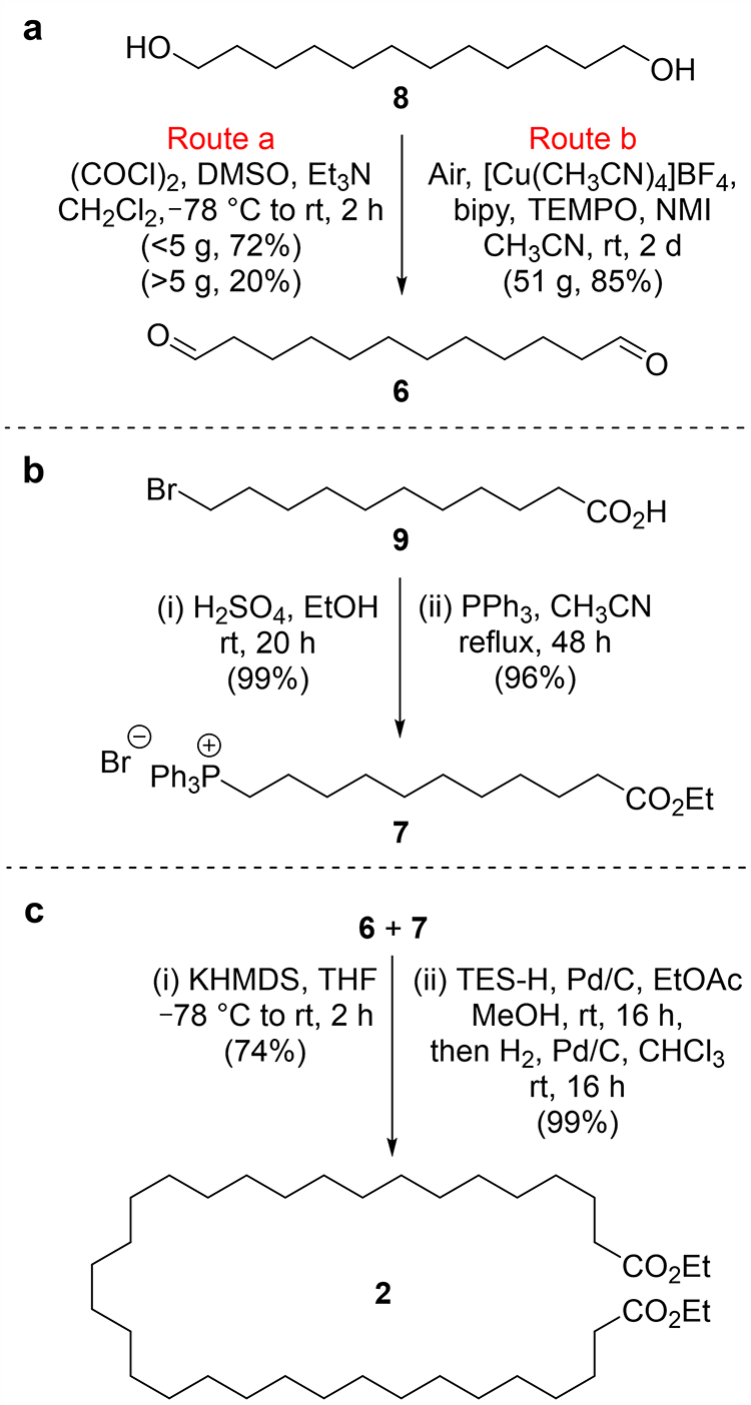
Synthesis of Diester **2** (a) Synthesis of dialdehyde **6**, (b) synthesis of phosphonium bromide **7**, and (c) Wittig olefination of **6** + **7** and hydrogenation of the resulting alkene to form **2**. bipy = 2,2′-bipyridine; TEMPO = (2,2,6,6-tetramethyl-piperidin-1-yl)oxidanyl; NMI = *N*-methylimidazole; KHMDS = potassium bis(trimethylsilyl)amide; TES-H = triethylsilane.

Even the highest (∼1%^7^) of the yields attributed by Wasserman to the synthesis of **1**, together with the amount of the isotopically labeled cyclic hydrocarbon cosolvent needed to achieve it, meant that at least 10 g of deuterated **3** would be required for every putative catenane-forming acyloin condensation reaction to allow the potential isolation of ∼1 mg of catenane **1**.^7,10^ In the 1960 report,^7^**2** was prepared through a series of Kolbe electrolysis reactions of a diester.^17,18^ To avoid large-scale electrolysis, we instead explored a route to **2** that utilized a double Wittig reaction between dialdehyde **6** and phosphonium bromide **7**, followed by hydrogenation of the resulting alkenes to afford 34-carbon chain diester **2** (Scheme 2).

Dialdehyde **6** was obtained by oxidation of the commercially available diol **8**. Swern oxidation^19^ gave **6** in good yields on modest scales (72% from 15 mmol of **8**; Scheme 2a, route a). However, the Swern reaction proved poorly scalable (e.g., 20% isolated yield of **6** from 51 mmol of **8**). Pleasingly, a copper-catalyzed oxidation procedure from Stahl^20^ proved to be both effective and reproducible in larger quantities (85% isolated yield of **6** from 252 mmol of **8**; Scheme 2a, route b), enabling **6** to be prepared on >50 g scale. Phosphonium bromide **7** was prepared in two steps from commercially available 11-bromoundecanoic acid **9** (Scheme 2b). Double Wittig olefination of **6** + **7** gave the dialkene (74%), which was reduced via a two-step procedure^21^ to give **2** in near-quantitative yield (Scheme 2c).

**Scheme 3:**
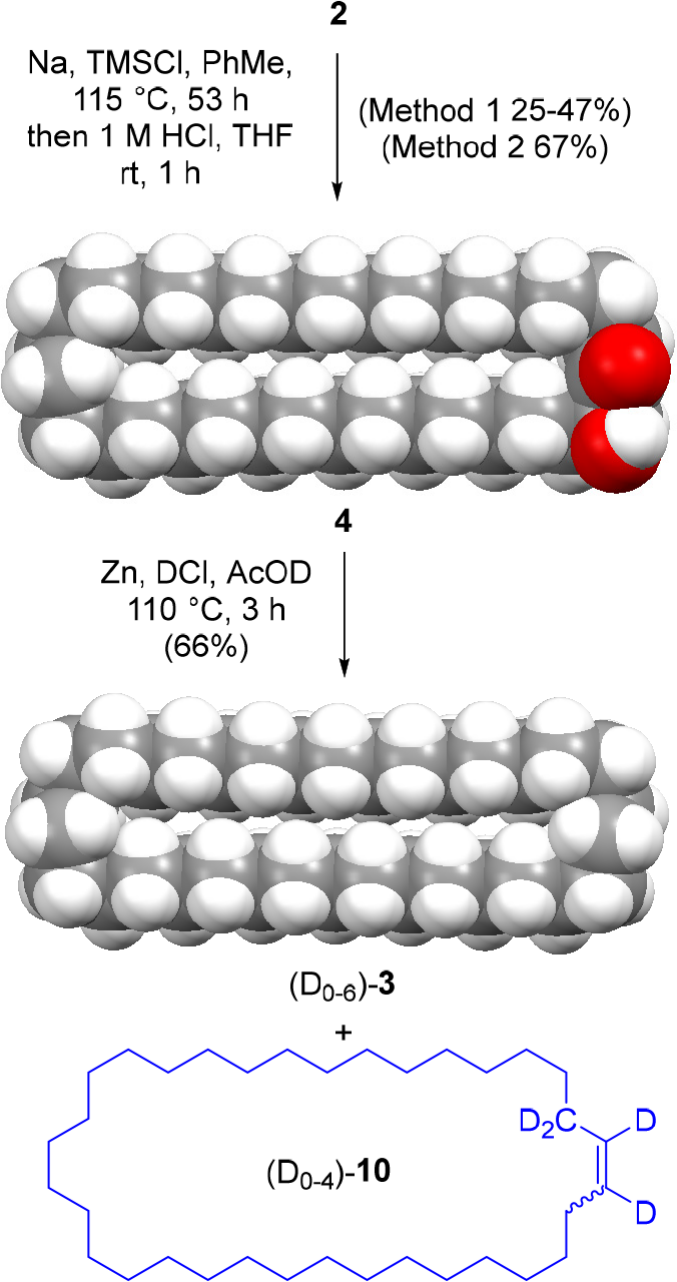
Synthesis of Cyclic Acyloin **4** and Deuterated Cycloalkane/-alkene (D_0-6_)-**3**/(D_0-4_)-**10** X-ray crystal structures of (D_0-6_)-**3** (the alkene group of **10** present in the crystal manifests itself as disorder distributed around the macrocycle) and **4**, depicted in van der Waals radius space-filling representations.

Diester **2** is both a component in the putative catenane-forming reaction (Scheme 1) and an intermediate in the synthesis of the other required organic building block, **3** (Scheme 3). Diester **2** was converted to macrocycle **4** via a modified acyloin condensation procedure (Scheme 3).^22^ Using temperature-controlled dropwise addition^23,24^ of the diester to the refluxing sodium dispersion, the yield of **4** on a 7–10 g scale was increased from 25% (without this method) to 67% (Supporting Information, Section 4). The structure of **4** was confirmed by nuclear magnetic resonance (NMR) spectroscopy, high resolution mass spectrometry (HRMS), and X-ray crystallography (Scheme 3). Clemmensen reduction of **4** with zinc in the presence of DCl and deuterated acetic acid (the procedure used by Wasserman) gave the deuterated cyclohydrocarbon in 66% yield, which crystallized from pentane upon standing.^25^

**Figure 1:**
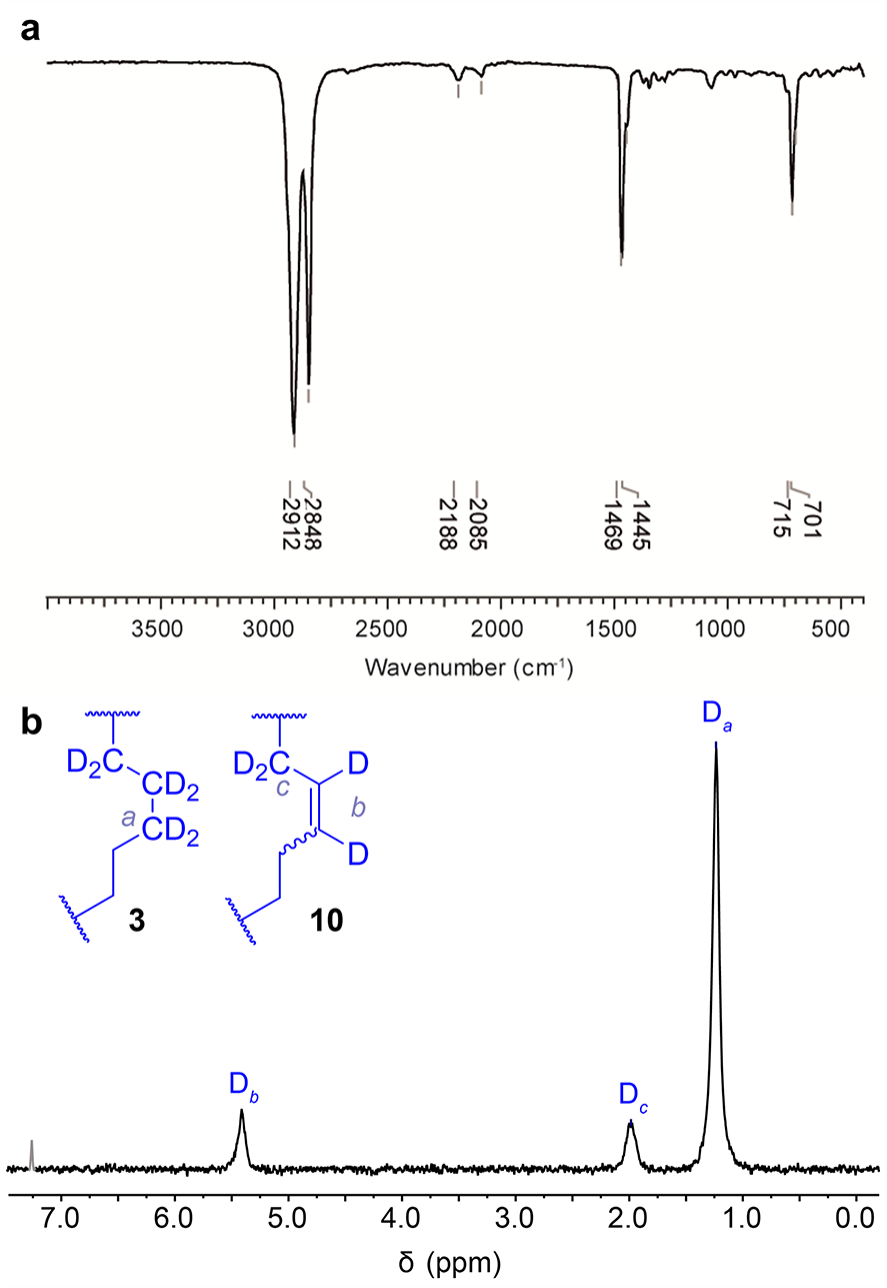
(a) IR spectrum of a crystal of (D_0–6_)-**3**/(D_0–4_)-**10** and (b) ^2^H NMR spectrum (77 MHz, CHCl_3_, 298 K) of (D_0–6_)-**3**/(D_0–4_)-**10**. Signal shaded gray corresponds to trace CDCl_3_.

IR spectroscopy of a crystal of (D_0–6_)-**3** showed low intensity bands in the region for C–D stretching^26^ (2188 and 2085 cm^–1^; Figure 1a). IR spectroscopy was used by Wasserman to detect the presence of catenane **1** in the **3**-free product mixture.^7^ The IR spectrum of deuterated **3** published in ref (8) has a band of moderate intensity for C–D absorption at 2174 cm^–1^ with a shoulder at 2130 cm^–1^ rather than the three bands at 2105, 2160, and 2200 cm^–1^ reported in ref (7). Due to these inconsistencies and the relatively low intensities of the C–D IR bands in our samples of neat deuterated-**3**, we were concerned that the presence of deuterium might not be assignable with confidence if the deuterated catenane made up, at most, 1% of a mixture with other organic compounds. We therefore explored other contemporary analysis methods to confirm and quantify deuterium incorporation that might be usefully extended to putative catenane detection.

^2^H NMR spectroscopy of **3** obtained from Scheme 3 (Figure 1b) confirmed the presence of deuterium (δ2_H_ 1.24 ppm). In addition, resonances in the C=CD alkene and C=CCD regions (δ2_H_ 5.41 and 1.98 ppm, Figure 1b) indicated that the cyclic hydrocarbon also contained some alkene (**10**). The presence of D in the alkene and the adjacent methylene group are consistent with this byproduct arising from elimination during the Clemmensen reduction of the acyloin.^27^ Atmospheric pressure chemical ionization (APCI) mass spectrometry^28^ showed the incorporation of up to 6 deuterium atoms in **3** and up to 4 deuterium atoms in **10**. The mass spectra show that the nondeuterated isotopomers, (D_0_)-**3** and (D_0_)-**10**, make up <3% of each sample, so the weak IR bands in Figure 1a are not a sign of ineffective deuterium incorporation during the Clemmensen reduction (Scheme 3). (D_0–6_)-**3** and (D_0–4_)-**10** proved inseparable by chromatography, and so, our investigation continued using the two species as a mixture (∼3:1 (D_0–6_)-**3**:(D_0–4_)-**10**). It seems likely that Wasserman’s Clemmensen reduction also produced **3** contaminated with **10** and that a mixture of **3**/**10** was used in the 1960 statistical catenane synthesis experiment.

The X-ray crystal structures of **3** and **4**, the two molecular components that are linked in putative catenane **1**, are shown with their atoms at van der Waals radius in Scheme 3. The cavities of both macrocycles are collapsed in the crystalline state in order to maximize packing interactions. The situation will obviously be very different in an acyloin condensation macrocyclization reaction, where the *p*-xylene solvent molecules will solvate the surfaces of both components and sodium will interact (and react) with the ester groups. Nevertheless, given the lack of designed template interactions for threading (an approach proposed by Sokolov a decade later^29^ and, independently, experimentally introduced by Sauvage a decade after that^2,5^), the flexibility of the alkyl chains, the entropic cost of macrocyclization, and the lack of driving force for the entwining of **2** and **3**, we considered that it might not be surprising if catenane formation during the acyloin condensation turned out to be a vanishingly rare statistical event.

### Attempted Reproduction of Wasserman’s Statistical Catenane Synthesis

**Figure 2:**
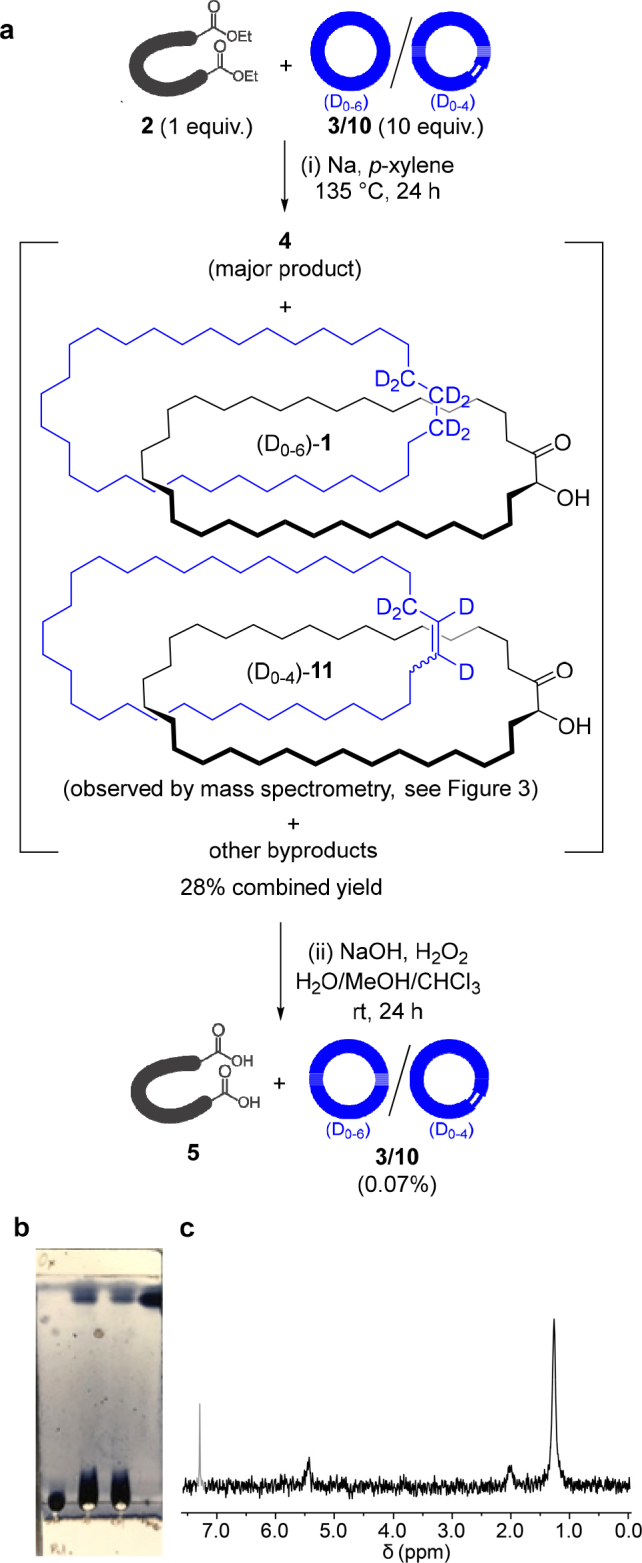
(a) (i) Synthesis of [2]catenane **1**/**11** by statistical threading. (ii) Oxidation of catenane-containing mixture with alkaline H_2_O_2_. (b) Photograph of TLC analysis (silica, petroleum ether; cerium ammonium molybdate stain) of the oxidative cleavage reaction on the polar fraction obtained from step (i). Left-hand lane: polar fraction from the catenane-forming reaction (i.e., products from step (i)); 2nd-from-left lane: co-spot of before and after oxidative cleavage; 2nd-from-right lane: oxidation reaction (i.e., step (ii)) after 24 h; right-hand lane: pristine **3**/**10**. (c) ^2^H NMR spectrum (77 MHz, CHCl_3_, 298 K) of fast-eluting product from step (ii), i.e., **3**/**10** (0.07% yield, based on starting material **2**).

With several 10s of grams of the deuterium-labeled macrocycle (D_0–6_)-**3**/(D_0–4_)-**10** and diester **2** in hand, we attempted to repeat Wasserman’s statistical catenane synthesis (Scheme 1). His original report^7^ does not include detailed experimental procedures, so we investigated several different sets of conditions, concentrations, starting material ratios, and various processes for the addition of reagents and stirring,^23^ before settling on the following (Figure 2a, step (i)): Diester **2** was added to a dispersion of sodium in liquid **3**/**10**:*p*-xylene (1:1 *v*/*v*, corresponding to a 12.4:1 molar ratio of **3**/**10**:**2**) at 90 °C (**3**/**10** melts at 61–63 °C) under mechanical stirring. The reaction was then heated at 135 °C for 24 h. The reaction mixture was allowed to cool to room temperature, quenched with methanol, extracted into CHCl_3_, and concentrated under reduced pressure. The resulting waxy solid was subjected to column chromatography on silica gel. The large excess of **3**/**10** was removed by elution with petroleum ether. This was followed by elution with a mixture of petroleum ether–ethyl acetate (1:1), which gave an acyloin-containing polar fraction that appeared to be free of **3**/**10** in 28% yield based on **2**. (Wasserman reports^7^ the yield of **4** from this reaction to vary between 5% and 20%.) ^1^H NMR spectroscopy confirmed the major species in our polar fraction to be **4**. ^2^H NMR spectroscopy indicated the presence of a small amount of deuterium (signals at δ2_H_ 5.41, 1.97, and 1.23 ppm; Figure S4).

**Figure 3:**
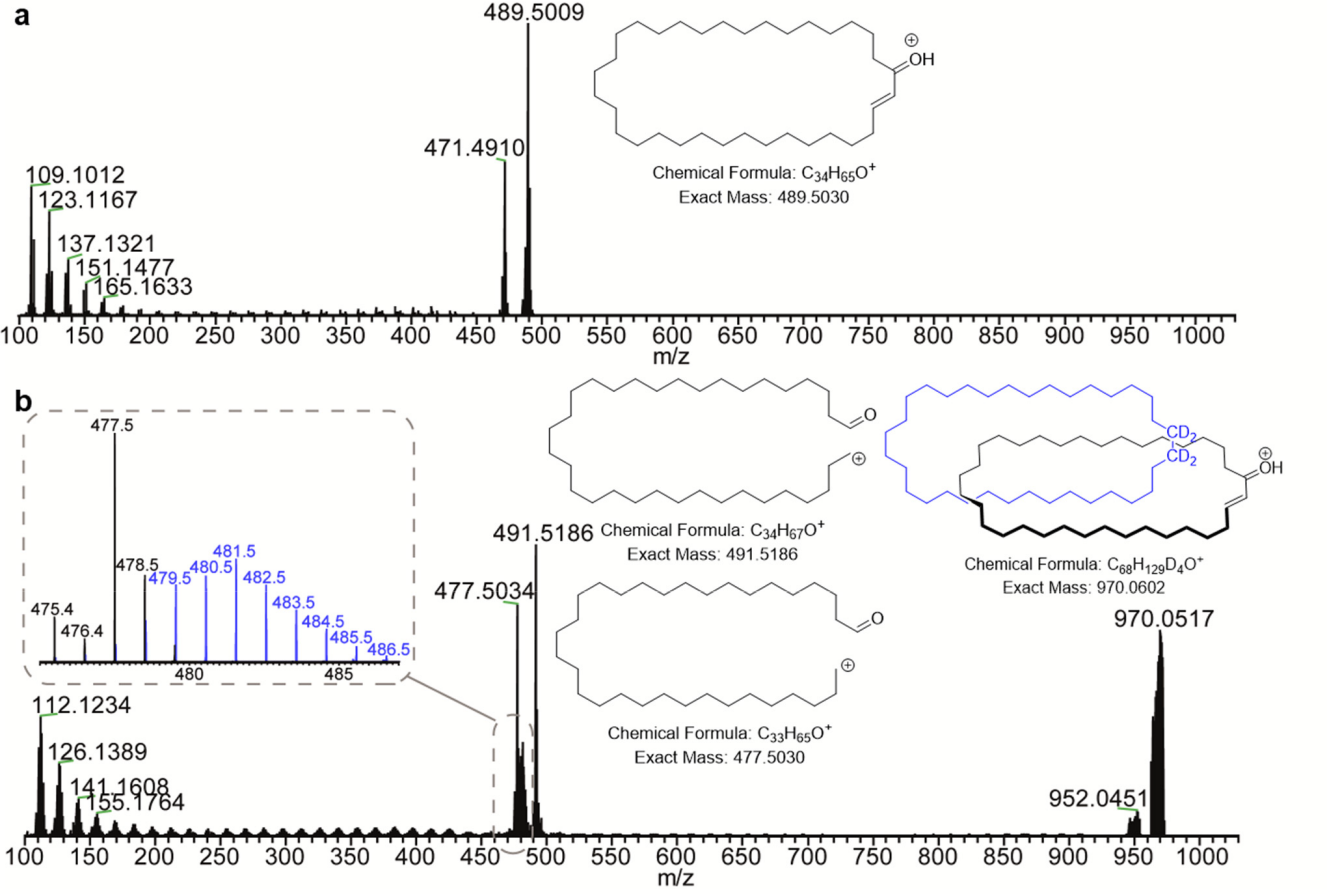
APCI(+)-MS/MS tandem mass spectrometry of key molecular ions present in the polar fraction eluted from Figure 2, step (i) after removal of excess **3**/**10** by column chromatography. (a) MS/MS of *m*/*z* 489, corresponding to the acyloin macrocycle [**4**-H_2_O]H^+^, using 20 eV collision energy. The fragment at *m*/*z* 471 corresponds to [**4**-2H_2_O]H^+^. (b) MS/MS of *m*/*z* 970, corresponding to catenane [**1**/**11**-H_2_O]H^+^, using 20 eV collision energy. Inset: Signals in blue arise from fragments of (D_0–6_)-**3** and (D_0–4_)-**10**; signals in black arise from fragments of **4**.

Analysis of the polar fraction by mass spectrometry confirmed **4** as being the major component (*m*/*z* = 489, [**4**-H_2_O]H^+^, Figure S17). Also apparent were ions corresponding to a macrocyclic acyloin dimer, **S7**(29) (*m*/*z* = 996, [**S7**-H_2_O]H^+^). However, also present was a very low intensity set of ions with *m*/*z* values clustered around 970 (the distribution of *m*/*z* values corresponding to the distribution of the deuterium incorporated in (D_0–6_)-**3**; Figure S18). To our delight, and some surprise, these corresponded to the molecular mass of catenane **1**/**11** after a loss of water (*m*/*z* = 964 to 972, [**1**/**11**-H_2_O]H^+^). Fragmentation of the *m*/*z* 970 ion by tandem MS/MS revealed the release of charged fragments corresponding to either the acyloin macrocycle **4** (*m*/*z* = 491 and 477) or the deuterated macrocycle **3**/**10** (*m*/*z* = 473 to 481) indicating the presence of both rings in the parent molecule (Figure 3b).

Several features of the tandem mass spectrum of the ion at *m*/*z* = 970 demonstrate unequivocally that it has the structure of a catenane:

(i) The mass of the ion is the sum of the molecular masses of **3** and **4** (minus H_2_O, which is readily eliminated from acyloin compounds under mass spectrometry conditions).
(ii) The isotope pattern of the *m*/*z* = 970 ion is the same as the deuterium distribution in **3**.
(iii) Fragmentation of the ion at *m*/*z* = 970 gives ions corresponding to one or another of the macrocycles. However, no fragments are observed of molecular mass in between that of the catenane and a single macrocycle. This is because, once one ring of the catenane is opened by bond cleavage, the linear fragment dethreads from the other, still closed, ring. This is characteristic behavior of catenanes in mass spectrometry (particularly those lacking strong attractive interactions between the components), originally described by Schill.^13^

### Quantification of the Amount of Catenane Formed in the Statistical Interlocking Reaction

We continued our investigation of the 1960 paper^7^ by attempting to reproduce the oxidative cleavage of the acyloin group in the polar fraction obtained by chromatography, free from **3** but containing acyloin macrocycle **4** and catenane **1**/**11** and likely other byproducts. Treatment of this fraction with alkaline H_2_O_2_ led to the formation of two new species, one of which had the same *R*_F_ value on thin layer chromatography (silica, petroleum ether eluent; Figure 2b) as **3**/**10**.^30^ This product was isolated and its identity confirmed by ^2^H NMR (Figure 2c) and mass spectrometry (Figure S6) to be **3**/**10**. Based on the recovery of **3**/**10** from this sample, the fraction of diester **2** converted into catenane by the acyloin condensation reaction shown in Figure 2a; step (i) is 0.07%.

To confirm that the presence of **3**/**10** was due to its release from a catenane and not incomplete chromatographic separation, a pristine sample of acyloin **4** (200 mg) was mixed with **3**/**10** (7 g). The mixed sample was subjected to the same chromatographic purification procedure as described previously (silica gel, eluting with petroleum ether, and then a mixture of petroleum ether and ethyl acetate). ^2^H NMR spectroscopy showed no deuterium present in the polar fraction from this control experiment. It was then submitted to alkaline oxidation conditions and, this time, analysis showed no **3**/**10** released by this reaction (Figure S7a,b).

### Derivatization and Isolation of a [2]Catenane Prepared by Statistical Synthesis

Our results corroborate Wasserman’s original observations,^7,8^ not only that the statistical synthesis of catenane **1** occurs, but also that the indirect “proof-of-structure” is valid and reproducible. Moreover, the modern day reproduction of the latter experiment shows that the amount of catenane formed in the statistical synthesis is at least 0.07%. However, our attempts to isolate the catenane from the mixture with the acyloin macrocycle were unsuccessful. Our efforts were hindered by the small amount of catenane present in a mixture of other chemically similar compounds and by the apolar nature of the catenane and its lack of functionality, for example chromophores, that could aid its detection as a very minor component of a complex mixture.

**Figure 4:**
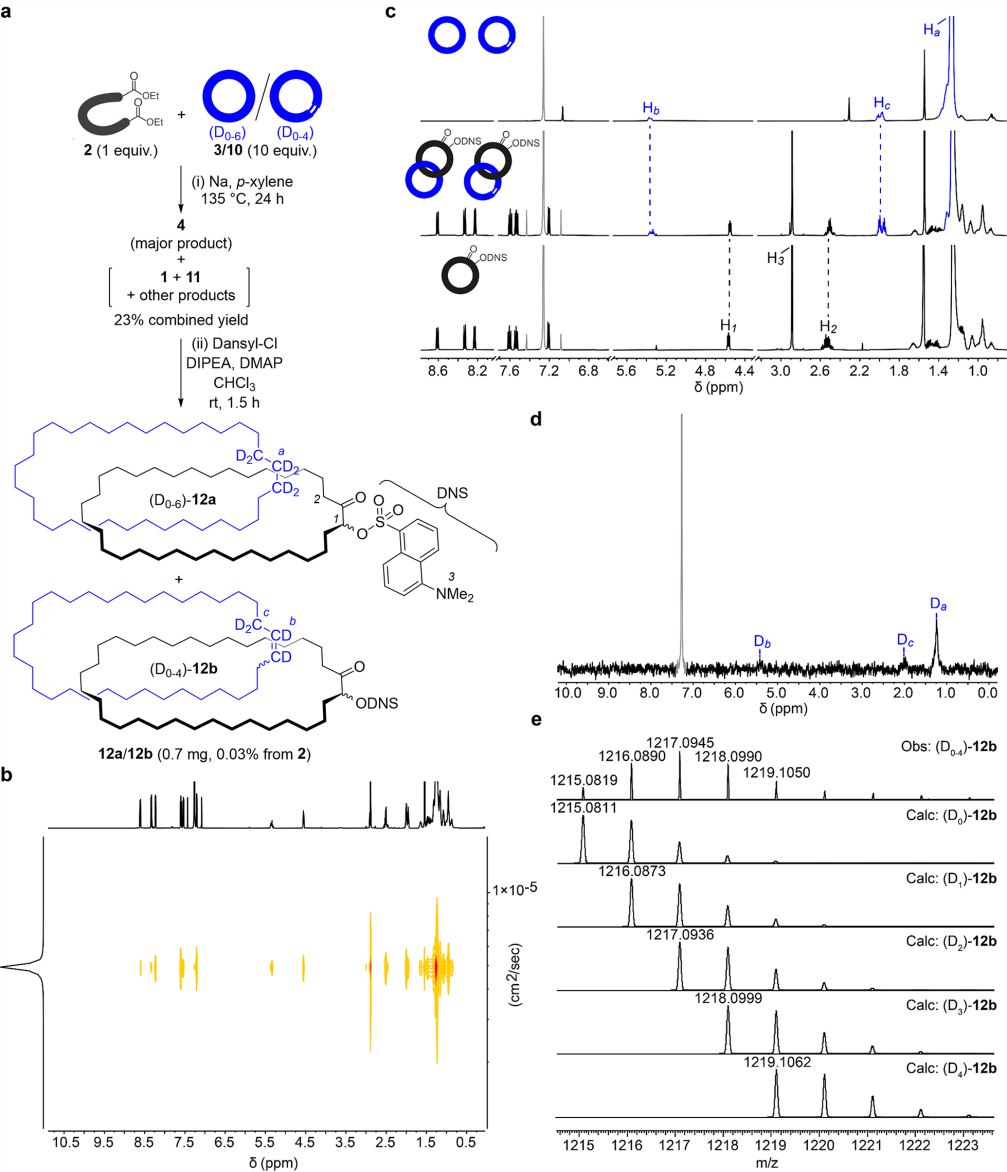
(a) Derivatization of the catenane-forming reaction mixture (after removal of excess **3**/**10**) with dansyl chloride, enabling the isolation of **12**, a derivative of Wasserman’s catenane. (b) DOSY spectrum (600 MHz, CDCl_3_, 298 K) of **12a**/**12b**. (c) Partial ^1^H NMR stackplot (600 MHz, CDCl_3_, 298 K) of (top) deuterium-labeled **3**/**10**; (middle) dansyl-derivatized catenane **12a**/**12b**; (bottom) dansyl-derivatized acyloin. Upper-case letters (deuterium-labeled macrocycle) and lower-case letters (acyloin) correspond to the lettering shown in (a). Signal in gray corresponds to CHCl_3_. (d) ^2^H NMR spectrum (77 MHz, CHCl_3_, 298 K) of **12a**/**12b**. Signal in gray corresponds to CDCl_3_. (e) HRMS (APCI+) mass spectrum of **12b**.

To address these problems, we investigated derivatizing the polar fraction product mixture with fluorescent and UV-active tags. We found that 4-nitrobenzoyl chloride and dansyl chloride both reacted cleanly and in good yields with the hydroxyl group of **4**, forming the corresponding ester and sulfonate ester, respectively (Supporting Information, Sections 7.1 and 7.3). Similar treatment of the catenane-containing polar fraction with either 4-nitrobenzoyl chloride or dansyl chloride, in the presence of *N*,*N*-diisopropylethylamine (DIPEA) and 4-(dimethylamino)pyridine (DMAP) (Figure 4a), gave a mixture of products derivatized with the tags (Supporting Information, Sections 7.2 and 7.4). Chromatography of these derivatives enabled us to identify a number of other byproducts originating from the acyloin macrocyclization reaction, including the macrocyclic acyloin dimer (**S14**, the bis(dansyl sulfonate ester) of **S7**), cyclic alcohols and diols (**S10a**, **S10b**, and **S13**), ketone (**S6**) and diketone (**S5**) adducts,^31^ adducts formed from a Dieckmann-type condensation of **2** (**S12**),^32^ and linear products formed from a Bouveault-Blanc reduction of **2** (**S11**);^33^ see Figures S8 and S10. The formation of these byproducts from the acyloin condensation, many of them macrocyclic, suggests that the catenane-forming reaction does not produce a single interlocked structure but actually a mixture of catenanes that includes several different macrocycles, each interlocked with **3** or **10**.

Following repetitive chromatography of the mixture obtained from dansylation (Figure 4a), we isolated 0.7 mg of a mixture of catenanes **12a** and **12b**. The ^1^H NMR spectrum (600 MHz, CDCl_3_) shows signals from both macrocycles (Figure 4c). The lack of potential magnetic shielding/deshielding groups and well-defined coconformations results in there being essentially no chemical shift differences between protons in the catenane (Figure 4c, middle) and the equivalent protons in the individual noninterlocked components (Figure 4c, top and bottom). Diffusion-ordered NMR spectroscopy (DOSY) shows the acyloin and cycloalkane/ene rings to be part of the same molecular species (Figure 4b). The ^2^H NMR spectrum (77 MHz, CHCl_3_) confirms the presence of deuterium signals with the same δ2_H_ shifts as (D_0–6_)-**3**:(D_0–4_)-**10** (Figure 4d). High resolution mass spectrometry shows the same pattern of deuterium distribution for (D_0–6_)-**12a** as (D_0–6_)-**3** and for (D_0–4_)-**12b** (Figure 4e) as (D_0–4_)-**10**. Tandem MS/MS of the molecular ions of **12a** and **12b** gave *m*/*z* values characteristic of fragmentation of a catenane (Figure S25). The isolated yield of **12a**/**b** is 0.03%, in two steps from **2** (1. Statistical catenane formation by acyloin condensation; 2. Dansylation; Figure 4a).

## Conclusions

Robust, scalable syntheses have been developed for key building blocks in the 10–100 g quantities necessary to reinvestigate Wasserman’s claim of the first catenane synthesis^7^ using the so-called “statistical approach”. With the benefit of modern day synthetic and analytical methods and instrumentation, we were able to demonstrate that Wasserman’s principle claims are essentially correct. Catenane **1** is, indeed, formed by acyloin condensation macrocyclization of diester **2** at a temperature where **3** can be used as a cosolvent with *p*-xylene. Furthermore, the indirect proof-of-structure outlined in the 1960 paper^7^ (that **3** is completely removed from the reaction products by chromatography and is subsequently released from a mixture containing the catenane by oxidation) is experimentally reproducible. Present day tandem mass spectrometry provided direct and unequivocal proof of the interlocked catenane structure from the reinvestigated reaction.

Deviations from Wasserman’s findings include that ^2^H NMR spectroscopy shows that a substantial amount of alkene macrocycle is present in the deuterium-labeled cycloalkane prepared by the Clemmensen reduction used by both Wasserman and ourselves. The presence of this macrocyclic alkene—and other macrocyclic byproducts identified from the acyloin condensation—means that a number of different [2]catenanes are actually produced in the catenane-forming reaction; it is not just **1**. Wasserman did not report detailed experimental procedures, but we were able to find conditions for the statistical catenane-forming reaction where 0.07% of the diester starting material (**2**) becomes interlocked in various catenanes. This corresponds to ∼1 mg of catenane being formed from 1 g of **2** and 10 g of **3**/**10** and is roughly a tenth of the highest yields (0.0001%,^8^ 0.7%,^10^ ∼1%^7^) ascribed to the catenane-forming reaction by Wasserman.

Derivatization of the semipurified product mixture with a fluorescent dansyl sulfonate tag enabled us to isolate a mixture of the dansyl cycloalkane-acyloin and cycloalkene-acyloin catenanes, (D_0–6_)-**12a** and (D_0–4_)-**12b**, in 0.03% overall yield (catenane formation followed by dansyl derivatization) from **2**. ^1^H and ^2^H NMR spectroscopy, DOSY spectroscopy, and mass spectrometry unambiguously demonstrated the interlocked structure of the isolated [2]catenane, **12a**/**b**.

The controversy surrounding Wasserman’s seminal catenane synthesis is thus resolved. Catenane **1** is not a prophetic compound^11^ but was genuinely synthesized (as a mixture of catenanes with **4** and other byproducts) in 1960 by the statistical approach. Furthermore, the evidence used to claim the interlocked structure at that time, albeit indirect, is actually experimentally robust. In our view, this outcome does not detract from the pioneering concepts to direct mechanically interlocked molecule synthesis introduced by Lüttringhaus and Schill.^13^ They inspired Sauvage’s metal template approach^5^ which, in turn, inspired Stoddart’s self-assembly strategies.^6^ Each was a step on a path that ultimately led to the award of a Nobel Prize, not (at least, not formally) for the synthesis of catenanes and rotaxanes,^11^ but for contributions to the nascent field of artificial molecular machinery.^12^

## Supporting Information Available

Experimental procedures, synthesis and characterization data, NMR, MS, and X-ray crystallography data (PDF)
