# Supplementary material for: In Search of Wasserman’s Catenane
Source: J Am Chem Soc. 2023 Apr 25;145(17):9825–33. doi: 10.1021/jacs.3c01939 (PMC10161206; doi:10.1021/jacs.3c01939)
Supplement: Supplementary file 1 — ja3c01939_si_001.pdf [file ja3c01939_si_001.pdf]

-Supporting Information-

In Search of Wasserman’s Catenane

Andrei S. Baluna, Albano Galan, David A. Leigh,* Gareth D. Smith, Justin T. J. Spence, Daniel J. Tetlow, Iñigo J. Vitorica-Yrezabal and Min Zhang

Department of Chemistry, University of Manchester, Oxford Road, Manchester, M13 9PL, UK.

School of Chemistry and Molecular Engineering, East China Normal University, 200062 Shanghai, China.

*E-mail: david.leigh@manchester.ac.uk

Table of contents

1. Abbreviations........ 3
2. General Information........ 4
3. Synthetic schemes........ 5
4. Experimental procedures and data........ 7
5. Procedure for the attempted synthesis of the Wasserman catenane **1**........ 16
6. Oxidation experiments........ 18
    6.2. Oxidative cleavage of the polar fraction obtained from the catenane reaction........ 18
    6.2. Macrocycle removal control oxidation experiment........ 20
7. Tagging experiments........ 21
    7.1. Tagging of acyloin **4** with 4-nitrophenyl chloride........ 21
    7.2. Attempt to form Wasserman’s catenane, **1**, with subsequent tagging of the crude product mixture with 4-nitrophenyl chloride........ 22
    7.3. Tagging of acyloin **4** with dansyl chloride........ 25
    7.4. Isolation of **12a**/**12b** aided by tagging with dansyl chloride........ 26
8. MS spectra........ 29
    8.1. HRMS (APCI+) spectra of deuterated macrocycle **3**/**10**........ 29
    8.2. HRMS (APCI+) spectrum of acyloin **4**........ 31
    8.3. HRMS (APCI+) spectra of the mixture of polar products isolated from the catenane-forming reaction........ 32
    8.4. HRMS (APCI+) spectra of the isolated dansyl-catenane mixture **12a**/**b**........ 34
9. NMR spectra........ 37
10. X-ray crystallography data........ 59
    8.1. X-ray data of acyloin **4**........ 59
11. References........ 61

1. Abbreviations

APCI atmospheric pressure chemical ionization; bipy 2,2′-bipyridine; calcd. calculated; CAM cerium ammonium molybdate; conc. concentrated; d day/s; D-mac deuterated macrocycle; Dansyl 5-(Dimethylamino)naphthalene-1-sulfonyl chloride; DIPEA diisopropylethylamine; DMAP (4-dimethylamino)pyridine; DMSO dimethylsulfoxide; DNS Dansyl; DOSY Diffusion-ordered NMR spectroscopy; Et ethyl; EtOAc ethyl acetate; ESI electrospray ionization; h hour/s; HPLC high performance liquid chromatography; HRMS high resolution mass spectrometry; IR infrared; KHMDS potassium bis(trimethylsilyl)amide; LRMS low resolution mass spectrometry; Me methyl; min minute/s; m.p. melting point; MS mass spectrometry/spectrum/spectra; NMI *N*-methylimidazole; NMR nuclear magnetic resonance; Ph phenyl; rt room temperature; sat. aq. saturated aqueous; TEMPO (2,2,6,6-tetramethyl-piperidin-1-yl)oxidanyl; TES-H triethylsilane; THF tetrahydrofuran; TLC thin layer chromatography; TMSCl trimethylsilyl chloride.

2. General information

All reagents and solvents were purchased from commercial sources and used without further purification. Anhydrous THF, CH_2Cl_2, CH_3CN and toluene were obtained by passing the solvent (HPLC grade) through an activated alumina column on a Phoenix SDS solvent drying system (JC Meyer Solvent Systems, CA, USA). All reactions were performed using flame-dried glassware under an atmosphere of N_2, unless stated otherwise. Column chromatography was carried out using Aldrich Si 60 (particle size 40-63μm) as the stationary phase, while TLC was performed on precoated silica gel plates (0.2 mm thick, 60 F_{254}, Macherey-Nagel, Germany) and visualized using Ceric Ammonium Molybdate (CAM) stain. Size-exclusion chromatography was carried out under gravity using a neutral, porous styrene divinylbenzene resin (1% crosslinked linked, Bio-Rad, Bio-Beads, S-X1) as stationary phase and $CHCl_3$ as an eluent. ^1H NMR spectra were recorded on a Bruker Avance III instrument with an Oxford AS600 magnet equipped with a cryoprobe [5mm CPDCH 13C-1H/D] (600 MHz) at 298 K. Chemical shifts are reported in parts per million (ppm) from high to low frequency using the residual solvent peak as the internal reference ($CDCl_3$ = 7.26 ppm). All ^1H resonances are reported to the nearest 0.01 ppm. The multiplicity of ^1H signals are indicated as: s = singlet; d = doublet; t = triplet; q = quartet; p = quintet; m = multiplet; br = broad; or combinations of thereof. Coupling constants (*J*) are quoted in Hz and reported to the nearest 0.1 Hz. Where appropriate, averages of the signals from peaks displaying multiplicity were used to calculate the value of the coupling constant. ^{13}C NMR spectra were recorded on the same spectrometer at 298 K with the central resonance of the solvent peak as the internal reference ($CDCl_3$ = 77.16 ppm). All ^{13}C resonances are reported to the nearest 0.1 ppm in general, or to 0.01 ppm to aid in the differentiation of close but resolved signals. ^2H NMR spectra were recorded on a Bruker AVIII HD 500 equipped with a prodigy BBO 5 mm probe, with the central resonance of the solvent peak as the internal reference ($CHCl_3$ = 7.26 ppm). All ^2H resonances are reported to the nearest 0.01 ppm. DEPT, COSY, HSQC and HMBC experiments were used to aid structural determination and spectral assignment. Low resolution ESI mass spectrometry was performed with a Thermo Scientific LCQ Fleet Ion Trap Mass Spectrometer or an Agilent Technologies 1200 LC system with either an Agilent 6130 single quadrupole MS detector or an Advion Expression CMS L single quadrupole MS detector. High resolution (ESI, APCI) mass spectrometry was carried out by the Department of Chemistry, University of Manchester. Infrared spectra were recorded neat on a Bruker Alpha II Platinum ATR. Melting points (m.p.) were determined using a Büchi Melting Point M-565.

3. Synthetic schemes

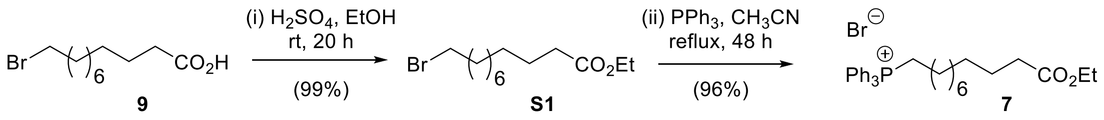

Scheme S1. Synthesis of phosphonium bromide **7**.

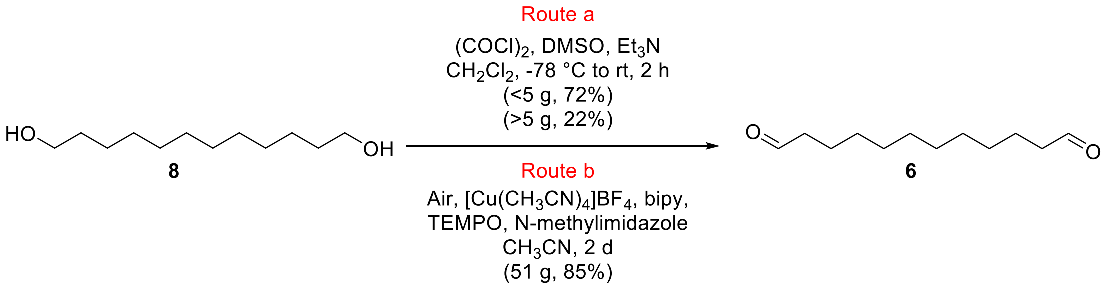

Scheme S2. Synthesis of dialdehyde **6**.

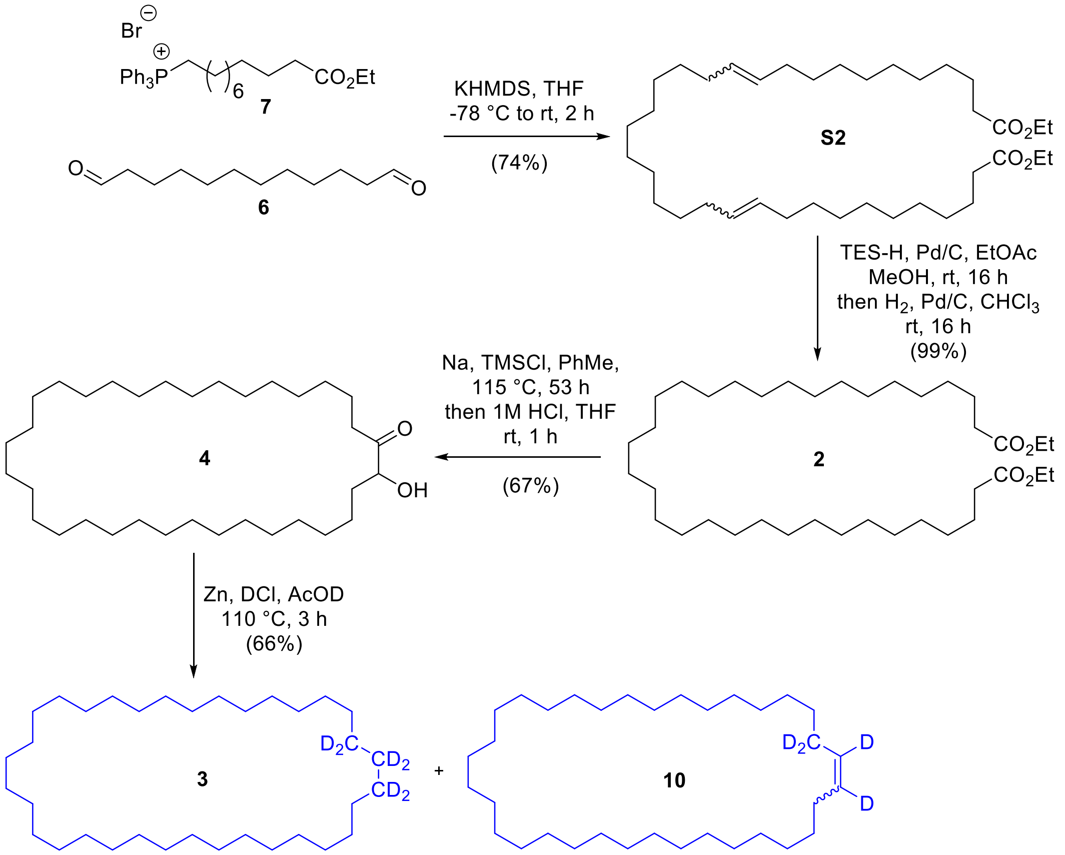

Scheme S3. Synthesis of acyloin **4** and deuterated macrocycles **3** and **10**.

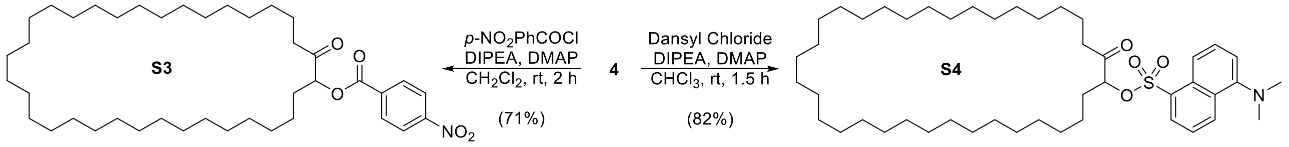

Scheme S4. Synthesis of tagged acyloins **S3** and **S4**

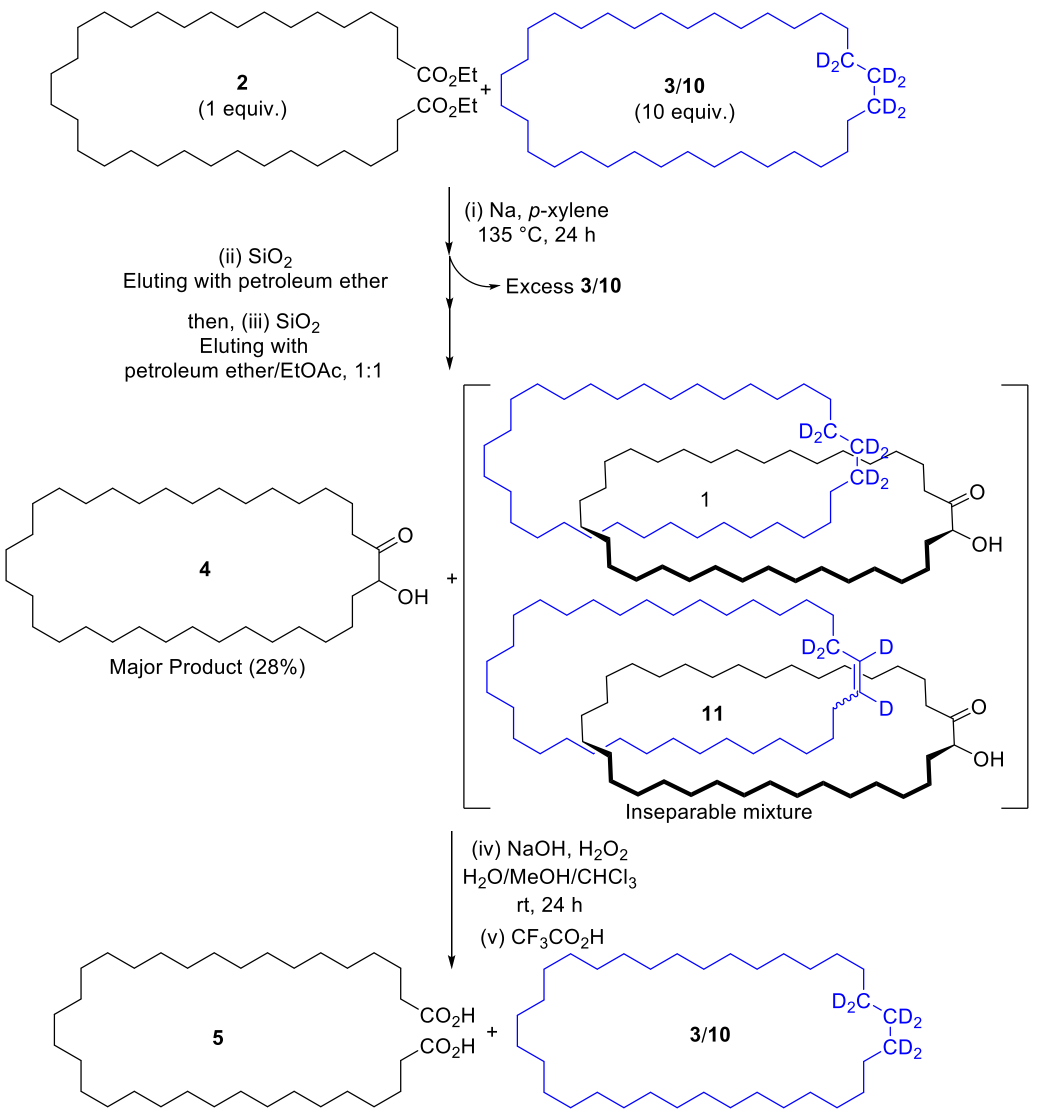

Scheme S5. Synthesis of a [2]catenane by statistical threading of alkyl diester through a deuterated macrocycle during an acyloin condensation

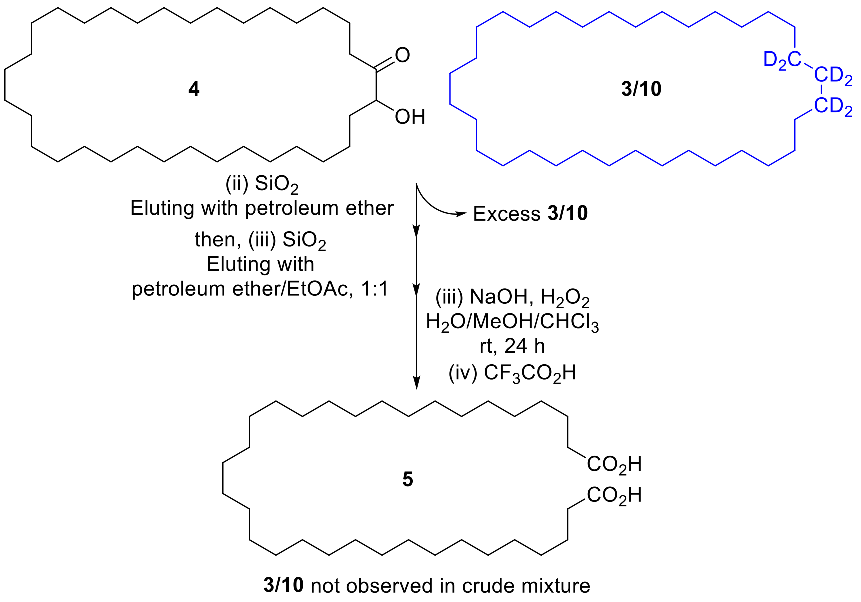

Scheme S6. Control experiment to show that filtration with petroleum ether as an eluent is effective at removing the excess deuterated macrocycle from the mixture prior to oxidation with alkaline H_2O_2.

4. Experimental procedures and data

Ethyl 11-bromoundecanoate (**S1**)

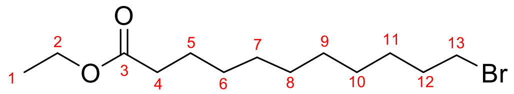

11-bromo undecanoic acid (10.0 g, 37.7 mmol) was dissolved in EtOH (200 mL) and conc. H_2SO_4 (10 mL) was added. The mixture was stirred at room temperature for 20 hours and the reaction was quenched with sat. aq. $NaHCO_3$. The product was extracted with CH_2Cl_2 (3 × 100 mL). The combined organic extracts were washed with brine, dried over anhydrous $MgSO_4$, filtered and the solvent was evaporated under reduced pressure. **S1** was obtained as a colorless oil (10.94 g, 37.5 mmol, 99%), which was sufficiently pure to be used without further purification. Experimental data consistent with literature.[1]

^{1}H NMR (600 MHz, 298 K, $CDCl_3$) δ 4.11 (q, *J* = 7.2 Hz, 2H, H_2), 3.40 (t, *J* = 7.0 Hz, 2H, H_{13}), 2.28 (t, *J* = 7.7, 1.6 Hz, 2H, H_4), 1.84 (p, *J* = 7.1 Hz, 2H, H_{12}), 1.61 (p, *J* = 7.7 Hz, 2H, H_5), 1.40 (p, *J* = 7.1 Hz, 2H, H_{11}), 1.28 (s, 10H, $H_{6\text{-}10}$), 1.25 (t, *J* = 7.2 Hz, 3H, H_1).

^{13}C NMR (151 MHz, 298 K, $CDCl_3$) δ 173.9 (C_3), 60.1 (C_2), 34.3 (C_4), 34.0 (C_{13}), 32.8 (C_{12}), 29.3, 29.28, 29.2, 29.1 ($C_{6\text{-}9}$), 28.7, 28.1 ($C_{10\text{-}11}$), 24.9 (C_5), 14.2 (C_1).

HRMS (+ESI) m/z: calcd. for $C_{13}H_{25}{}^{79}BrO_2Na$ $[M+Na]^+$ 315.0930, found 315.0923; calcd. for $C_{13}H_{25}{}^{81}BrO_2Na$ $[M+Na]^+$ 317.0910, found 317.0903.

IR (neat) ν_{max}/cm^{-1}: 2925, 2854, 1733, 1463, 1371, 1244, 1176, 1115, 1034, 857, 722, 644, 562.

(11-ethoxy-11-oxoundecyl)triphenylphosphonium bromide (**7**)

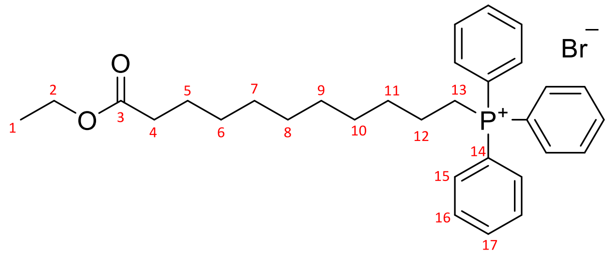

In a flame-dried flask, ethyl 11-bromoundecanoate **S1** (10.94 g, 37.3 mmol) and PPh_3 (10.3 g, 39.2 mmol) were added and dissolved in dry CH_3CN (60 mL). The solution was heated at reflux for 48 hours. The solvent was evaporated under reduced pressure and the crude oil obtained was washed with Et_2O (3 x 100 mL). The resultant residue was thoroughly dried at 120 °C overnight under high vacuum to give **7** (19.93 g, 35.9 mmol, 96%) as a viscous yellow oil, which was used in the next step without further purification. Experimental data was consistent with literature.[2]

^{1}H NMR (600 MHz, 298 K, $CDCl_3$) δ 7.86 (ddd, *J* = 12.6, 7.8, 1.5 Hz, 6H, H_{15}), 7.78 (ddd, *J* = 7.8, 5.3, 1.5 Hz, 3H, H_{17}), 7.70 (td, *J* = 7.8, 3.3 Hz, 6H, H_{16}), 4.10 (q, *J* = 7.1 Hz, 2H, H_2), 3.93 – 3.80 (m, 2H, H_{13}), 2.25 (t, *J* = 7.5 Hz, 2H, H_4), 1.64 – 1.60 (m, 4H, $H_{11,12}$), 1.57 (p, *J* = 7.5 Hz, 2H, H_5), 1.24 (t, *J* = 7.1 Hz, 3H, H_1), 1.21 – 1.18 (s, br, 10H, H_{6-10}).

^{13}C NMR (151 MHz, 298 K, $CDCl_3$) δ 173.7 (C_3), 134.7 (d, *J* = 3.0 Hz, C_{17}), 133.5 (d, *J* = 10.0 Hz, C_{15}), 130.2 (d, *J* = 12.5 Hz, C_{16}), 118.3 (d, *J* = 85.6 Hz, C_{14}), 59.9 (C_2), 34.12 (C_4), 30.1 (d, J_{C-P} = 15.5 Hz, C_{11}), 29.04, 29.00, 28.9, 28.85, 28.81, 24.7 (C_{5-10}), 22.6 (d, J_{C-P} = 34.6 Hz, C_{13}), 22.4 (d, J_{C-P} = 10.4 Hz, C_{12}), 14.0 (C_1).

^{31}P NMR (162 MHz, 298 K, $CDCl_3$) δ 24.5 (s).

HRMS (+ESI) m/z: calcd. for $C_{31}H_{40}O_2P$ $[M-Br]^+$ 475.2760, found 475.2752.

IR (neat) ν_{max}/cm^{-1}: 3361, 3052, 2984, 2925, 1725, 1436, 1247, 1182, 747, 721, 507, 494.

Dodecanedial (**6**)

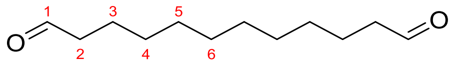

In a flame-dried flask, oxalyl chloride (5.0 mL, 59.2 mmol) was dissolved in anhydrous CH_2Cl_2 (50 mL) and the solution was cooled to –78 °C. Anhydrous DMSO (5.3 mL) was added dropwise, making sure the reaction temperature did not exceed –60 °C. 1,12-dodecanediol (3.0 g, 14.8 mmol) was dissolved in a 1:1 mixture of CH_2Cl_2/DMSO (30 mL) and was added to the reaction flask *via* cannula. After 15 minutes of stirring at –78 °C, Et_3N (20.0 mL) was added and the solution was allowed to gradually warm to room temperature where it was stirred for 2 hours. The reaction was quenched with water (100 mL) and extracted with CH_2Cl_2 (3 × 100 mL). The combined organic phases were washed with brine, dried over anhydrous $MgSO_4$, filtered and the solvent was evaporated under reduced pressure. Purification by flash chromatography (SiO_2, petroleum ether/EtOAc, 4:1) gave **6** as a colorless solid (2.1 g, 10.6 mmol, 72%). Experimental data was consistent with literature.[3]

m.p. 36 – 38 °C.

^{1}H NMR (600 MHz, 298 K, $CDCl_3$) δ 9.76 (t, *J* = 1.9 Hz, 2H, H_1), 2.42 (td, *J* = 7.4, 1.9 Hz, 4H, H_2), 1.62 (p, *J* = 7.4 Hz, 4H, H_3), 1.35 – 1.25 (m, 12H, $H_{4\text{-}6}$).

^{13}C NMR (151 MHz, 298 K, $CDCl_3$) δ 202.8 (C_1), 43.7 (C_2), 29.1, 29.0 ($C_{5\text{-}6}$), 28.9 (C_4), 21.8 (C_3).

HRMS (+APCI) m/z: calcd. for $C_{12}H_{23}O_2$ $[M+H]^+$ 199.1693, found 199.1691.

IR (neat) v_{max}/cm^{-1}: 2923, 2912, 2848, 2747, 1708, 1470, 1409, 1391, 1074, 993, 698, 477.

For larger scale synthesis of **6** (up to 50 g) a modified version of a previously reported procedure was used.[4] To a solution of 1,12-dodecanediol (51.1 g, 252.5 mmol) in anhydrous acetonitrile (1.5 L), was added $[Cu(MeCN)_4]BF_4$ (5.6 g, 17.8 mmol), 2,2’-bipyridine (2.8 g, 17.8 mmol), TEMPO (2.8 g, 17.8 mmol) and *N*-methylimidazole (2.8 mL, 35.8 mmol). The flask was vigorously stirred under air at room temperature for 2 days. Over the course of the reaction, the color of the mixture transitioned from dark red to blue. The crude reaction mixture was filtered over a large pad of celite and the filtrate was concentrated under reduced pressure to afford **6** (42.5 g, 214.6 mmol, 85%) as a colorless solid, which used without further purification.

Diethyl tetratriaconta-11,23-dienedioate (**S2**)

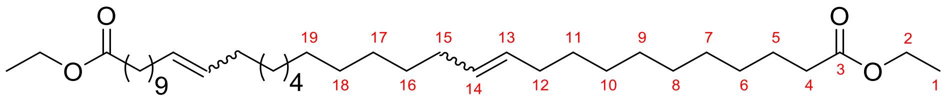

Phosphonium salt **7** (19.9 g, 35.8 mmol) was dissolved in anhydrous THF (300 mL) and cooled to -78 °C. KHMDS solution (1.0 M in THF, 36.0 mL, 36.0 mmol) was added dropwise. After 20 min, a solution of dialdehyde **6** (3.4 g, 17.1 mmol) in anhydrous THF (30 mL) was added dropwise, over 10 min, while keeping the internal reaction temperature at –78 °C. After the complete addition of **6**, the reaction mixture was allowed to warm to room temperature and stirred for 2 hours. The excess ylide was quenched with EtOH and the solvent was evaporated under reduced pressure. The resulting crude solid was redissolved in CH_2Cl_2 (500 mL) and water (500 mL) was added. The two phases were separated, and the aqueous phase was further extracted with CH_2Cl_2 (2 × 250 mL). The organic phases were combined, washed with brine (2 × 250 mL), dried over anhydrous $MgSO_4$, filtered and the solvent was evaporated under reduced pressure. Purification by flash chromatography (SiO_2, petroleum ether/EtOAc, 15:1) yielded **S2** (7.56 g, 12.8 mmol, 74%) as a colorless oil.

^{1}H NMR (600 MHz, 298 K, $CDCl_3$) δ 5.39 – 5.31 (m, 4H, $H_{13,14}$), 4.12 (q, J = 7.1 Hz, 4H, H_2), 2.28 (t, J = 7.5 Hz, 4H, H_4), 2.01 (q, J = 6.8 Hz, 8H, $H_{12,15}$), 1.61 (p, J = 7.5 Hz, 4H, H_5), 1.37 – 1.27 (m, 40H, $H_{6\text{-}11,16\text{-}19}$), 1.25 (t, J = 7.1 Hz, 6H, H_1).

^{13}C NMR (151 MHz, 298 K, $CDCl_3$) δ 173.7 (C_3), 129.7 (C_{13}), 129.6 (C_{14}), 59.9 (C_2), 34.2 (C_4), 29.6, 29.5, 29.4, 29.3, 29.2, 29.1, 29.0, 28.9 ($C_{6\text{-}11}$, $C_{15\text{-}19}$), 27.0 (C_{12}), 26.98 (C_{15}), 24.8 (C_5), 14.0 (C_1).

HRMS (+ESI) m/z: calcd. for $C_{38}H_{70}O_4Na$ $[M+Na]^+$ 613.5166, found 613.5154

IR (neat) ν_{max}/cm^{-1}: 3001, 2977, 2922, 2852, 1736, 1463, 1371, 1247, 1177, 1035, 721.

Diethyl tetratriacontanedioate (**2**)

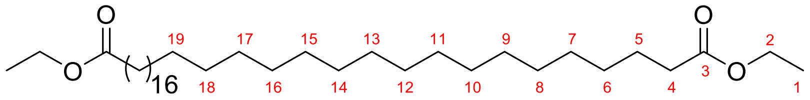

Dialkene **S2** (52 g, 88 mmol) was dissolved in ethyl acetate (300 mL) and methanol (300 mL). Under an argon atmosphere, 10% Pd/C (5g, 1% wt.) was added in small portions. Triethylsilane (80 mL, 5 eq.) was added dropwise, and the reaction stirred overnight at room temperature. Over time, the fully saturated diester crushed out of solution. The mixture obtained was filtered over a large pad of celite and washed with ethyl acetate to remove impurities. The celite pad was then washed with chloroform (400 mL) to elute a mixture of unreacted starting material **S2** and desired compound **2** in a *ca.* 1:9 ratio. To this, 10% Pd/C (500 mg) was added, and the resulting suspension was stirred overnight at room temperature under an atmosphere of H_2. The suspension was filtered through a pad of celite and the solvent was evaporated under reduced pressure to yield **2** (52 g, 7.5 mmol, 99%) as a colorless solid, which was used without further purification.

m.p. 82 – 83 °C.

^{1}H NMR (600 MHz, 298 K, $CDCl_3$) δ 4.12 (q, J = 7.1 Hz, 4H, H_2), 2.28 (t, J = 7.5 Hz, 4H, H_4), 1.61 (p, J = 7.5 Hz, 4H, H_5), 1.33 – 1.21 (m, 62H, $H_{1,6\text{-}19}$).

^{13}C NMR (151 MHz, 298 K, $CDCl_3$) δ 173.7 (C_3), 59.9 (C_2), 34.2 (C_4), 29.5, 29.4, 29.3, 29.2, 29.0, 28.9 ($C_{6\text{-}19}$), 24.8 (C_5), 14.0 (C_1).

HRMS (+APCI) m/z: calcd. for $C_{38}H_{75}O_4$ $[M+H]^+$ 595.5660, found 595.5660.

IR (neat) ν_{max}/cm^{-1}: 2976, 2915, 2847, 1736, 1462, 1379, 1285, 1244, 1183, 1172, 730, 719.

2-hydroxycyclotetratriacontan-1-one (**4**)

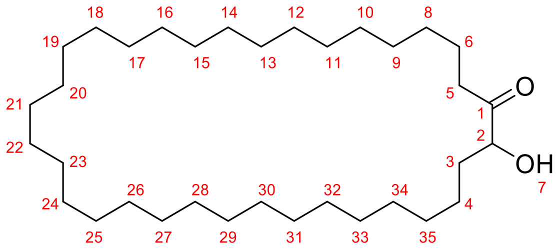

Method 1. In a flame-dried flask, finely cut sodium pieces (~100 mg, 4.2 mmol) were added to anhydrous toluene (25 mL) and the reaction mixture was stirred vigorously under reflux to generate a sodium dispersion.[a] To this, a solution of diester **2** (500 mg, 0.84 mmol) and TMSCl (2.5 mL, 19.8 mmol) in dry toluene (25 mL) was added dropwise *via* an addition funnel over 3 hours.[b] After the complete addition of the diester, the reaction mixture was stirred at 115 °C for 20 hours. The mixture was cooled to room temperature and the solution was quenched with methanol (5 mL) and then aq. 1M HCl (5 mL). The suspension was filtered through a pad of celite, and the filtrate was evaporated under reduced pressure to give a yellow grease. The grease was redissolved in a mixture of THF (15 mL) and aq. 1M HCl (2 mL), and the solution was stirred at room temperature for 1 hour. The product was extracted with $CHCl_3$ (3 × 20 mL) and the combined organic phases were washed with sat. aq. $NaHCO_3$, brine and dried over anhydrous $MgSO_4$. The solvent was removed under reduced pressure and the crude product obtained was purified by flash column chromatography (SiO_2, petroleum ether/EtOAc, 9:1) to yield **4** (201 mg, 0.40 mmol, 47%) as a colorless solid. Single crystals of **4** suitable for X-ray crystallography were obtained by slow diffusion of ^{i}PrOH into a solution of **4** in $CHCl_3$.

m.p. 67 – 69 °C.

^{1}H NMR (600 MHz, 298 K, $CDCl_3$) δ 4.18 (dt, *J* = 7.1, 4.3 Hz, 1H, H_2), 3.51 (d, *J* = 4.4 Hz, 1H, H_7), 2.50 (ddd, *J* = 16.9, 8.7, 6.5 Hz, 1H, H_5), 2.39 (ddd, *J* = 16.9, 8.7, 6.0 Hz, 1H, $H_{5'}$), 1.82 (dtd, *J* = 13.6, 9.9, 4.3 Hz, 1H, H_3), 1.70 – 1.62 (m, 1H, H_6), 1.63 – 1.56 (m, 1H, $H_{6'}$), 1.58 – 1.51 (m, 1H, $H_{3'}$), 1.50 – 1.41 (m, 1H, H_4), 1.38 – 1.31 (m, 1H, $H_{4'}$), 1.31 – 1.23 (m, 56H, $H_{8\text{-}35}$).

^{13}C NMR (151 MHz, 298 K, $CDCl_3$) δ 212.6 (C_1), 76.2 (C_2), 37.7 (C_5), 33.6 (C_3), 30.6 – 27.5 ($C_{8\text{-}35}$), 24.4 (C_4), 23.5 (C_6).

HRMS (+APCI) m/z: calcd. for $C_{34}H_{67}O_2$ $[M+H]^+$ 507.5136, found 507.5137.

IR (neat) ν_{max}/cm^{-1}: 3433 (br), 2911, 2847, 1710, 1470, 1122, 715, 619.

[a] We found that the yield of the reaction was strongly dependent on the quality of the sodium dispersion formed. It is vital that the sodium gets finely dispersed before addition of the diester and this is best achieved by mechanical stirring. For the scale-up of the reaction, it was decided to use pre-dispersed Na in toluene.
[b] The solubility of the diester **2** in cold toluene is poor, so regular heating of the addition funnel is required.

Method 2. In a flame-dried flask equipped with a condenser and a 500 mL heated addition funnel, anhydrous toluene (100 mL) was added (see Fig. S1 for the reaction set-up). To this, 30% sodium dispersion in toluene (6 mL, ~1.8 g sodium, 78 mmol) was added and the reaction flask was brought to 115 °C under vigorous stirring. Diester **2** (7.0 g, 11.8 mmol) and anhydrous toluene (150 mL) were transferred to the addition funnel, which was heated to 40 °C to allow for the solvation of the poorly soluble diester. TMSCl (26.5 mL, 22.8 g, 210 mmol) was added to the diester solution and the resulting mixture was added dropwise to the main reaction flask. The addition was completed over 48 hours, during which time the reaction flask was kept at 115 °C and the addition funnel was kept at 40 °C. After the addition was complete, the mixture was stirred for further 5 hours and then cooled to room temperature. MeOH (10 mL) was added dropwise to quench the excess sodium, followed by the addition of aq. 1M HCl (20 mL). The suspension was filtered through a pad of celite, and the filtrate was evaporated under reduced pressure to give a yellow grease. The grease was redissolved in a mixture of THF (150 mL) and aq. 1M HCl (20 mL), and the solution was stirred at room temperature for 1 hour. The product was extracted with $CHCl_3$ (3 × 200 mL) and the combined organic phases were washed with sat. aq. $NaHCO_3$, brine and dried over anhydrous $MgSO_4$. The solvent was removed under reduced pressure and the crude product obtained was purified by flash column chromatography (SiO_2, petroleum ether/EtOAc, 9:1) to yield **4** (4.1 g, 8.1 mmol, 67%) as a colorless solid.

Note: Spectroscopic data was identical to that obtained *via* Method 1.

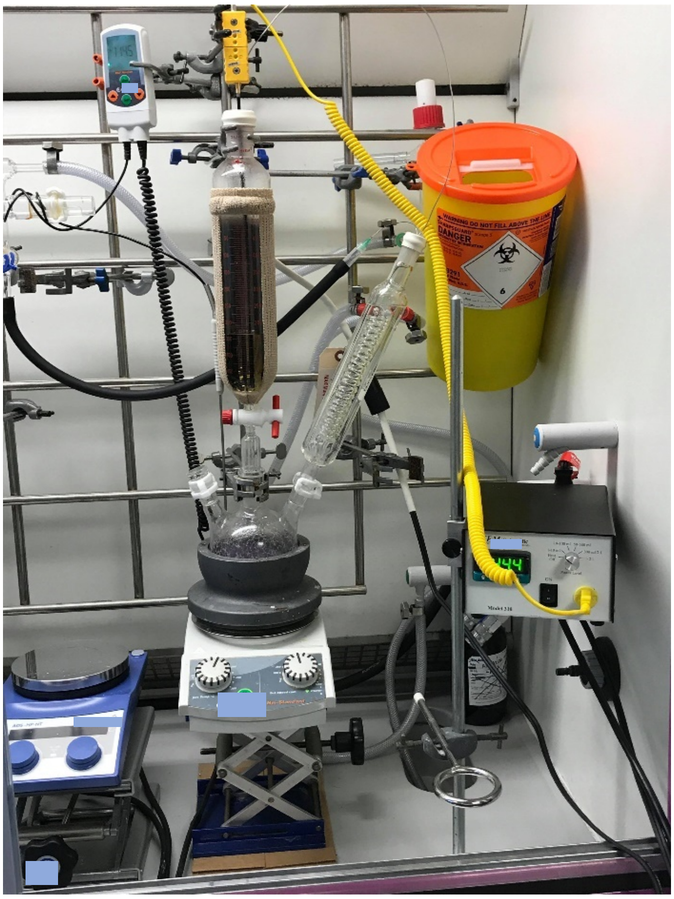

Figure S1. Acyloin reaction apparatus set-up with heated addition funnel.

Several additional by-products from the acyloin reaction (Fig. S2) were also isolated and characterized (see Section 9 for full NMR analysis).

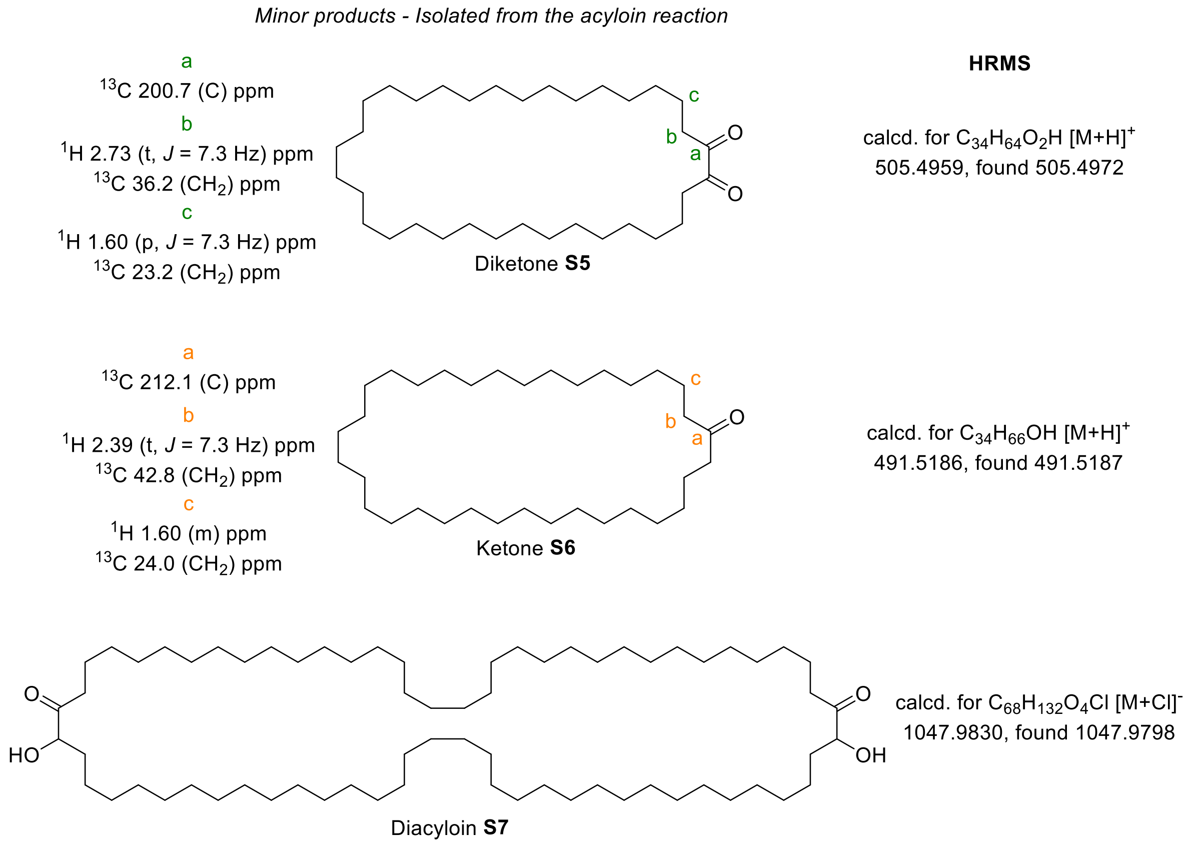

Figure S2. Acyloin reaction by-products.

Cyclotetratriacontane/ene (**3/10**)

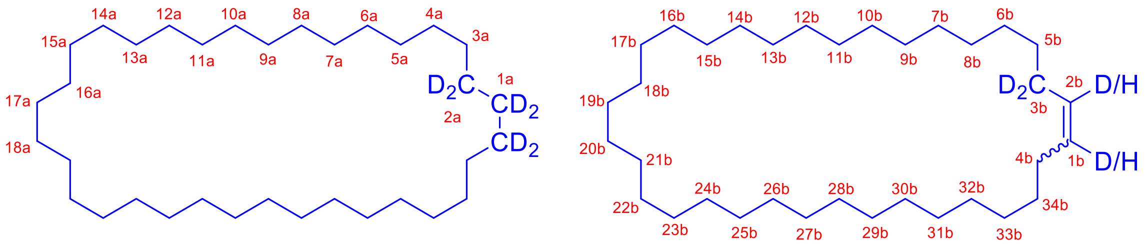

Acyloin **4** (2.7 g, 5.33 mmol) was suspended in a mixture of conc. DCl (35% in D_2O, 40 mL) and deuterated acetic acid (25 mL). Zinc dust (20 g, 306 mmol) was added in small portions and the reaction was stirred at 110 °C for 3 hours. The reaction was cooled to room temperature and extracted with petroleum ether (2 × 20 mL) and $CHCl_3$ (3 × 20 mL). The combined organic phases were washed with brine, dried over anhydrous $MgSO_4$, filtered, and concentrated under reduced pressure. Purification by flash column chromatography (SiO_2, petroleum ether) gave an inseparable mixture of **3** and **10** (1.7 g, 3.54 mmol, 66%).[c]

m.p. 61 – 63 °C.

^{1}H NMR (600 MHz, 298 K, $CDCl_3$) δ 5.42 – 5.32 (m, $H_{1b,2b}$), 2.05 – 1.93 (m, $H_{3b,4b}$), 1.27 (s, $H_{3a\text{-}18a,5b\text{-}34b}$).

^{13}C NMR (151 MHz, 298 K, $CDCl_3$) δ 130.6 – 130.0 ($C_{1b,2b}$), 32.6 – 32.5 ($C_{3b/4b}$), 29.4 ($C_{1a\text{-}18a,5b\text{-}34b}$), 27.2 – 27.1 ($C_{4b/3b}$).

^{2}H NMR (77 MHz, 298 K, $CHCl_3$) δ 5.41 (br s, $D_{1b,2b}$), 1.98 (br s, D_{3b}), 1.24 (br s, $D_{1a,2a}$).

HRMS (+APCI) m/z: calcd. for $C_{34}H_{61}D_4$ $[M\text{-}H]^+$ 477.5332, found 477.5335 (**10**); calcd. for $C_{34}H_{61}D_6$ $[M\text{-}H]^+$ 481.5614, found 481.5611 (**3**). (see Section 8.1, Fig. S12–S15).[5,6]

IR (neat) ν_{max}/cm^{-1}: 2912, 2847, 2189, 2087, 1470, 715.

[c] The isolated colorless solid is a mixture of compounds, saturated and unsaturated, with different degrees of deuteration ranging from 0 to 6 deuterium atoms. The highest peaks in the mass spectrum correspond to macrocycles containing 4 and 6 deuteriums, respectively.

5. Procedure for the attempted synthesis of the Wasserman catenane (1)

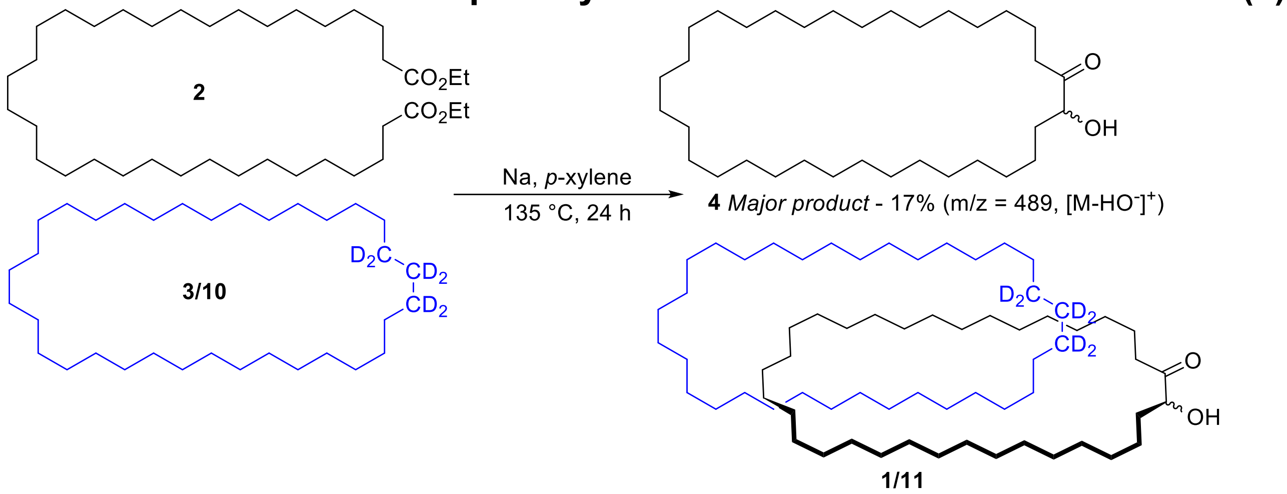

Observed as a minor component by APCI MS (m/z = 964 - 972, [M-HO$^-$]$^+$)

Scheme S7. Acyloin condensation in the presence of a large excess of deuterated cyclotetratriacontane/ene.

In a flame-dried flask, **3**/**10** (10.0 g, 20.8 mmol) was added and the flask was heated to 90 °C. After the solid had melted, anhydrous *p*-xylene (5.0 mL) was added, followed by the addition of 30% sodium dispersion in toluene (1.5 mL dispersion, ~450 mg sodium, 19.6 mmol). Diester **2** (1.0 g, 1.68 mmol) was added and the reaction was stirred at 135 °C for 24 hours. The reaction was allowed to cool to room temperature and $CHCl_3$ (50 mL) was added to solubilize the solid mass formed. The excess sodium was carefully quenched by addition of MeOH (5 mL) and aq. 1M HCl (10 mL). The product was extracted with $CHCl_3$ (3 × 200 mL), and the combined organic phases were washed with brine and dried over anhydrous $MgSO_4$.[d] The solvent was removed under reduced pressure and the crude obtained was purified by flash column chromatography (SiO_2, petroleum ether, then petroleum ether/EtOAc, 1:1) to yield **3**/**10** (9.8 g) and a mixture of polar compounds[e] (236 mg, 28%), respectively.[f]

^{2}H NMR (77 MHz, 298K, $CHCl_3$) of the polar fraction obtained showed the characteristic peaks for the deuterated macrocycles **3**/**10** (Fig. S4), while mass spectrometry analysis of the mixture revealed the presence of peaks corresponding to fragments of the catenated species **1**/**11** (*m/z* = 964 to 972, ([**1**/**11**-H_2O]H^+), see Fig. S17–S18. Further fragmentation of these peaks by MS/MS (see Section 8.3, Fig. S19–S20) generated a distribution of peaks corresponding to the deuterated macrocyclic species **3**/**10** (*m/z* = 473 to 481, [**3**/**10**-H]$^+$) and acyloin **4** (*m/z* = 491, 489 and 474), see Fig. S19 for predicted fragment structures.

[d] Additionally, an insoluble off-white solid (~600 mg, 70%) was recovered from the reaction mixture by filtration. This is believed to be a polymeric acyloin species, based on the IR spectrum which shows acyloin diagnostic peaks (3400 and 1710 cm^{-1}, Fig. S3).
[e] The major product of this mixture was acyloin **4**, by ^{1}H NMR.
[f] The best yields for the acyloin reaction in deuterated macrocycle were obtained when the reaction was run using mechanical stirring, pre-dispersed sodium in toluene (30%) and a mixture of **3**/**10** and *p*-xylene ($V_{D\text{-}mac}/V_{p\text{-}xylene}$, 1:1).

Further attempts to separate this complex mixture of products by flash column chromatography (SiO_2)[g] and/or size-exclusion chromatography (SX-1, $CHCl_3$) provided **4** (143 mg, 0.28 mmol, 17%), as well as other by-products. Despite detecting the presence of catenane by mass spectrometry, we were unable to isolate catenane directly from chromatography of this mixture, but had to rely on derivatization of the product mixture with a fluorescent tag (which substantially aided the detection and isolation of different products) to separate the catenane from all other species (Section S7).

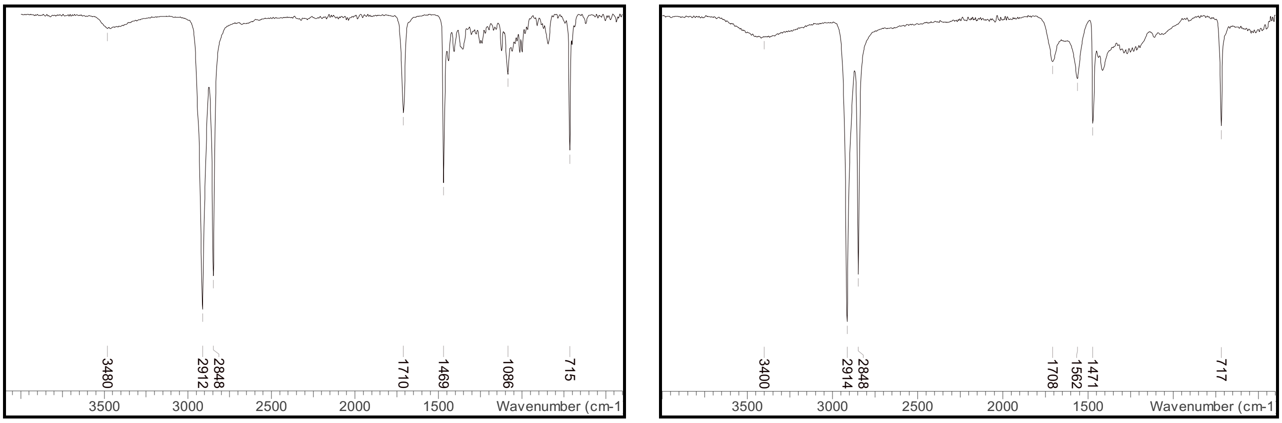

Figure S3: Figure showing the IR spectra of: (left) acyloin **4**; (right) the insoluble solid obtained from the catenane-forming reaction.

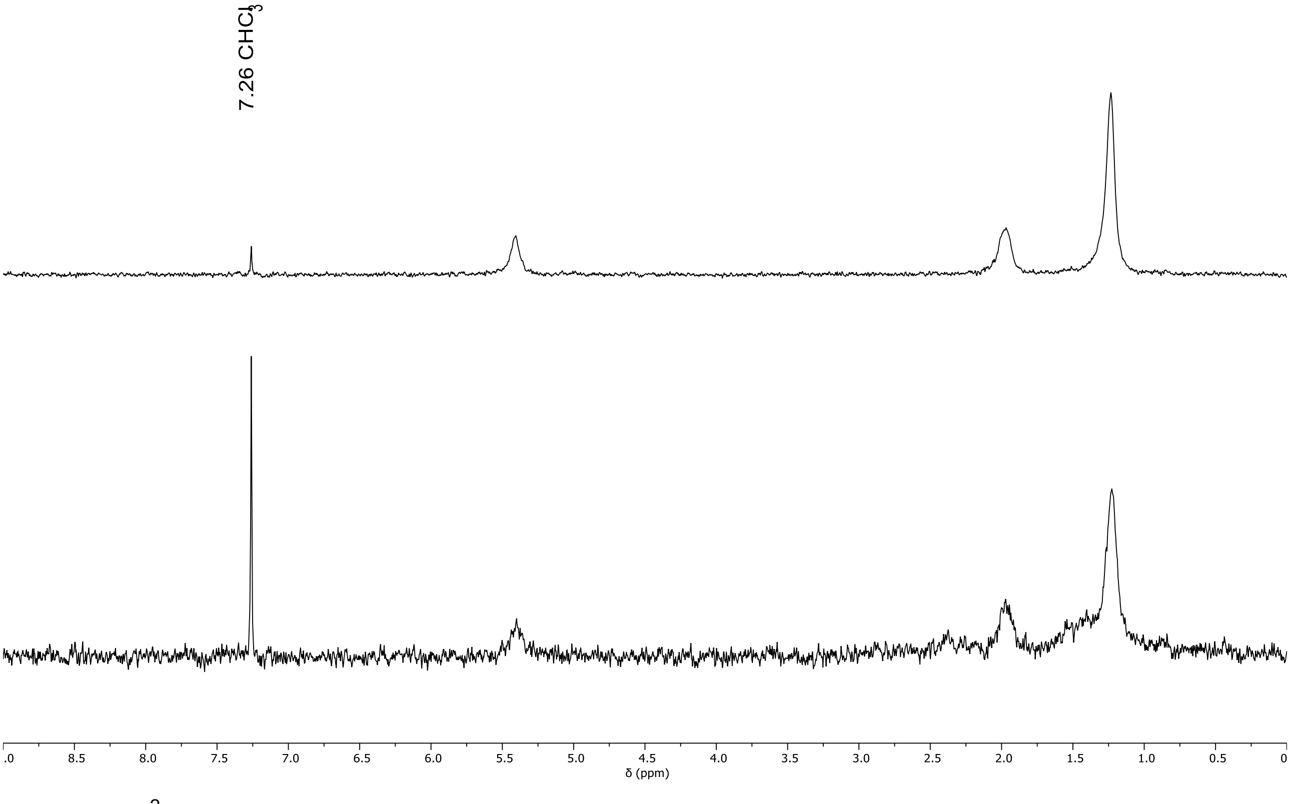

Figure S4. ^{2}H NMR (77 MHz, 298 K, $CHCl_3$) of: (top) Deuterated-macrocycle **3**/**10**; (bottom) the polar mixture obtained from the catenane-forming reaction.

[g] Different conditions were tested, including different polarity eluents, as well as variable polarity elution.

6. Oxidation experiments

6.1. Oxidative cleavage of the polar fraction obtained from the catenane reaction

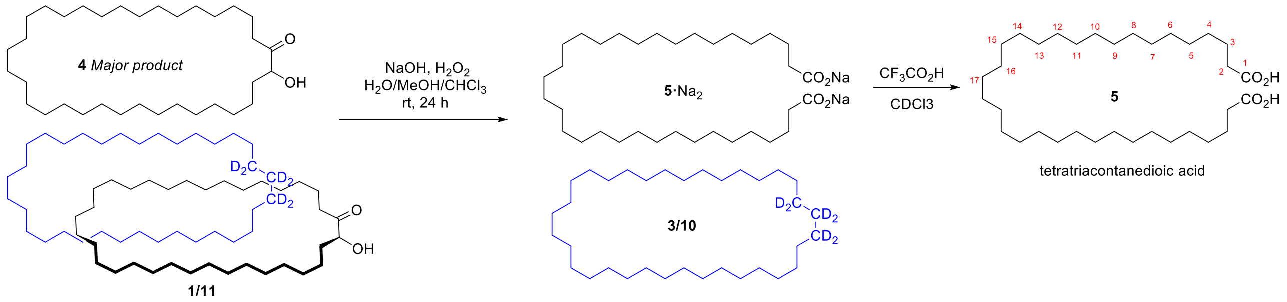

Scheme S8. Oxidation of the polar fraction obtained from the catenane reaction to release **3/10**.

The catenane-forming reaction (Section S5) was repeated[h] and the polar residue obtained (245 mg, assumed 0.48 mmol) was dissolved in a mixture of $CHCl_3$/MeOH (1:1, 10 mL). NaOH (400 mg, 10 mmol) dissolved in H_2O (2 mL) was added, followed by portion-wise addition of 30% H_2O_2 solution (10 mL). The reaction mixture was stirred at room temperature for 24 hours. The precipitated disodium salt of **5**[*] (120 mg, 0.23 mmol, 48%) was filtered off and the solution phase was extracted with $CHCl_3$ (3 × 15 mL). The organic layer was washed with brine, dried over anhydrous $MgSO_4$ and the solvent was removed under reduced pressure to give a yellow greasy solid (36 mg). Analysis by TLC (SiO_2, petroleum ether, Fig. S5) shows the presence of a new apolar species which has an R_F (0.90) near the solvent front. Purification by flash column chromatography (SiO_2, petroleum ether) afforded **3/10** (0.4 mg, 0.07% with respect to the diester starting material). The identity of **3/10** was confirmed by ^{1}H NMR, ^{2}H NMR and HRMS (Fig. S6, a-c).

[*]Full characterization of **5**·Na_2

m.p. sample decomposed at $T > 250$ °C.

^{1}H NMR (600 MHz, 298 K, $CDCl_3/CF_3CO_2H$) δ 10.30 (s, 2H, H_{OH}), 2.44 (t, J = 7.5 Hz, 4H, H_2), 1.66 (p, J = 7.5 Hz, 4H, H_3), 1.26 (s, 56H, $H_{4\text{-}17}$).

^{13}C NMR (151 MHz, , 298 K, $CDCl_3/CF_3CO_2H$) δ 182.8 (C_1), 34.2 (C_2), 29.91, 29.89, 29.87, 29.83, 29.76, 29.6, 29.3 ($C_{5\text{-}17}$), 29.1 (C_4), 24.8 (C_3).

IR (neat **5**·Na_2) v_{max}/cm^{-1}: 2914, 2848, 1697, 1472, 923, 729.

[h] The catenane-forming reaction was repeated before each new experiment and the polar mixture obtained each time was used integrally for that experiment.

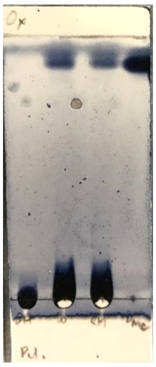

Figure S5. Image of the TLC analysis of the oxidative cleavage (SiO_2, petroleum ether) of the polar fraction obtained from the catenane-forming reaction after staining with CAM. Left-hand lane: starting mixture; middle-left lane: Co-spot of starting mixture/crude reaction mixture; middle-right lane: crude oxidation mixture after 24 hours; right-hand lane: reference **3**/**10**.

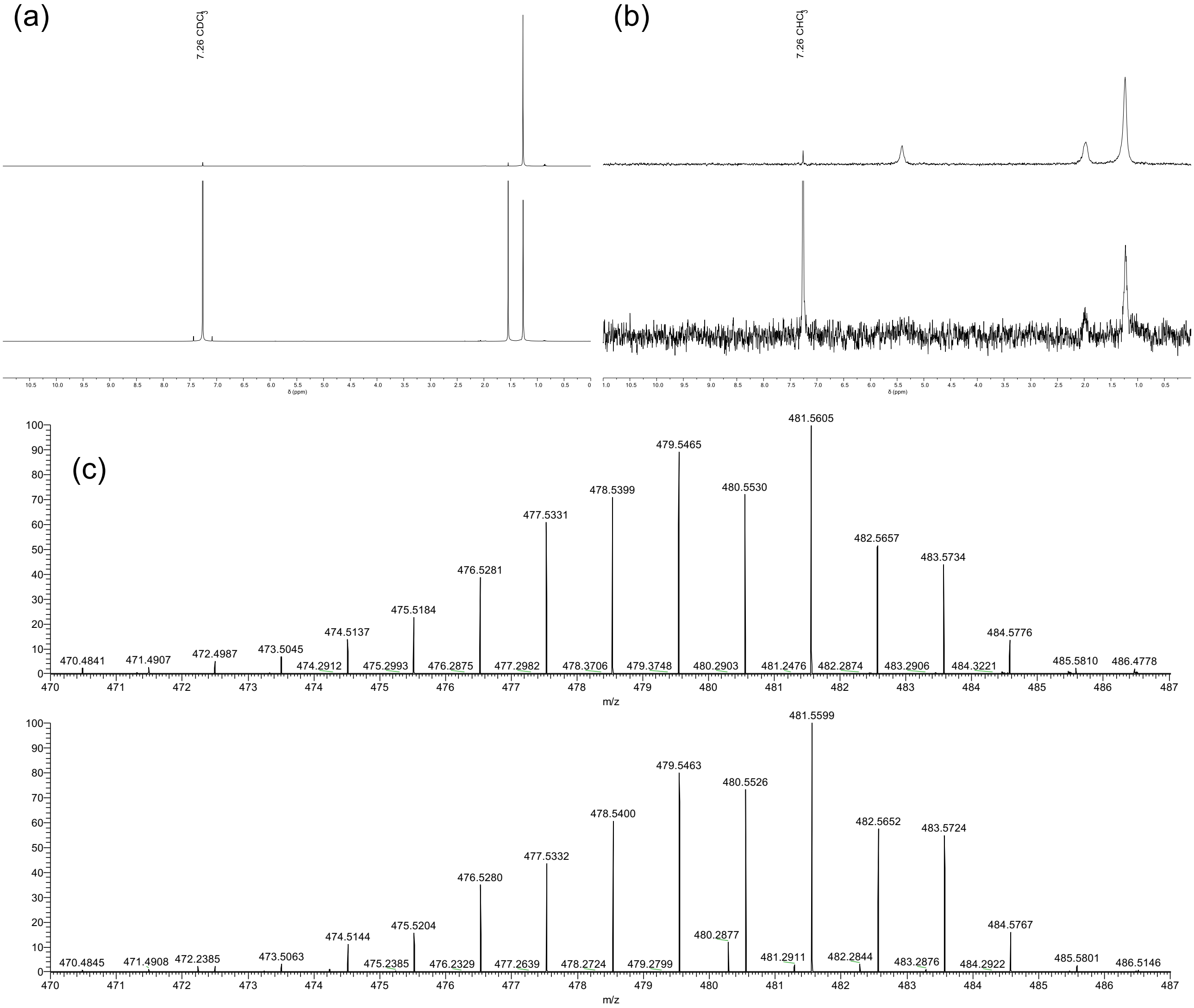

Figure S6. (a) ^{1}H NMR spectra of **3**/**10** isolated after the oxidation of the polar fraction from the catenane-forming reaction (bottom) compared to analytically pure **3**/**10** (top); (b) ^{2}H NMR (77 MHz, $CHCl_3$, 298 K) spectra of **3**/**10** isolated after the oxidation of the polar fraction from the catenane-forming reaction (bottom) compared to analytically pure **3**/**10** (top); (c) HRMS (+APCI) spectra of **3**/**10** isolated after the oxidation of the polar fraction from the catenane-forming reaction (bottom), compared to analytically pure **3**/**10** (top).

6.2. Macrocycle removal control experiment

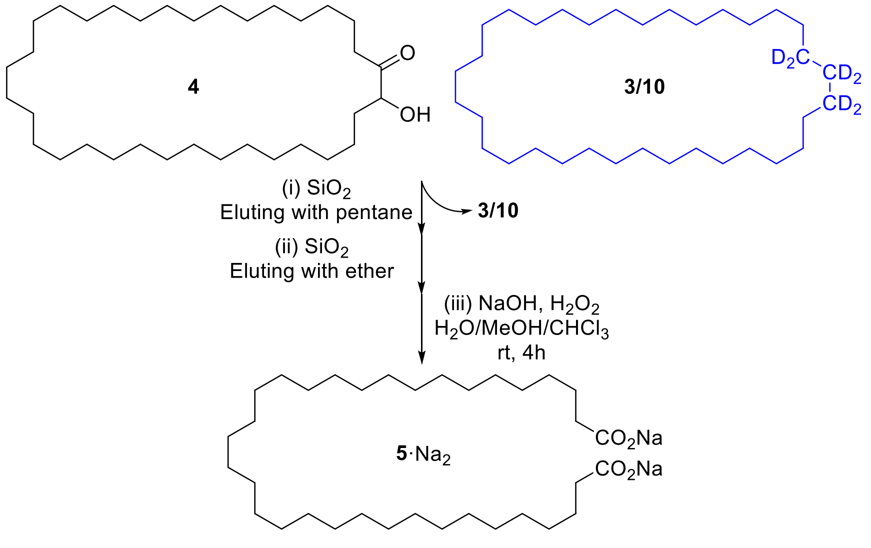

3/10 not observed in crude mixture

Scheme S9. Macrocycle removal control experiment.

4 (200 mg, 0.49 mmol) and **3/10** (7.0 g, 14.5 mmol) were dissolved in $CHCl_3$ (100 mL) and stirred at room temperature for 1 hour. SiO_2 was added and the solvent was removed under reduced pressure. The dry loaded SiO_2 was transferred onto a SiO_2 column and eluted with petroleum ether until all of **3/10** was reisolated (6.9 g). Subsequent elution with petroleum ether/EtOAc (1:1) was used to remove the remaining **4** from the column. Analysis by ^{2}H NMR (Fig. S7, a) indicated that no deuterium containing species were present in the mixture. The polar fraction obtained was dissolved in a mixture of $CHCl_3$/MeOH (1:1, 10 mL), and NaOH (390 mg, 9.8 mmol) in H_2O (2 mL) was added, followed by dropwise addition of 30% H_2O_2 solution (10 mL). The reaction mixture was stirred at room temperature for 24 hours. Analysis by TLC (SiO_2, petroleum ether/EtOAc, 9:1) showed complete consumption of the acyloin and no presence of the deuterated macrocycle in the reaction mixture (Fig. S7, b). The same procedure was used for the oxidation of pure acyloin **4** which was not pre-mixed with deuterated macrocycle (Fig. S7, c) and gave identical results.

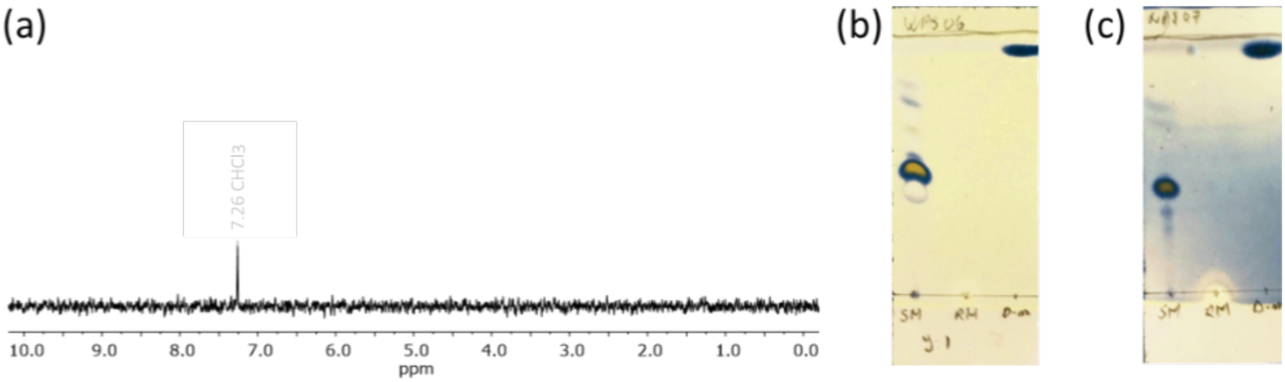

Figure S7. (a) ^{2}H NMR (77 MHz, $CHCl_3$, 298 K) spectrum of the acyloin after removal of excess **3/10**. (b) TLC analysis of the control experiment (SiO_2, petroleum ether/EtOAc, 9:1) after staining with CAM. Left-hand spot: mixture after separation from **3/10**; middle spot: crude oxidation reaction mixture; right-hand spot: reference **3/10**. (c) Reference TLC analysis for the oxidation of acyloin **4** which was not pre-mixed with **3/10** (SiO_2, petroleum ether/EtOAc, 9:1) after staining with CAM. Left-hand spot: acyloin **4**; middle spot: crude oxidation reaction mixture; right-hand spot: reference **3/10**.

7. Tagging experiments

7.1. Tagging of acyloin 4 with 4-nitrobenzoyl chloride

2-oxocyclotetratriacontyl 4-nitrobenzoate (**S3**)

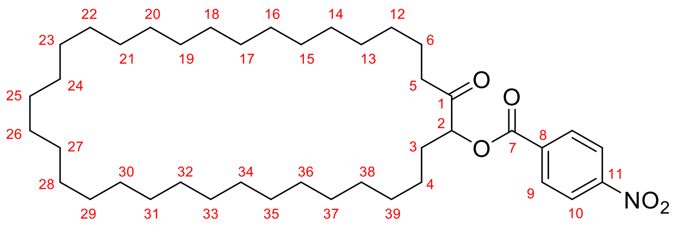

Acyloin **4** (10.0 mg, 19.7 μmol), 4-nitrobenzoyl chloride (5.5 mg, 29.6 μmol) and DMAP (cat.) were dissolved in CH_2Cl_2 (1 mL). DIPEA (7 μL, 5.2 mg, 40.2 μmol) was added and the reaction stirred at room temperature for 2 hours. The reaction crude was partitioned between CH_2Cl_2 (2 × 10 mL) and a saturated aqueous solution of ammonium chloride (10 mL). The organic layer was separated and dried over anhydrous $MgSO_4$ and the solvent was removed under reduced pressure. The residue was purified by flash column chromatography (SiO_2, petroleum ether/EtOAc, 30:1) to give compound **S3** (9.2 mg, 14.0 μmol, 71 %) as a colorless solid.

m.p. 68 – 70 °C.

^{1}H NMR (600 MHz, 298 K, $CDCl_3$) δ 8.31 (d, J = 8.8 Hz, 2H, H_{10}), 8.24 (d, J = 8.8 Hz, 2H, H_9), 5.29 (dd, J = 7.1, 5.1 Hz, 1H, H_2), 2.57 (dt, J = 17.2, 7.2 Hz, 1H, H_5), 2.48 (dt, J = 17.2, 7.4 Hz, 1H, $H_{5'}$), 1.93 (q, J = 7.6, Hz, 2H, H_3), 1.64 (p, J = 7.2 Hz, 2H, H_6), 1.53 – 1.43 (m, 2H, H_4), 1.43 – 1.27 (m, 55H, $H_{12\text{-}39}$).

^{13}C NMR (151 MHz, 298 K, $CDCl_3$) δ 206.5 (C_1), 164.4 (C_7), 150.9 (C_8), 135.2 (C_{11}), 131.1 (C_9), 123.8 (C_{10}), 79.8 (C_2), 38.9 (C_5), 30.5 (C_3), 29.54, 29.51, 29.48, 29.46, 29.41, 29.37, 29.30, 29.28, 29.24, 29.22, 29.20, 29.18, 29.17, 29.14, 25.2 ($C_{12\text{-}39}$), 23.2 (C_6).

HRMS (+APCI) m/z: calcd. for $C_{41}H_{70}NO_5$ $[M+H]^+$ 656.5249, found 656.5245

IR (neat) v_{max}/cm^{-1}: 2916, 2846, 1525, 1464, 1342, 716.

7.2. Attempt to form the original Wasserman catenane (1) with subsequent tagging of the crude product mixture with 4-nitrobenzoyl chloride

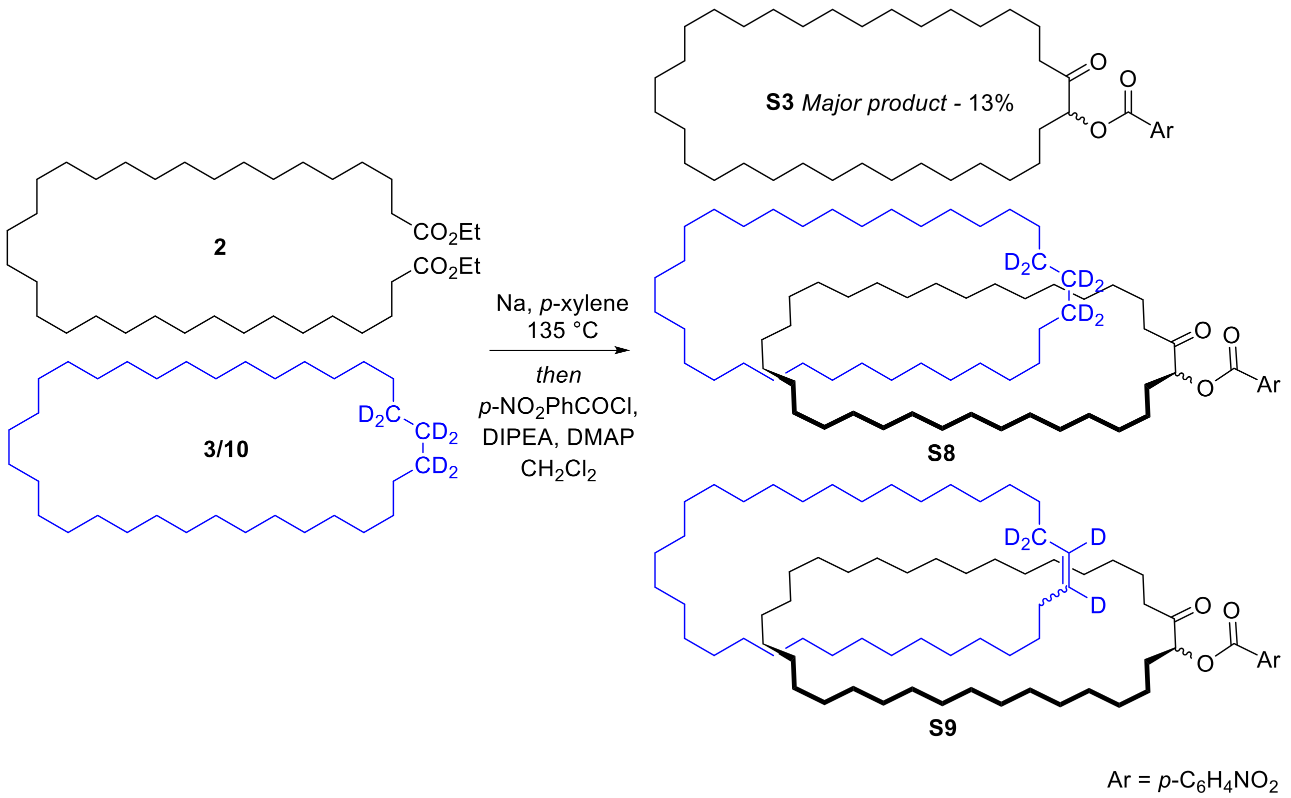

Scheme S10. Acyloin condensation in the presence of a large excess of deuterated cyclotetratriacontane/ene and subsequent tagging of the crude product mixture with 4-nitrobenzoyl chloride.

In a flame-dried flask, finely cut sodium pieces (1.0 g, 43.5 mmol) were added to a mixture of dry *p*-xylene (10 mL) and cyclotetratriacontane/ene **3**/**10** (10.0 g, 20.8 mmol). The mixture was brought to reflux under vigorous mechanical stirring to promote the formation of a sodium dispersion. To the dispersion, diester **2** (1.0 g, 1.68 mmol) was added and the reaction mixture was stirred at 135 °C overnight. After 20 hours, the mixture was cooled to room temperature and quenched with MeOH (20 mL) and then aq. 1M HCl (10 mL). The product was extracted with $CHCl_3$ (3 × 200 mL) and petroleum ether (3 × 200 mL), the combined organic phases were dried over anhydrous $MgSO_4$ and the solvent was removed under reduced pressure. The crude obtained was purified by flash column chromatography (SiO_2, petroleum ether, then petroleum ether/EtOAc, 1:1). to yield **3**/**10** (9.9 g) and a mixture of polar compounds (900 mg), respectively. This mixture was redissolved in $CHCl_3$ (10 mL), to which 4-nitrobenzoyl chloride (500 mg, 2.69 mmol), DMAP (22 mg, 0.18 mmol) and DIPEA (0.62 mL, 460 mg, 3.56 mmol) were added, and the reaction was stirred at room temperature overnight. CH_2Cl_2 (250 mL) and sat. aq. NH_4Cl (250 mL) were added, and the organic phase was separated and dried over anhydrous $MgSO_4$. The solvent was removed under reduced pressure and the crude obtained was purified by flash column chromatography (SiO_2, petroleum ether/EtOAc, 30:1) to give **S3** (140 mg, 0.21 mmol, 13%), as a colorless solid.

A second fraction was isolated from the column chromatography as a mixture of different compounds (1 mg, < 1%). Analysis by 1D/2D $^{1}H/^{13}C$ NMR (see Fig. S9 and Section 9, Spectra S21-S25) indicated the presence of at least four compounds, the structures of the major species are shown in Fig. S8.

Compounds 1-4 - Minor products - Isolated as an inseparable mixture (~1 mg)

Compound 1

a
^{13}C 212.1 (C) ppm
b
^{1}H 2.39 (t, J = 7.3 Hz) ppm
^{13}C 42.8 (CH_2) ppm
c
^{1}H 1.60 (m) ppm
^{13}C 24.0 (CH_2) ppm

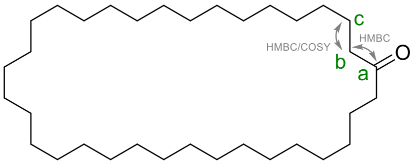

Ketone **S6**

Compound 2

a
^{1}H 5.18 (p) ppm
^{13}C 76.3 (CH) ppm
b
^{1}H 1.71 (m) ppm
^{13}C 33.7 (CH_2) ppm
c
^{13}C 164.4 (C) ppm

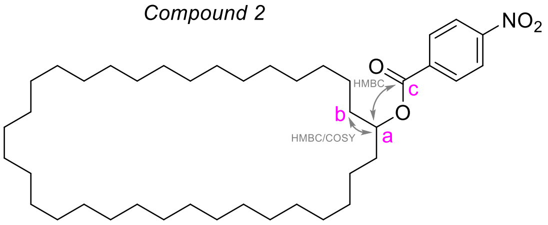

Cyclic alcohol **S10a**

Compound 3

a
^{1}H 4.39 (t, J = 6.7 Hz) ppm
^{13}C 66.1 (CH) ppm
b
^{1}H 1.81 (p, J = 6.8 Hz) ppm
^{13}C 28.6 (CH_2) ppm
c
^{13}C 164.7 (C) ppm

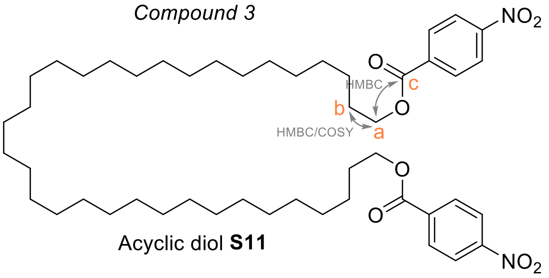

Acyclic diol **S11**

Compound 4

a
^{1}H 4.29 (d, J = 5.8 Hz) ppm
^{13}C 69.0 (CH_2) ppm
b
^{1}H 1.84 (m) ppm
^{13}C 37.1 (CH) ppm
c
^{13}C 164.8 (C) ppm

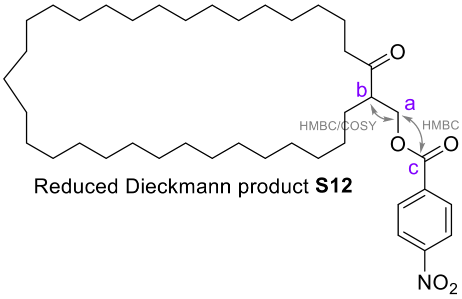

Reduced Dieckmann product **S12**

Figure S8. Product mixture isolated from acyloin condensation of diester **2**.

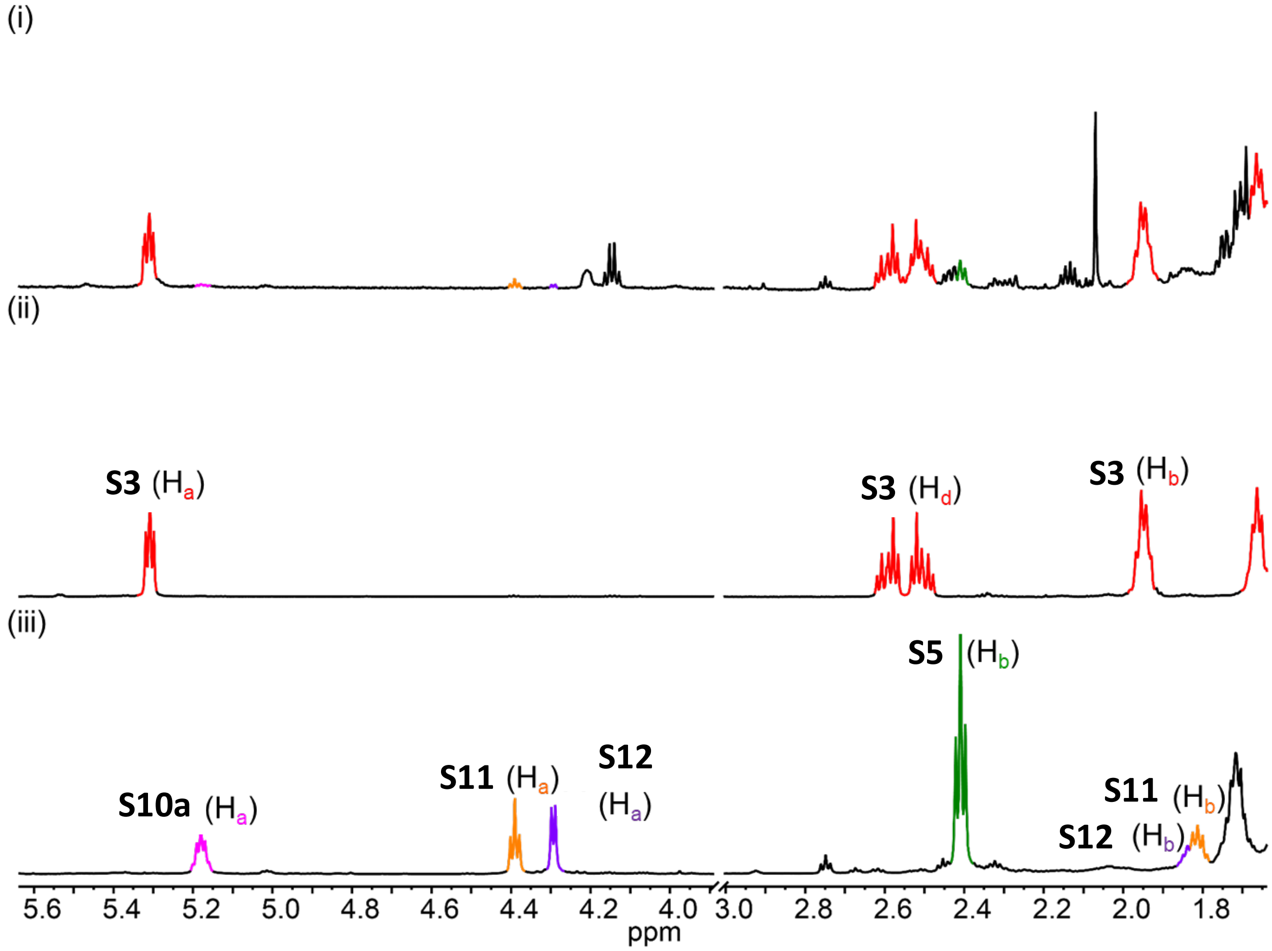

Figure S9. Partial ^{1}H NMR spectra (600 MHz, 298 K, $CDCl_3$) of (i) product mixture obtained from the tagging reaction; (ii) isolated tagged-acyloin **S3**; (iii) mixture of minor products **S5**, **S10a**, **S11** and **S12**.

7.3. Tagging of acyloin 4 with dansyl chloride

2-oxocyclotetratriacontyl 5-(dimethylamino)naphthalene-2-sulfonate (**S4**)

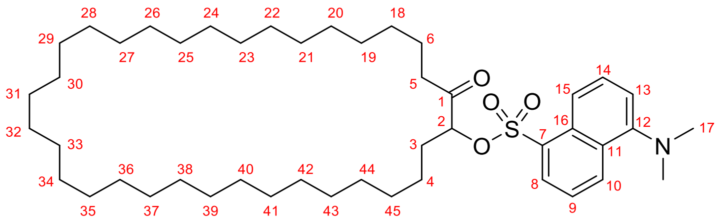

Acyloin **4** (20 mg, 39.0 µmol), dansyl chloride (65 mg, 237 µmol) and DMAP (23 mg, 195 µmol) were dissolved in $CHCl_3$ (1.2 mL). DIPEA (60 µL, 50 mg, 390 µmol) was added and the reaction was stirred at room temperature for 1.5 hours. The solvent was removed under reduced pressure and the residue obtained was purified by flash chromatography (SiO_2, CH_2Cl_2) to give **S4** (24 mg, 32 µmol, 82 %) as a light-yellow solid.

m.p. 62 – 64 °C.

^{1}H NMR (600 MHz, 298 K, $CDCl_3$δ 8.61 (d, *J* = 8.5 Hz, 1H, H_{10}), 8.33 (d, *J* = 8.6 Hz, 1H, H_{15}), 8.23 (d, *J* = 7.3, 1H, H_8), 7.61 (t, *J* = 8.1 Hz, 1H, H_{14}), 7.54 (t, *J* = 8.1 Hz, 1H, H_9), 7.21 (d, *J* = 7.6 Hz, 1H, H_{13}), 4.57 (dd, *J* = 7.3, 4.9 Hz, 1H, H_2), 2.89 (s, 6H, H_{17}), 2.61 – 2.44 (m, 2H, H_5), 1.69 – 1.62 (m, 1H, $H_{3'}$), 1.54 – 1.39 (m, 3H, $H_{3'', 6}$), 1.26 (s, 56H, $H_{18\text{-}45}$), 1.06 (p, *J* = 7.3 Hz, 2H, H_4).

^{13}C NMR (151 MHz, 298 K, $CDCl_3$) δ 207.7 (C_1), 152.0 (C_{12}), 132.0 (C_{10}), 131.5 (C_{11}), 130.6 (C_8), 130.02 (C_7), 129.98 (C_{16}), 128.9 (C_{14}), 123.2 (C_9), 119.6 (C_{15}), 115.8 (C_{13}), 85.2 (C_2), 45.6 (C_{17}), 38.4 (C_5), 31.7 (C_3), 29.5, 29.4, 29.3, 29.2, 28.9 ($C_{18\text{-}45}$), 28.7, 24.1 (C_4), 22.7 (C_6).

HRMS (+APCI) m/z: calcd. for $C_{46}H_{78}NO_4S$ $[M+H]^+$ 740.5625, found 740.5646.

IR (neat) v_{max}/cm^{-1}: 2912, 2848, 1715, 1576, 1470, 1408, 1362, 1174, 787.

7.4. Isolation of 12a/12b aided by labelling with dansyl chloride

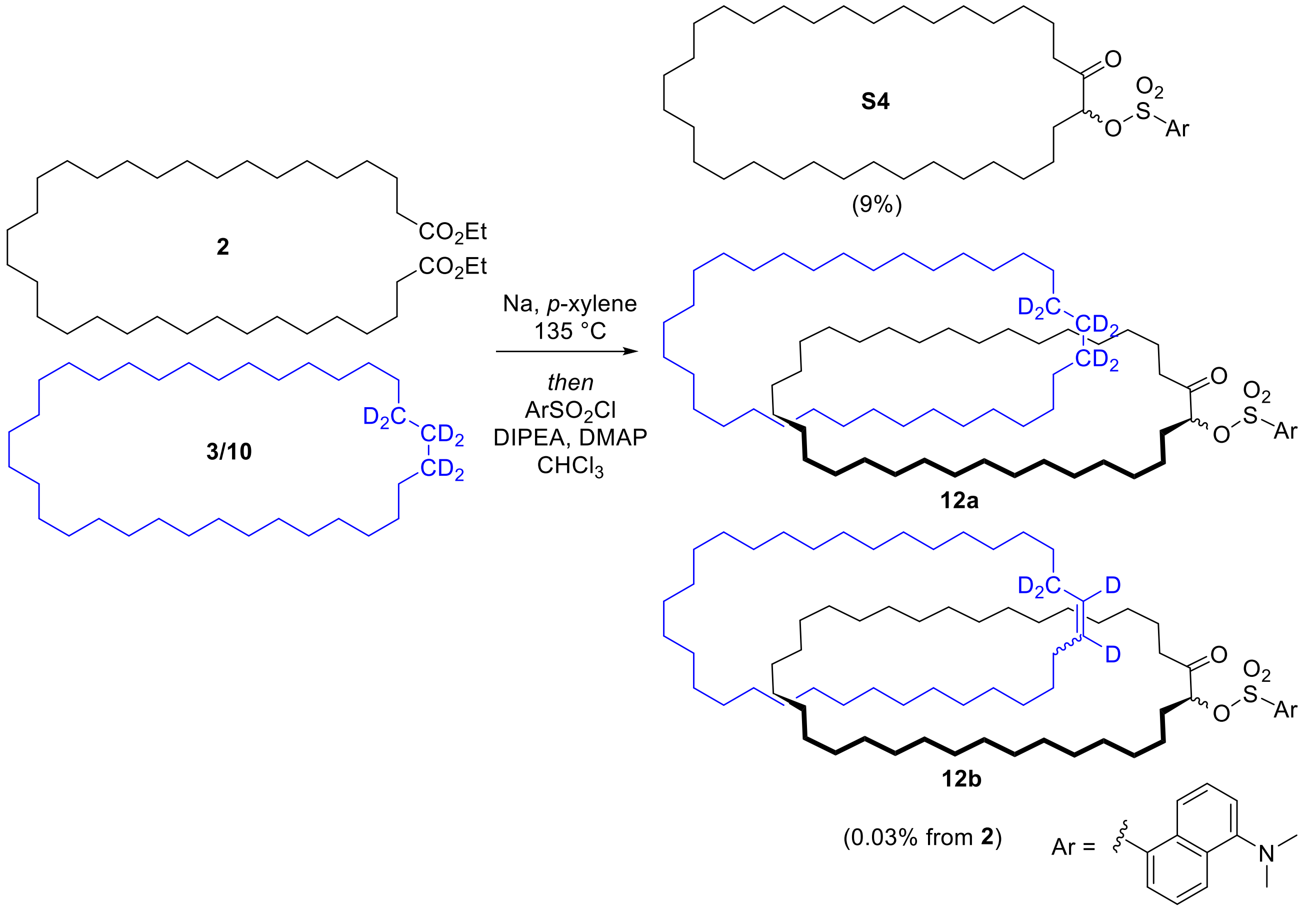

Scheme S11. Acyloin condensation in the presence of a large excess of deuterated species and subsequent dansyl-tagging of the polar mixture.

The polar mixture (198 mg, assumed 0.39 mmol) obtained from a catenane-forming reaction (See Section S5) was dissolved in $CHCl_3$ (10 mL). To this, dansyl chloride (526 mg, 1.95 mmol), DMAP (238 mg, 1.95 mmol) and DIPEA (0.68 mL, 3.90 mmol) were added. The reaction was stirred at room temperature for 1.5 hours. $CHCl_3$ (50 mL) was then added, and the solution was washed with sat. aq. NH_4Cl, sat. aq. $NaHCO_3$, brine and dried over $MgSO_4$. The solvent was removed under reduced pressure to give a crude residue. This was purified by size-exclusion chromatography (SX-1, $CHCl_3$) to separate **S4**[i] (114 mg, 0.15 mmol, 9% from **2**) as the major product, as well as small amounts of other low-molecular weight macrocyclic byproducts (Fig. S10). A different fraction, containing larger molecules, was further purified by flash column chromatography (SiO_2, CH_2Cl_2) to yield the dansylated catenanes **12a**/**12b** (0.7 mg, 0.57 μmol, 0.03% from **2**) and acyloin macrocyclic dimer derivatives (Fig. S11).

[i] Analytical data matches that obtained in Section 7.3.

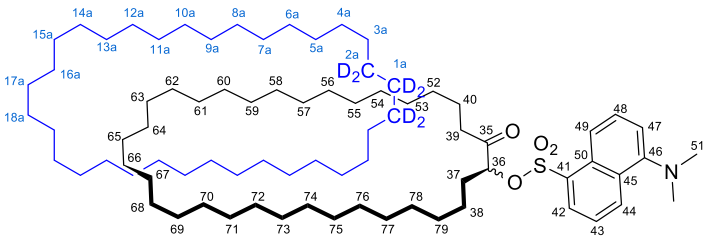

12a

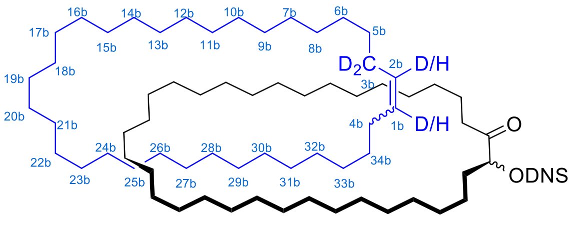

12b

^{1}H NMR (600 MHz, 298 K, $CDCl_3$) δ 8.61 (dt, J = 8.5, 1.1 Hz, 1H, H_{44}), 8.33 (dt, J = 8.6, 1.0 Hz, 1H, H_{49}), 8.23 (dd, J = 7.3, 1.1 Hz, 1H, H_{42}), 7.60 (dd, J = 8.6, 7.6 Hz, 1H, H_{48}), 7.53 (dd, J = 8.5, 7.3 Hz, 1H, H_{43}), 7.20 (dd, J = 7.6, 1.0 Hz, 1H, H_{47}), 5.39 – 5.31 (m, $H_{1b,2b}$), 4.55 (dd, J = 7.5, 4.8 Hz, 1H, H_{36}), 2.89 (s, 6H, H_{51}), 2.57 – 2.43 (m, 2H), 2.03 – 1.93 (m, H_{4b}), 1.68 – 1.61 (m, 1H, $H_{37'}$), 1.52 – 1.35 (m, 3H, $H_{37'',40}$), 1.26 (m, 118H, $H_{3a-18a,5b-34b,52-79}$), 1.08 (m, J = 7.1 Hz, 2H, H_{38}).

^{13}C NMR (151 MHz, 298 K, $CDCl_3$) δ 207.3 (C_{35}), 152.0 (C_{46}), 132.0 (C_{44}), 131.5 (C_{45}), 130.7 (C_{42}), 130.3 ($C_{1b/2b}$), 130.0 (C_{41}), 129.9 ($C_{2b/1b}$), 128.9 (C_{48}), 123.2 (C_{43}), 119.6 (C_{49}), 115.7 (C_{47}), 85.2 (C_{36}), 45.6 (C_{51}), 38.5, 32.7 – 32.6 ($C_{3b/4b}$), 31.6 (C_{37}), 30.0, 29.8, 29.7, 29.6, 29.5, 29.2, 29.1, 29.0 ($C_{1a-18a,5b-34b,52-79}$), 27.4 – 27.3 ($C_{4b/2b}$), 24.3 (C_{38}), 22.9 (C_{40}).

^{2}H NMR (77 MHz, 298 K, $CHCl_3$) δ 5.40 (br s, $D_{1b,2b}$), 1.97 (br s, D_{3b}), 1.23 (br s, $D_{1a,2a}$).

HRMS (+APCI) m/z: calcd. for $C_{80}H_{140}D_6NO_4S$ $[M+H]^+$ 1223.1344, found 1223.1335 (**12a**); calcd. for $C_{80}H_{140}D_4NO_4S$ $[M+H]^+$ 1219.1062, found 1219.1047 (**12b**). (see Section 8.4, Fig. S21–S24 for full spectrum).

a
^{1}H 4.50 (p, J = 5.6 Hz) ppm
^{13}C 85.2 (CH) ppm
b
^{1}H 1.48 (m) ppm
^{13}C 33.7 (CH_2) ppm
c
^{1}H 1.10 (m) ppm
^{13}C 24.4 (CH_2) ppm

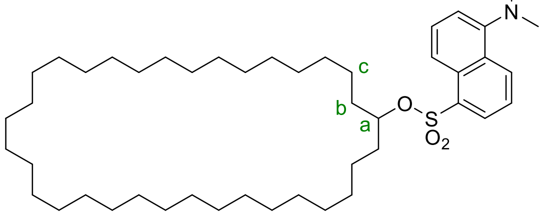

Cyclic alcohol **S10b**

HRMS

calcd. for $C_{47}H_{79}NO_3SH$ $[M+H]^+$
726.5853, found 726.5854

a
^{1}H 4.11 (dt, J = 10.2, 3.4 Hz) ppm
^{13}C 81.7 (CH) ppm
b
^{1}H 1.37 (m) ppm
^{13}C 28.1 (CH_2) ppm
c
^{1}H 0.82 (m) ppm
^{13}C 24.8 (C) ppm

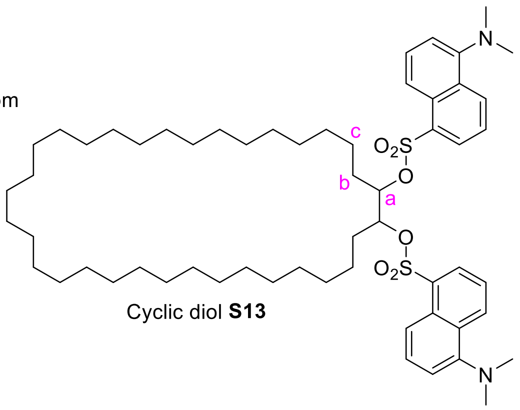

Cyclic diol **S13**

calcd. for $C_{58}H_{90}N_2O_6S_2H$ $[M+H]^+$
975.6313, found 975.6315

Figure S10: Small-molecular weight compounds isolated from size-exclusion chromatography following the dansylation of a polar mixture from the catenane-forming reaction.

a

^{1}H 4.57 (t, *J* = 5.9 Hz) ppm

^{13}C 85.0 (CH) ppm

HRMS: calcd. for $C_{92}H_{155}N_2O_8S_2$ [M+H]$^+$ 1480.1219, found 1480.1233

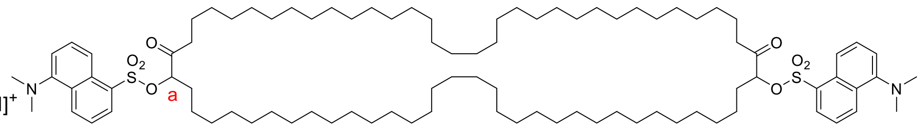

Didansylated diacyloin **S14**

a

^{1}H 4.55 (t, *J* = 6.3 Hz) ppm

^{13}C 85.2 (CH) ppm

b

^{1}H 4.18 (dt, *J* = 9.9, 5.4 Hz) ppm

^{13}C 63.9 (CH) ppm

HRMS: calcd. for $C_{80}H_{143}NO_6SNa$ [M+Na]$^+$ 1269.0534, found 1269.0528

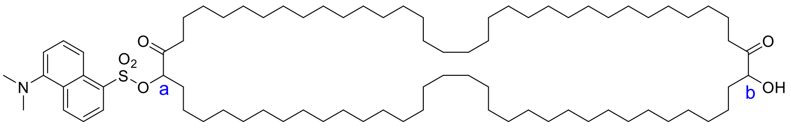

Monodansylated diacyloin **S15**

Figure S11: Large-molecular weight compounds isolated from size-exclusion chromatography and subsequent flash column chromatography following the dansylation of a polar mixture from the catenane-forming reaction.

8. MS spectra

8.1. HRMS (APCI$^+$) spectra of deuterated macrocycle 3/10

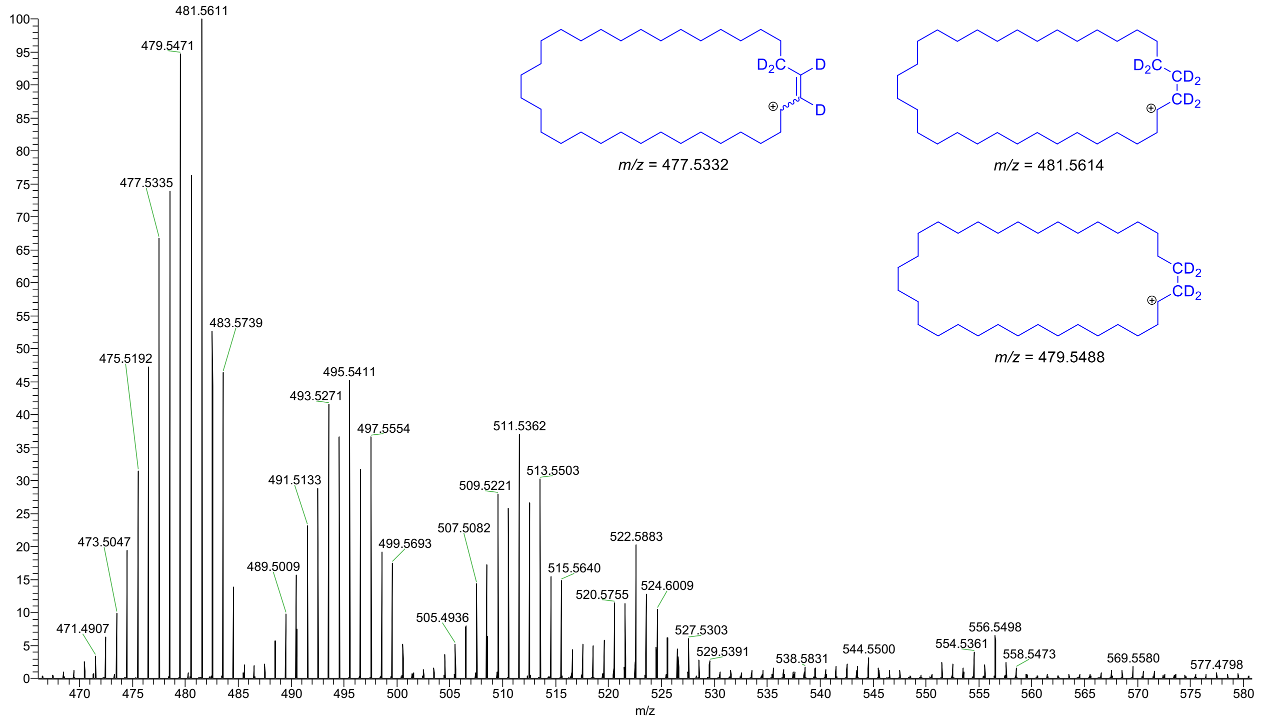

Figure S12: HRMS (+APCI) spectrum of **3**/**10**.

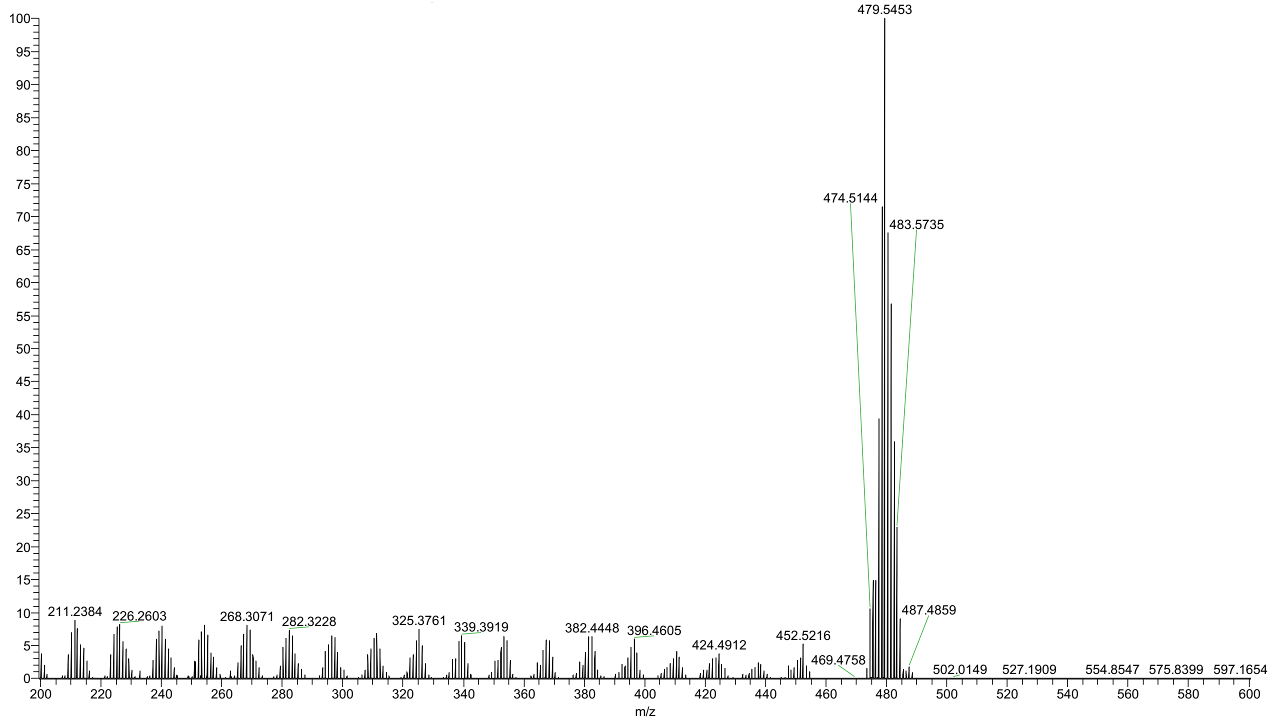

Figure S13: MS/MS (+APCI) spectrum of **3**/**10**, using 20 eV collision energy to fragment the parent ions (*m/z* = 473 to 481, [**3**/**10**-H]$^+$).

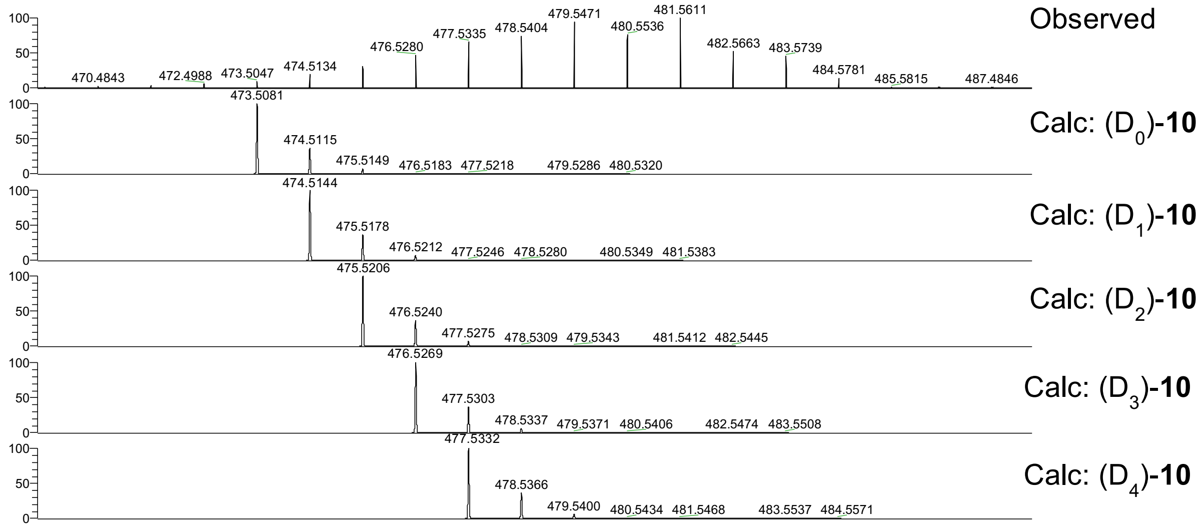

Figure S14: Stack-plot showing the observed HRMS (+APCI) spectrum of deuterated **3**/**10** (top) and the predicted spectra for the alkene species **10** ($C_{34}H_{65-x}D_x$, $_{x=0–4}$ $[M\text{-}H]^+$).

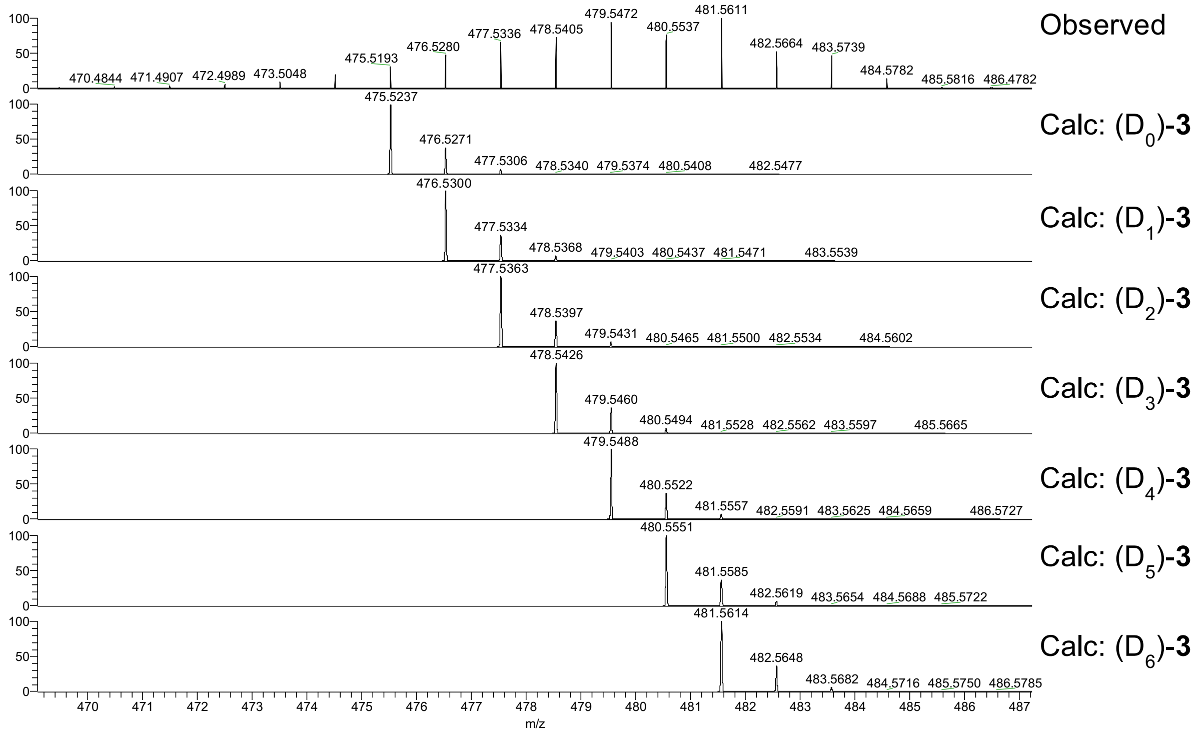

Figure S15: Stack-plot showing the observed HRMS (+APCI) spectrum of deuterated **3**/**10** (top) and the predicted spectra for the alkane species **3** ($C_{34}H_{67-x}D_x$, $_{x=0–6}$ $[M\text{-}H]^+$).

8.2. HRMS (APCI$^+$) spectrum of acyloin 4

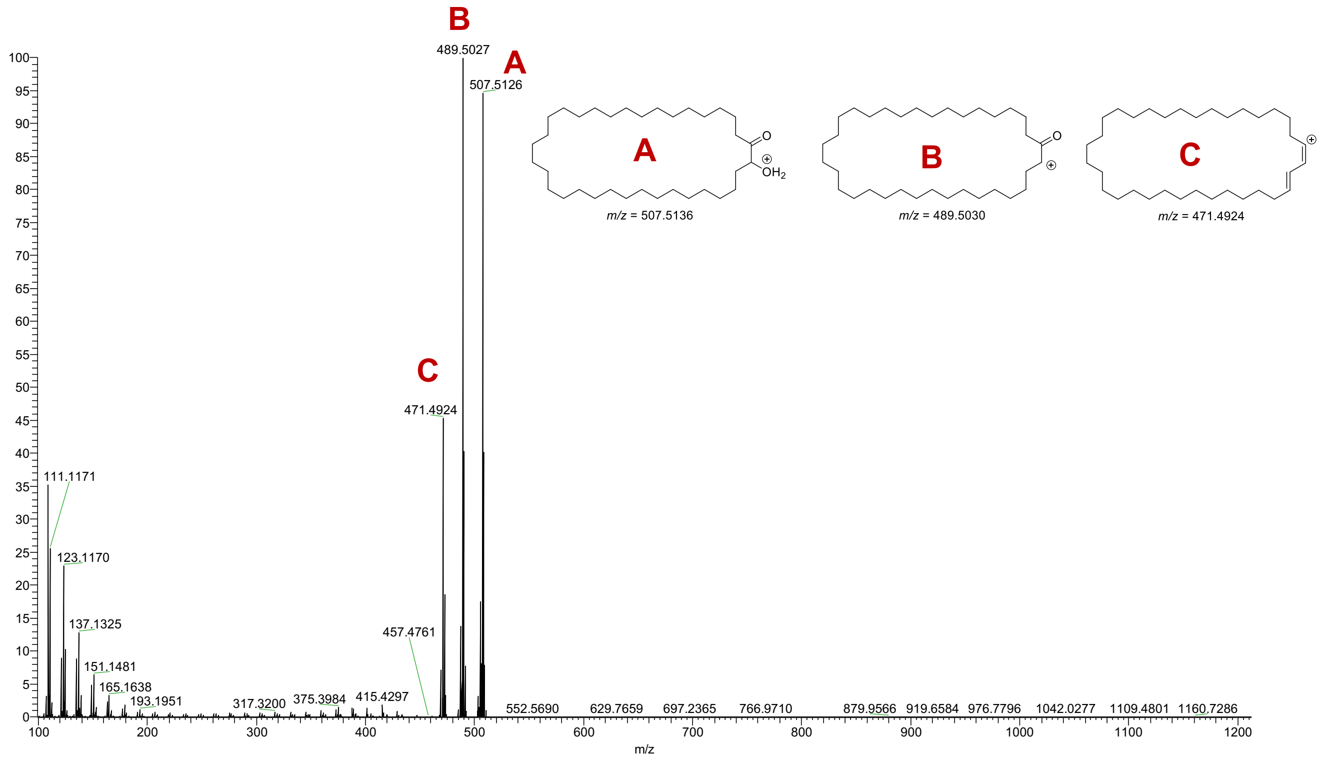

Figure S16: MS/MS (+APCI) spectrum of acyloin **4**, using 20 eV collision energy to fragment the parent ion (*m/z* = 507, [**4**+H]$^+$).

8.3. HRMS (APCI$^+$) spectra of the mixture of polar products isolated from the catenane-forming reaction

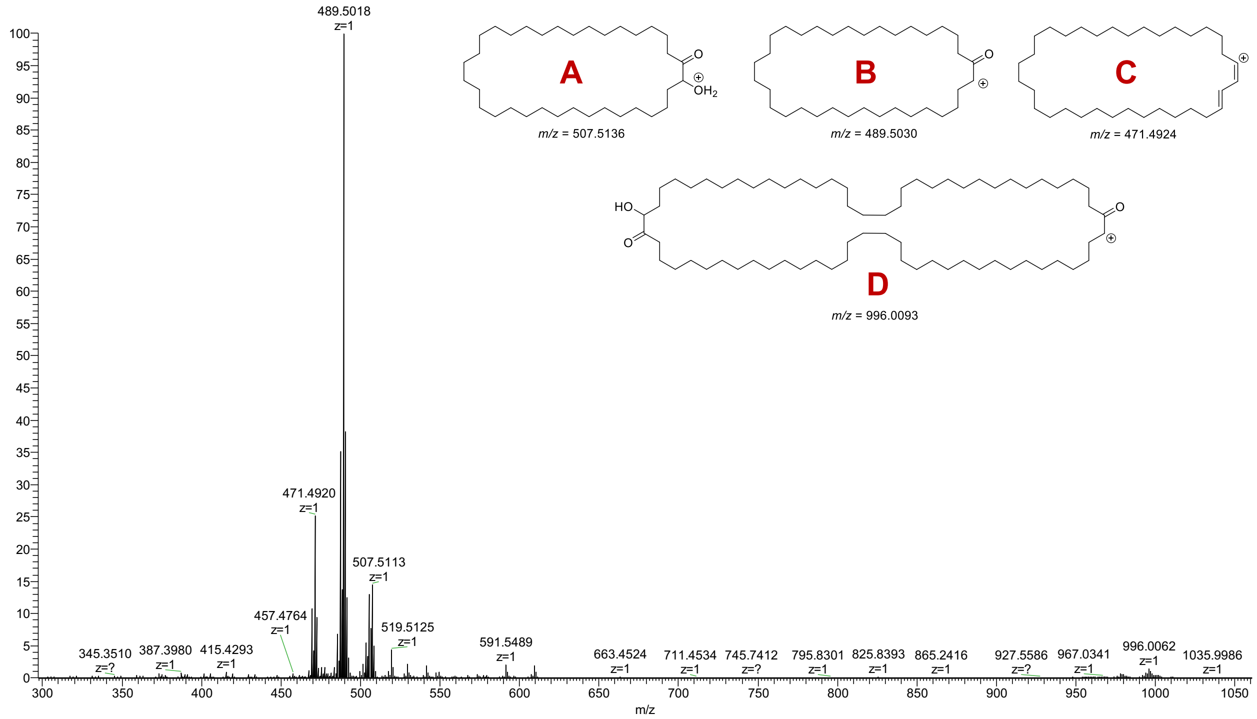

Figure S17: HRMS (+APCI) spectrum of the polar mixture obtained from the catenane-forming reaction.

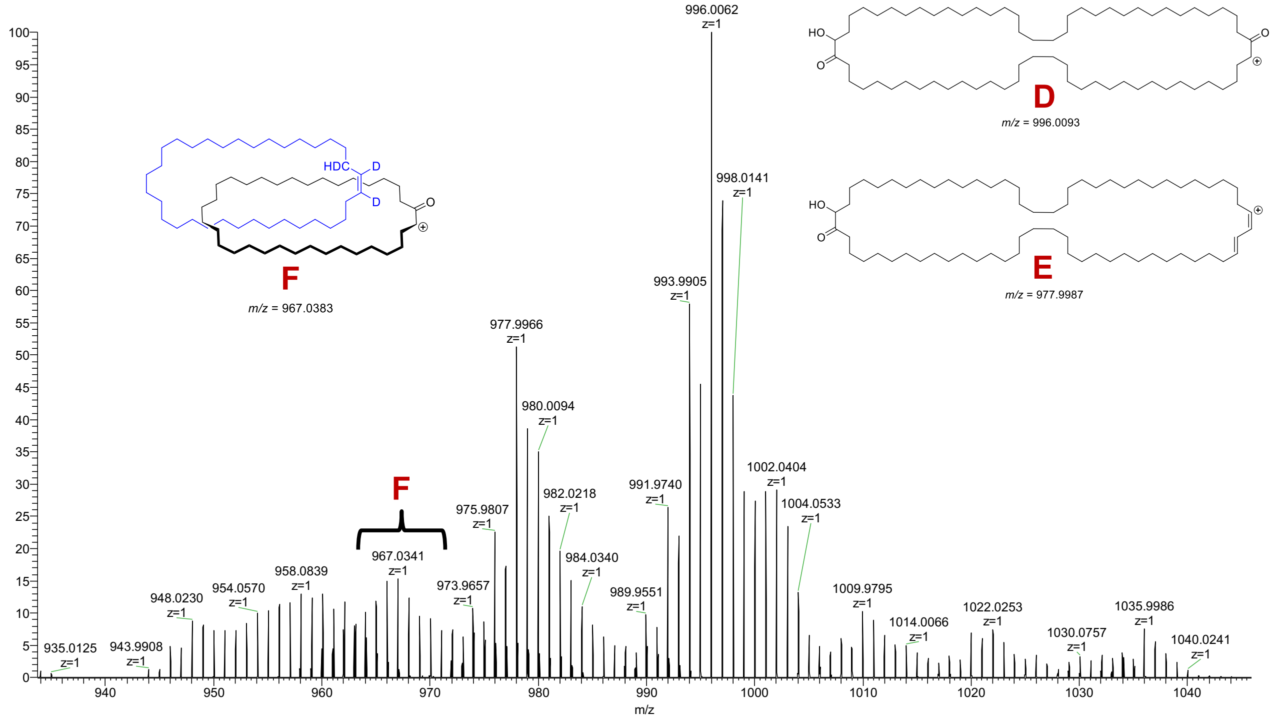

Figure S18: HRMS (+APCI) spectrum of the polar mixture obtained from the catenane-forming reaction, zoom-in on the catenane fragment peaks (*m/z* = 964 to 972).

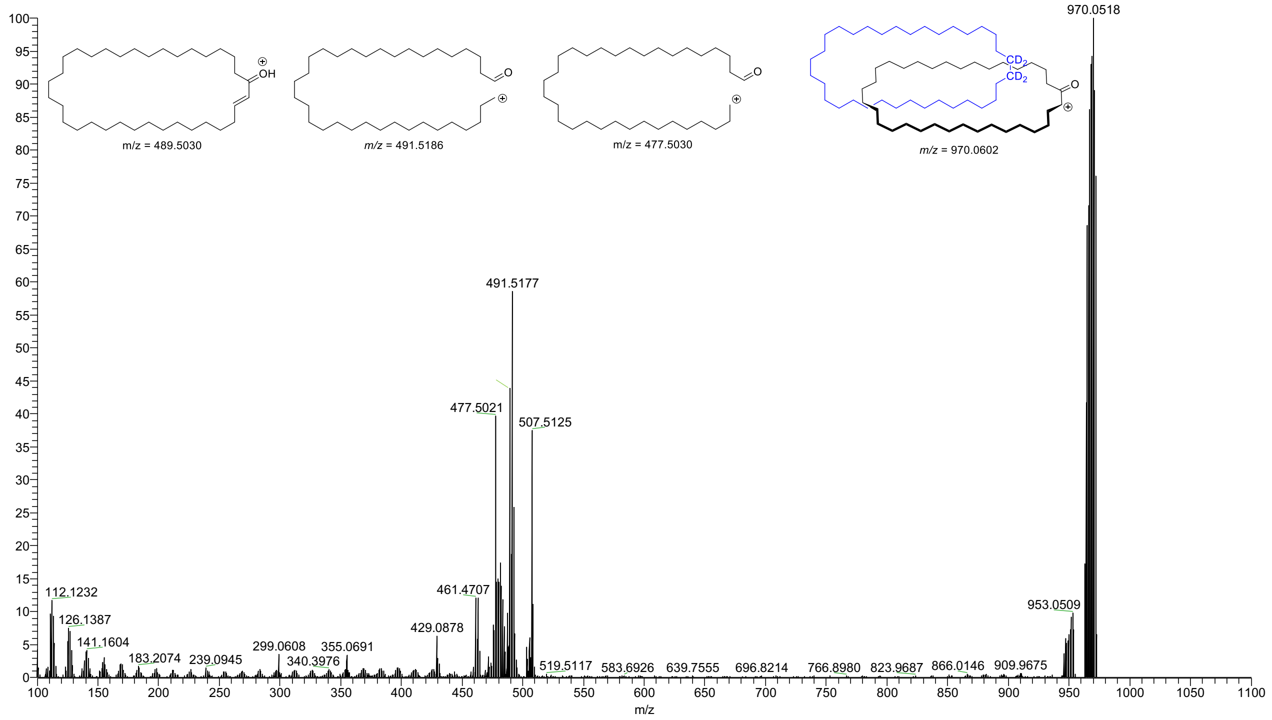

Figure S19: MS/MS (+APCI) analysis of the catenane fragment peaks (*m/z* = 968 ± 5 Da, [**1**/**11**-H_2O]H^+), using 20 eV collision energy for fragmentation.

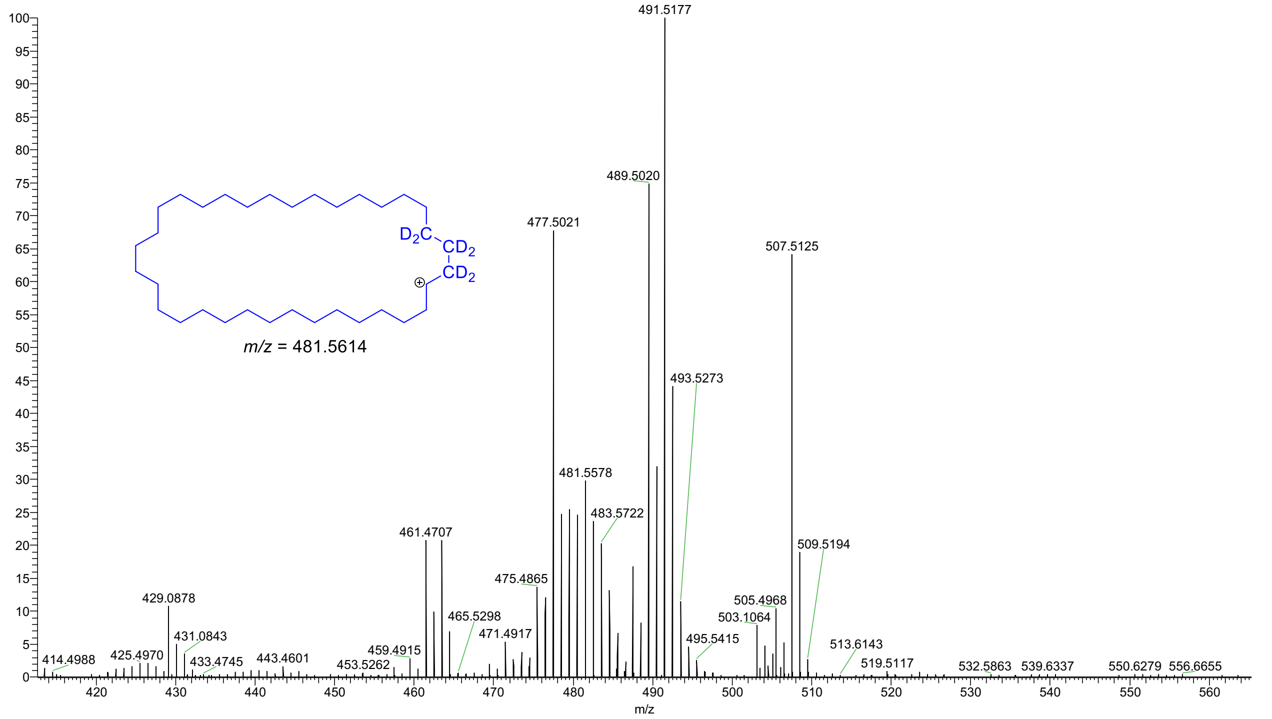

Figure S20: MS/MS (+APCI) analysis of the catenane fragment peaks (*m/z* = 968 ± 5 Da), zoom-in on the fragmented peaks.

8.4. HRMS (APCI$^+$) spectra of the isolated dansyl-catenane mixture 12a/12b

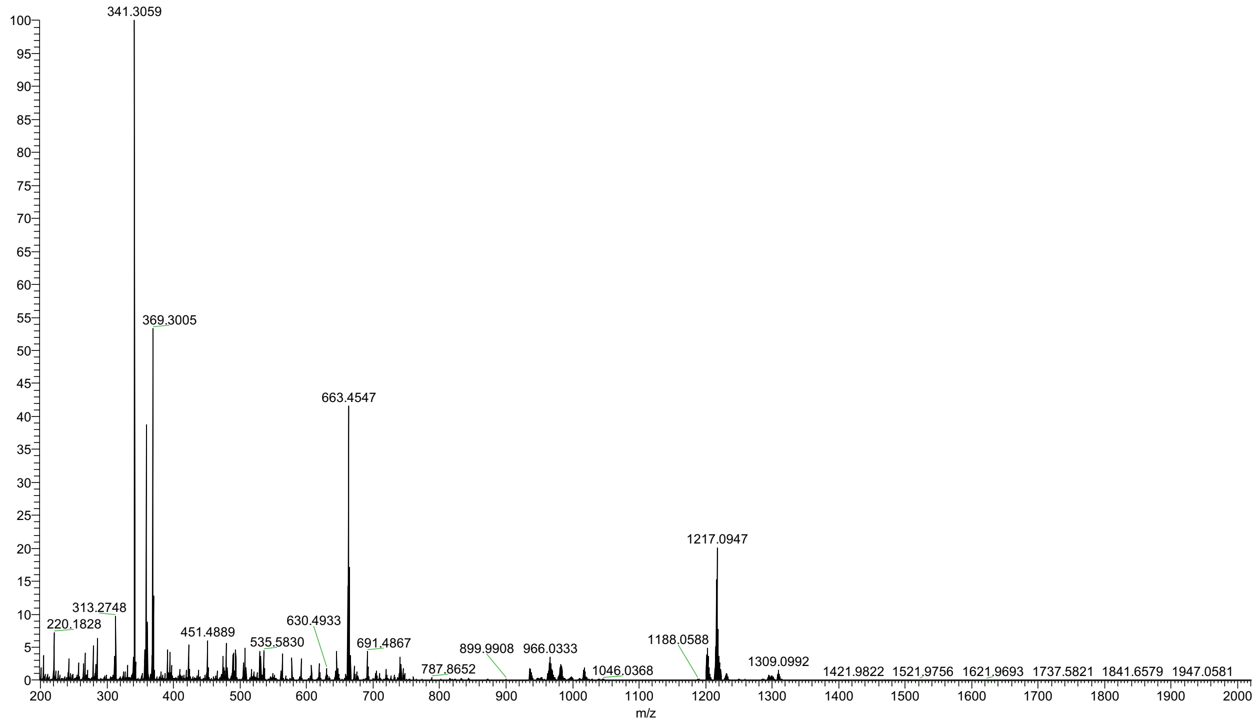

Figure S21: HRMS (+APCI) analysis of **12a/12b**

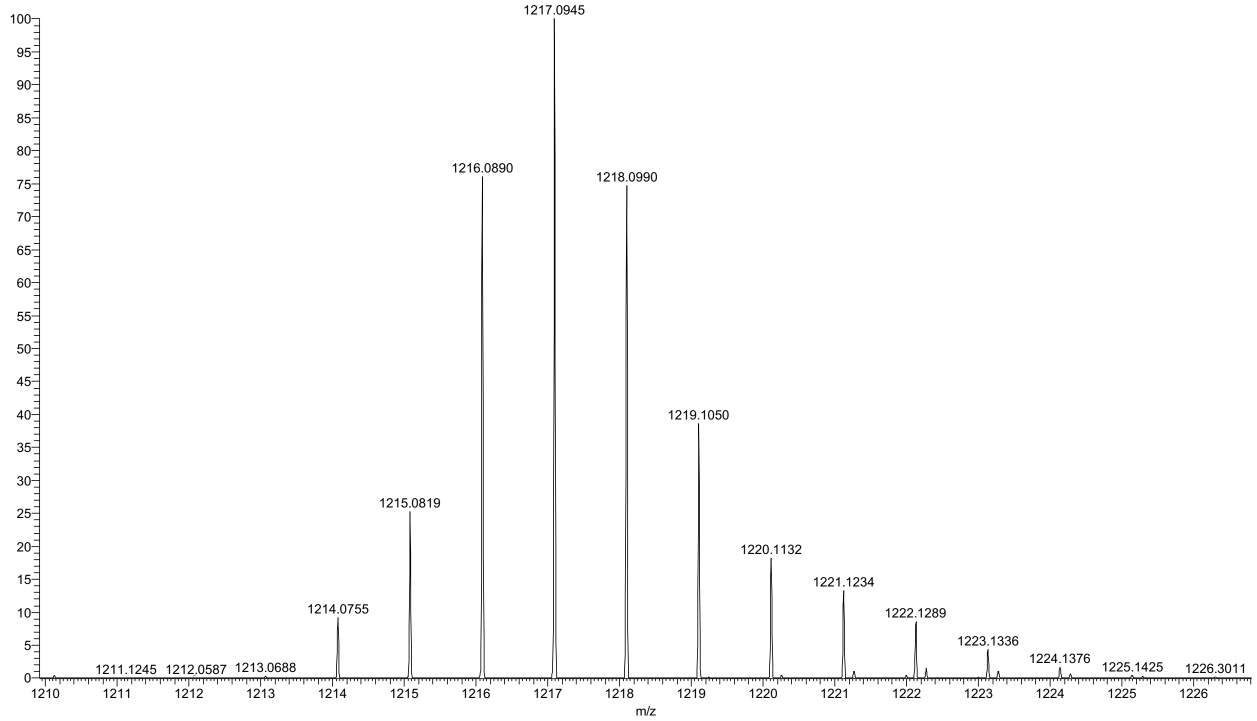

Figure S22: HRMS (+APCI) analysis of **12a/12b**, zoom-in on the catenane peaks.

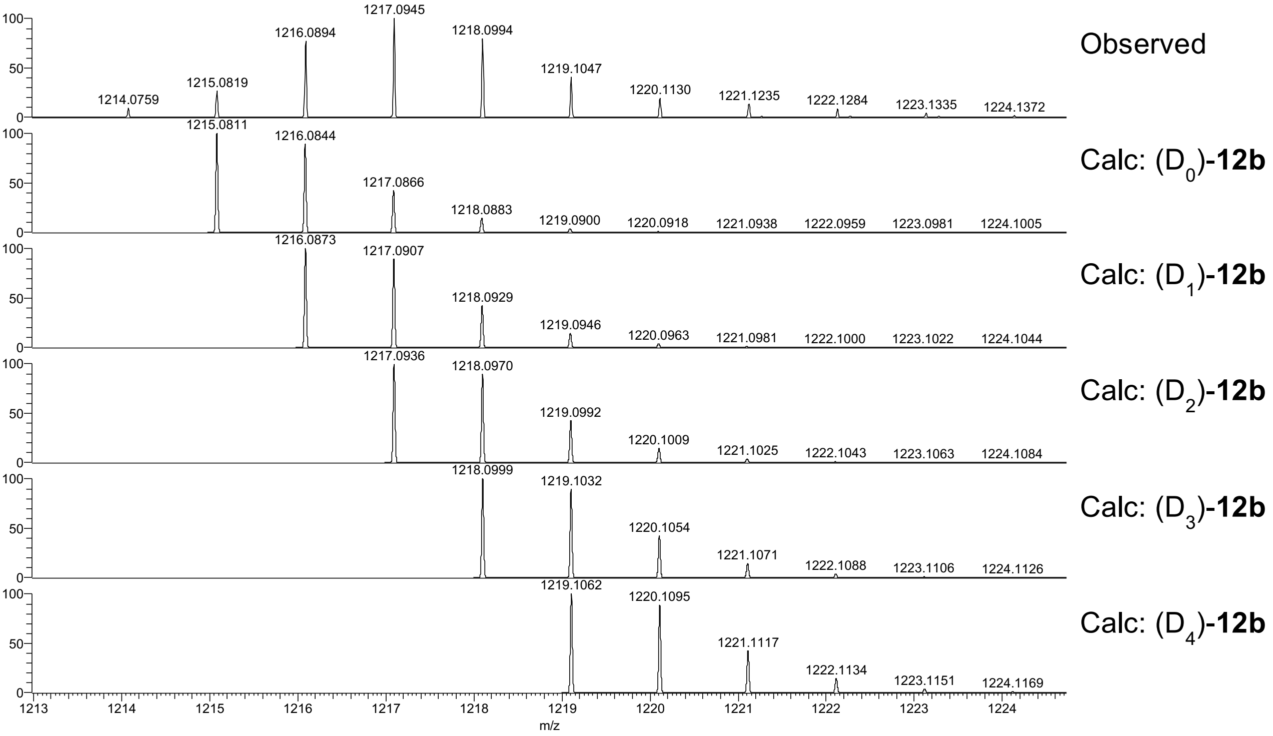

Figure S23: Stack-plot showing the observed HRMS (+APCI) spectrum of **12a**/**12b** (top) and the predicted spectra for the alkene species ($C_{80}H_{144-x}D_xNO_4S$, $_{x=0–4}$ $[M+H]^+$).

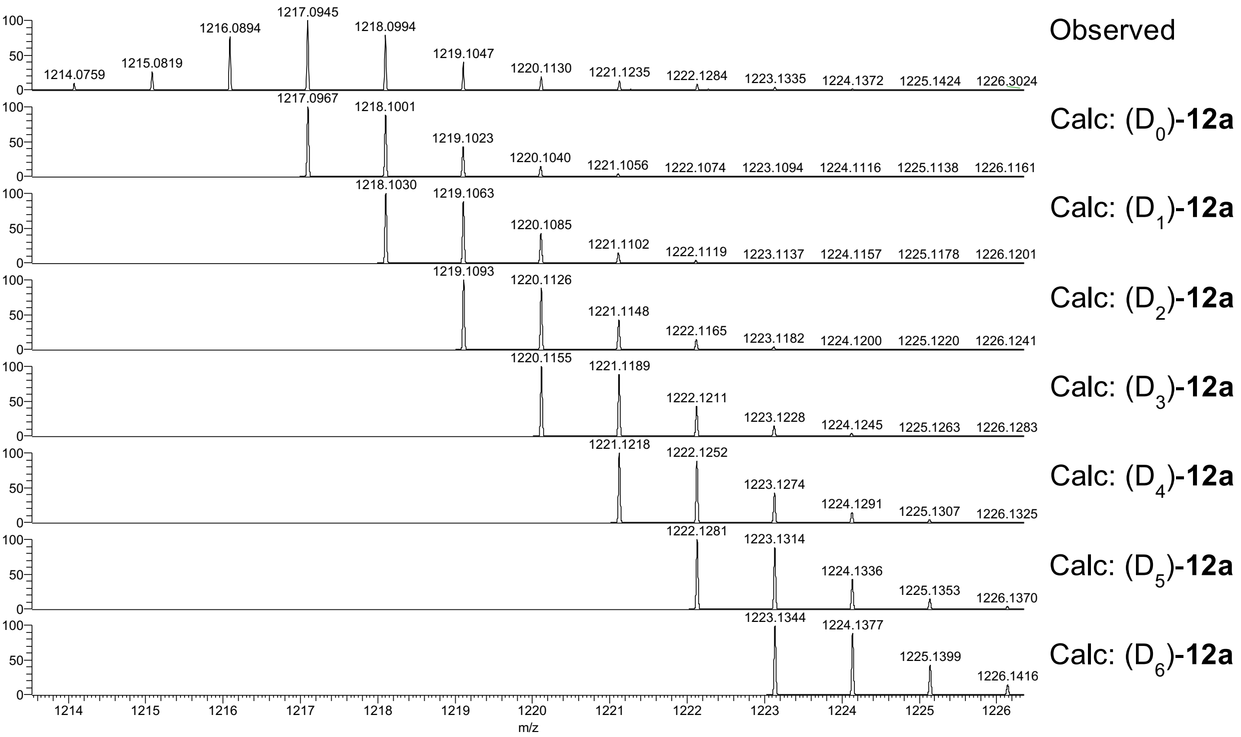

Figure S24: Stack-plot showing the observed HRMS (+APCI) spectrum of **12a**/**12b** (top) and the predicted spectra for the alkane species ($C_{80}H_{146-x}D_xNO_4S$, $_{x=0–6}$ $[M+H]^+$).

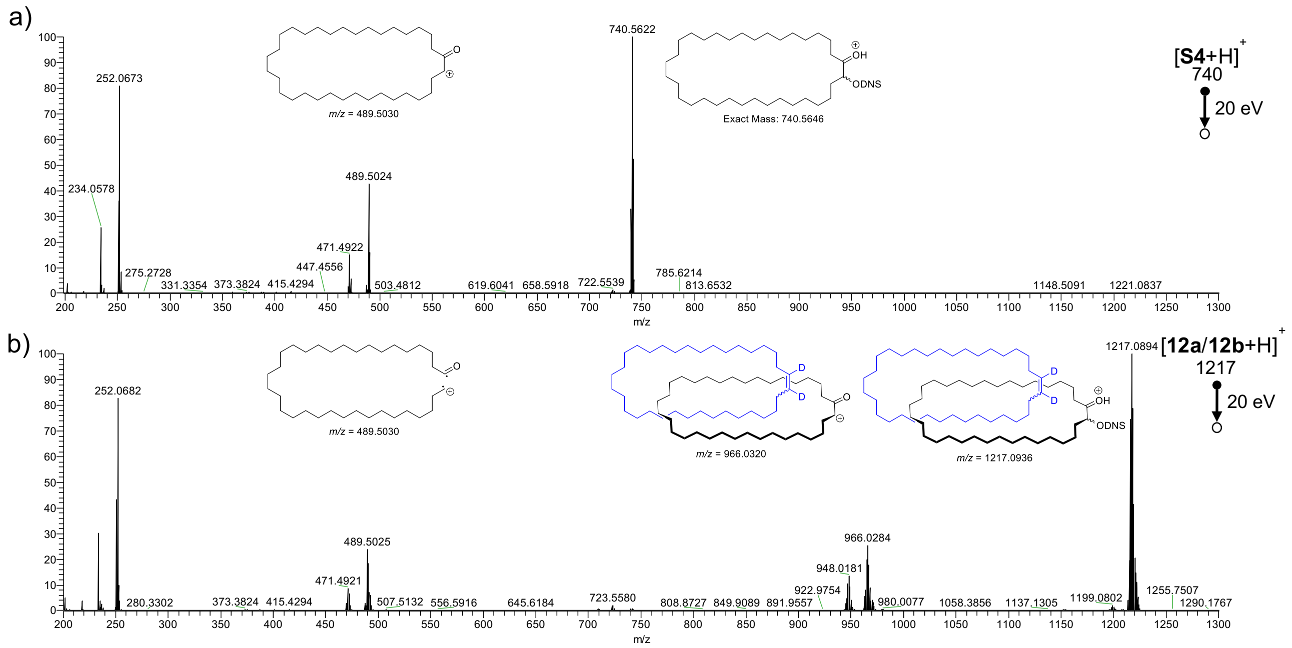

Figure 25. MS/MS (+APCI) of (a) Dansyl-acyloin at *m/z* = 740 ([**S4**+H]$^+$), using 20 eV collision energy; (b) isolated Dansyl-catenane at *m/z* = 1219 ± 5 Da ([**12a**/**12b**+H]$^+$), using 20 eV collision energy.

9. NMR spectra

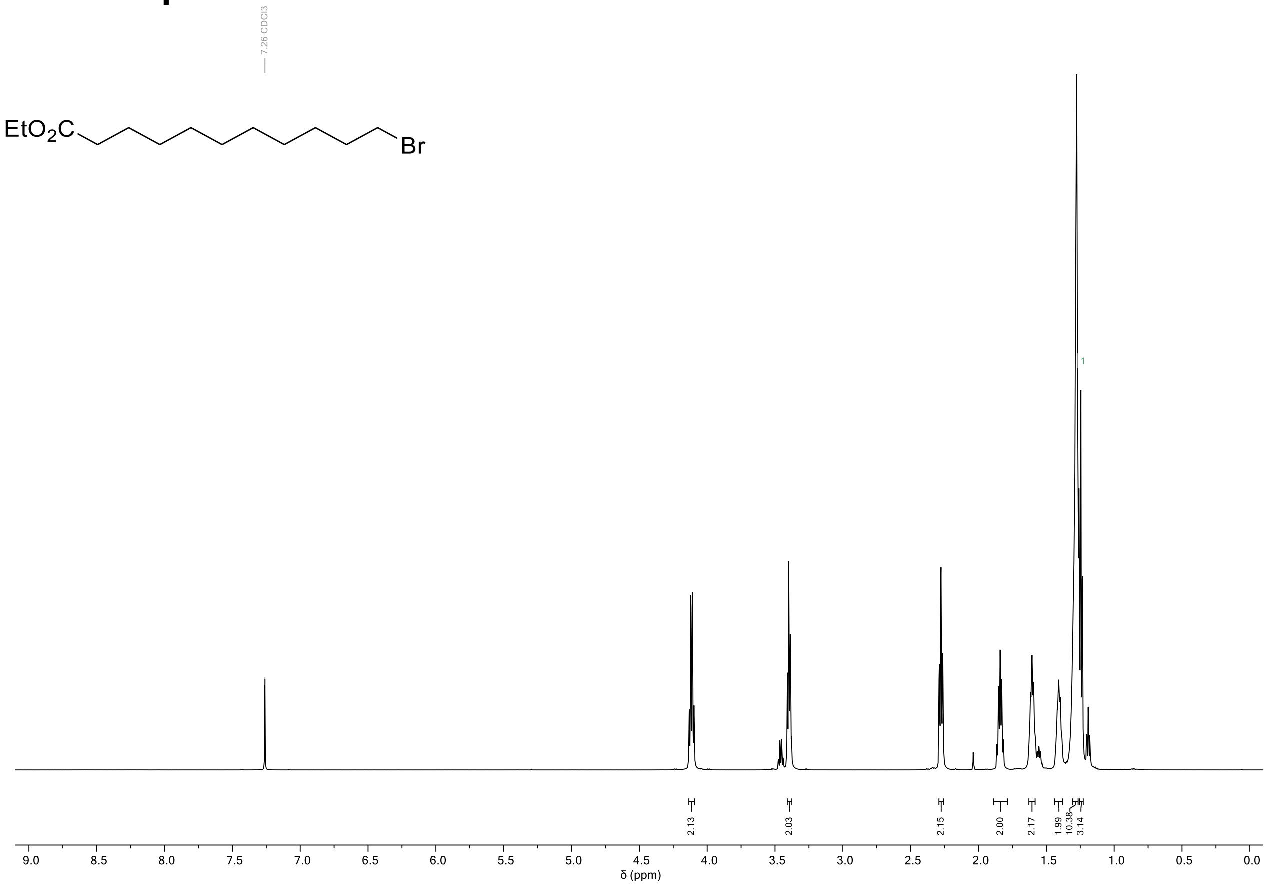

Spectrum S1: ^{1}H NMR (600 MHz, 298 K, $CDCl_3$) of **S1**

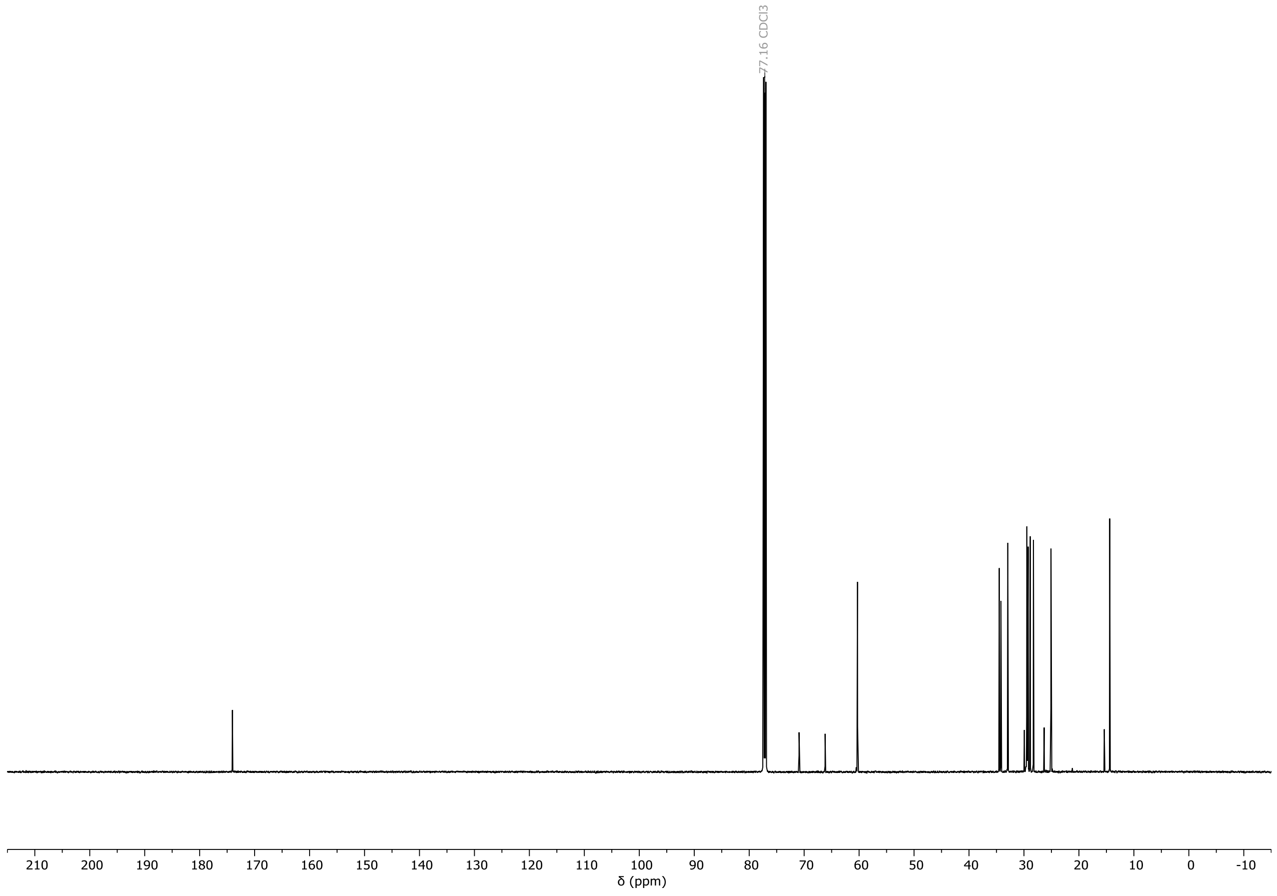

Spectrum S2: ^{13}C NMR (151 MHz, 298 K, $CDCl_3$) of **S1**

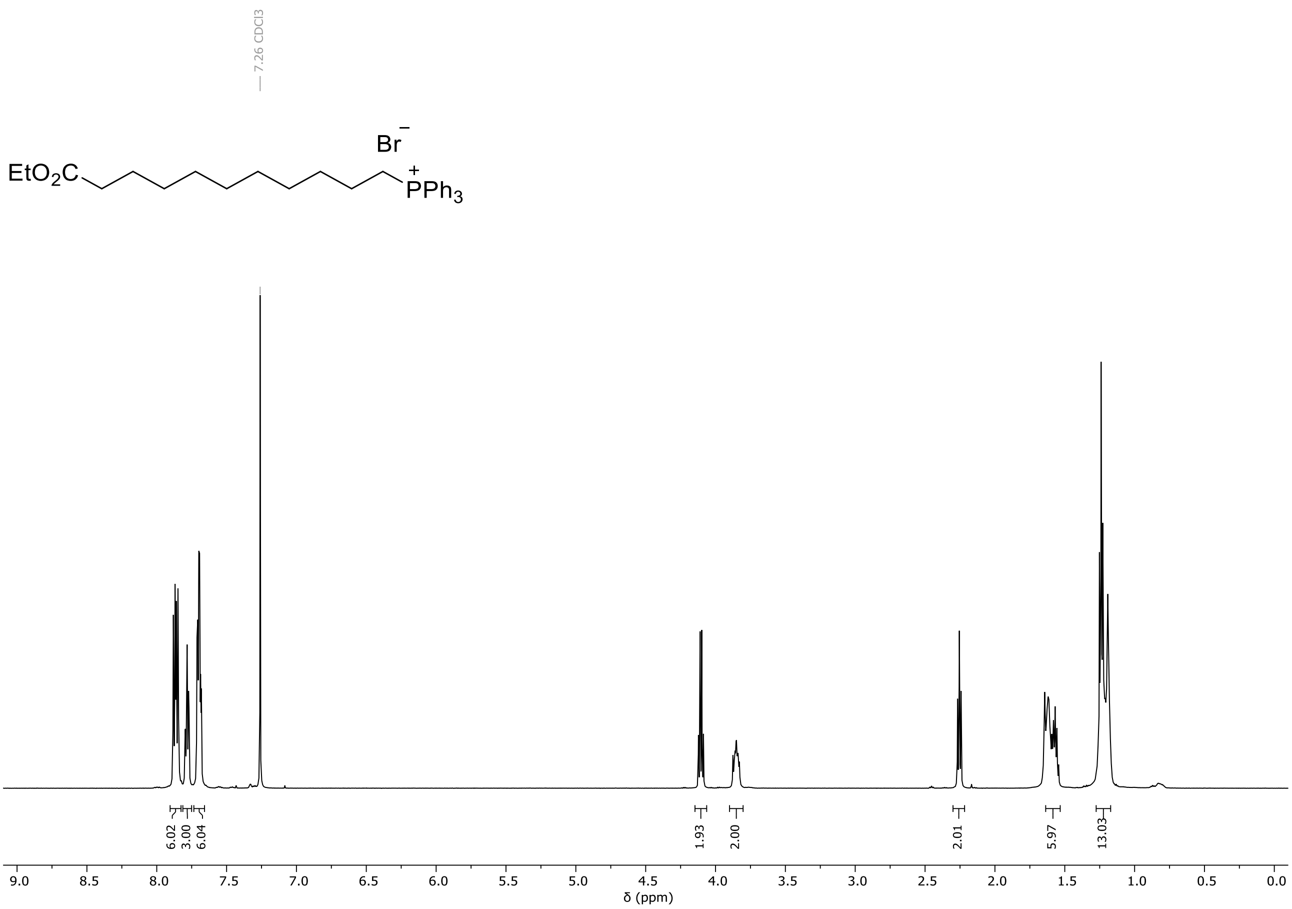

Spectrum S3: ^{1}H NMR (600 MHz, 298 K, $CDCl_3$) of **7**

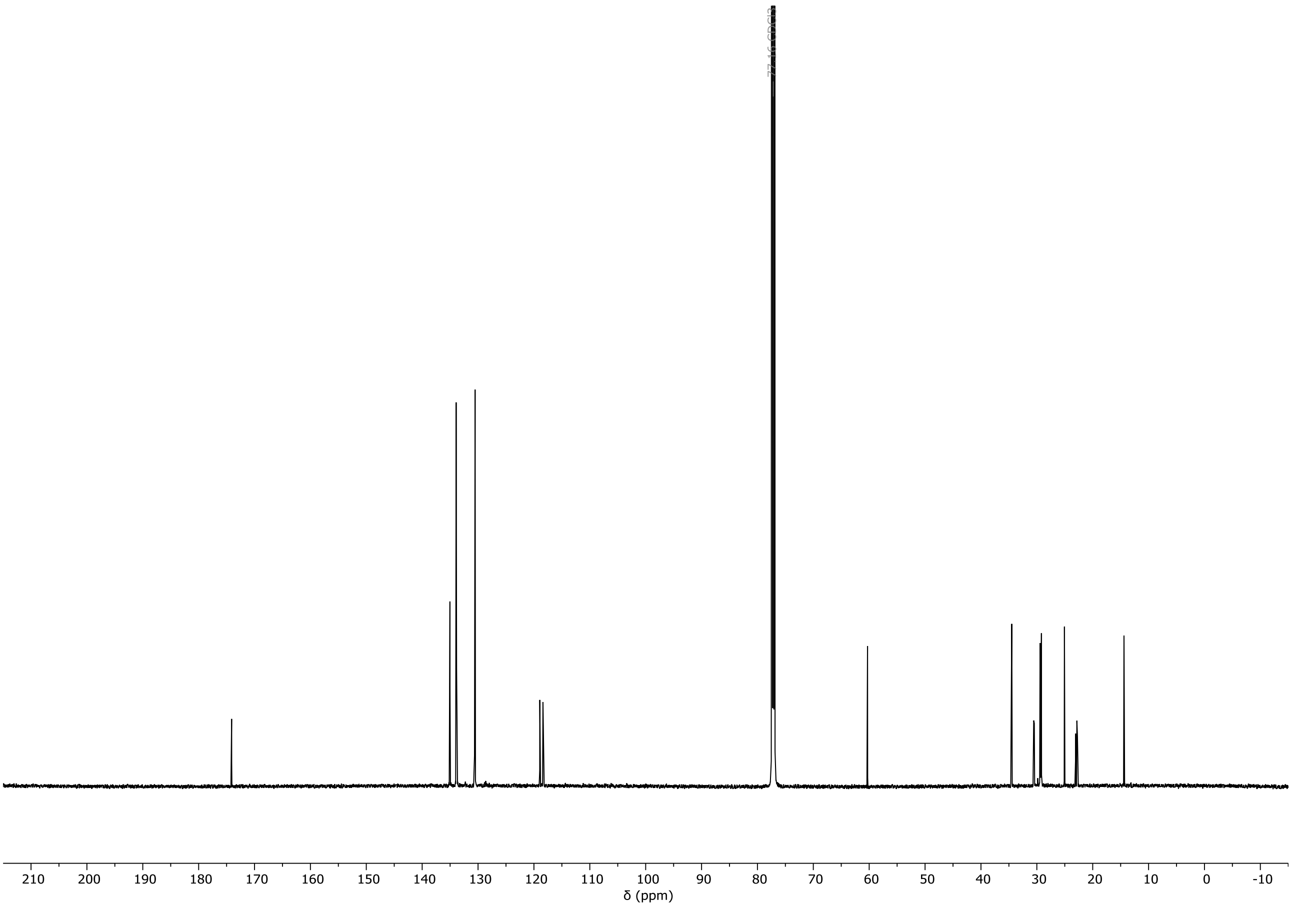

Spectrum S4: ^{13}C NMR (151 MHz, 298 K, $CDCl_3$) of **7**

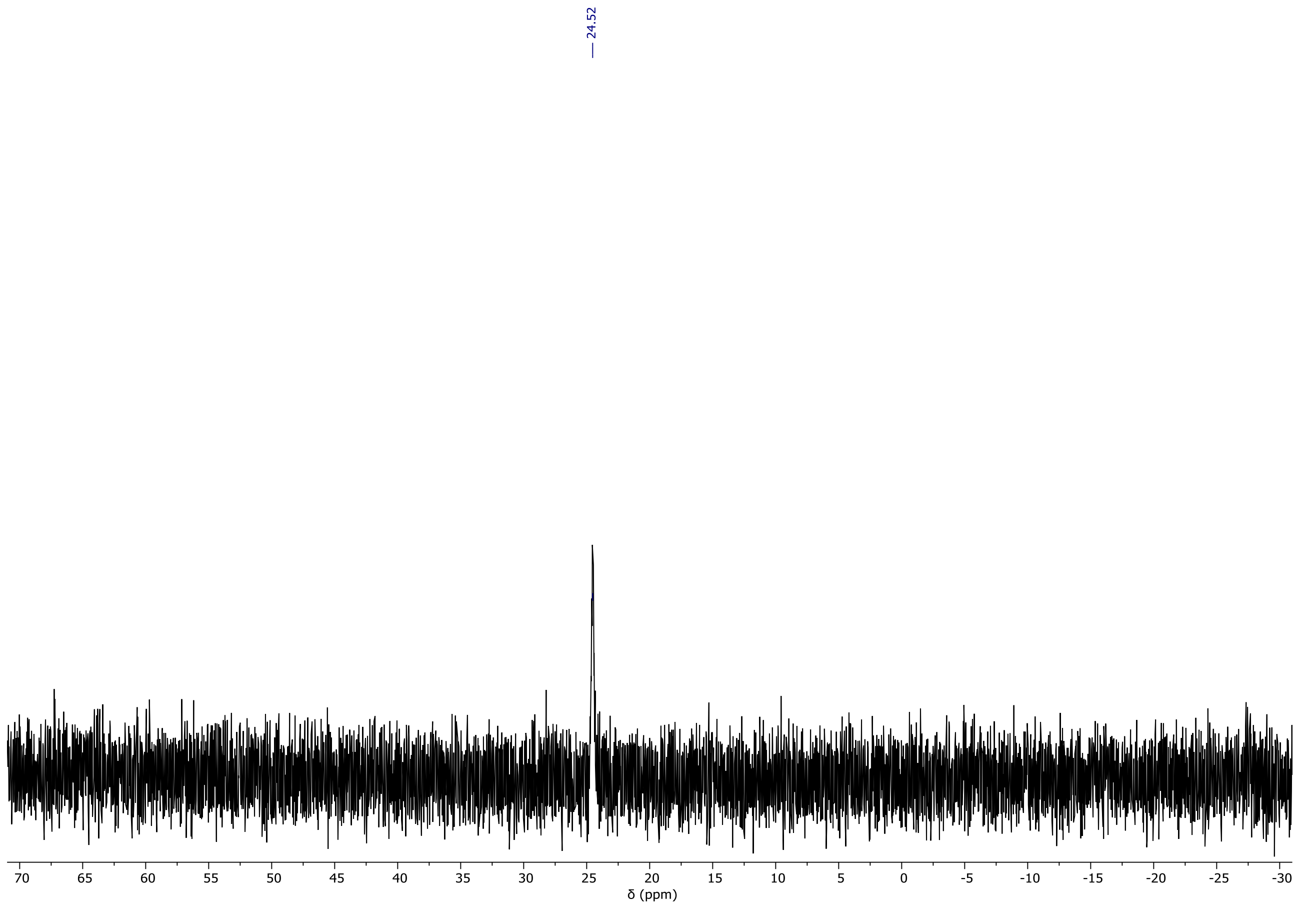

Spectrum S5: ^{31}P NMR (162 MHz, 298 K, $CDCl_3$) of **7**

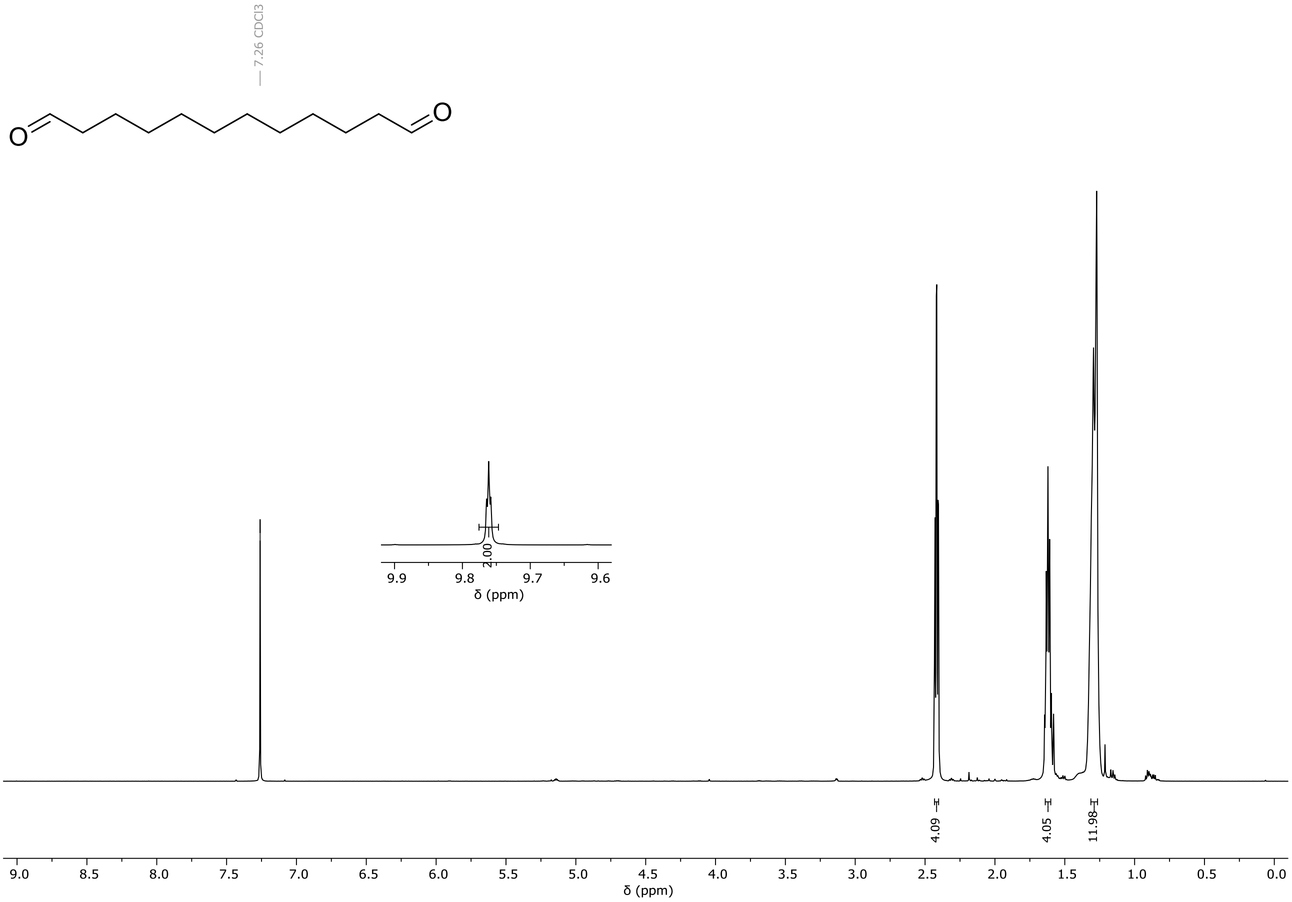

Spectrum S6: ^{1}H NMR (600 MHz, 298 K, $CDCl_3$) of **6**

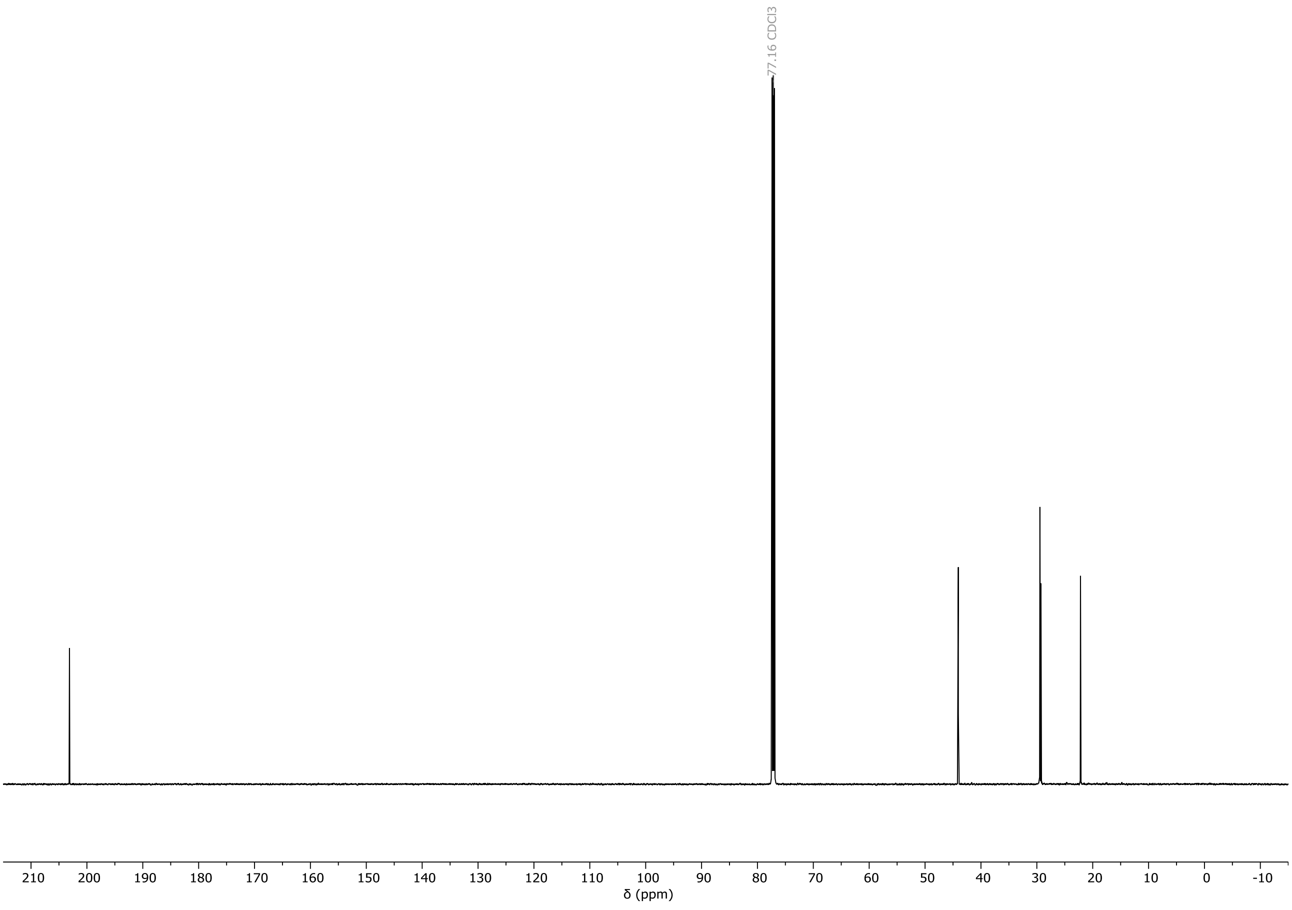

Spectrum S7: ^{13}C NMR (151 MHz, 298 K, $CDCl_3$) of **6**

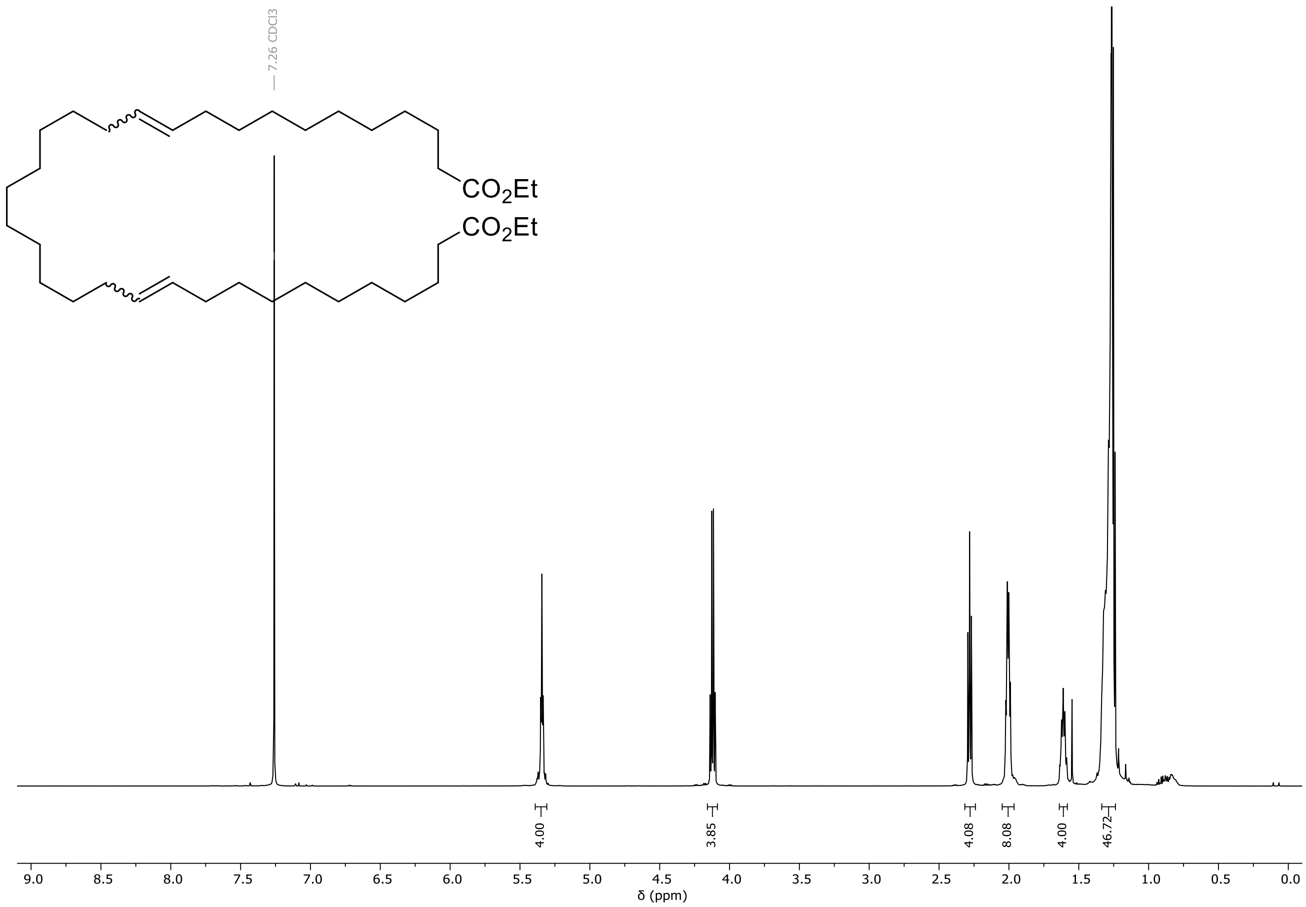

Spectrum S8: ^{1}H NMR (600 MHz, 298 K, $CDCl_3$) of **S2**

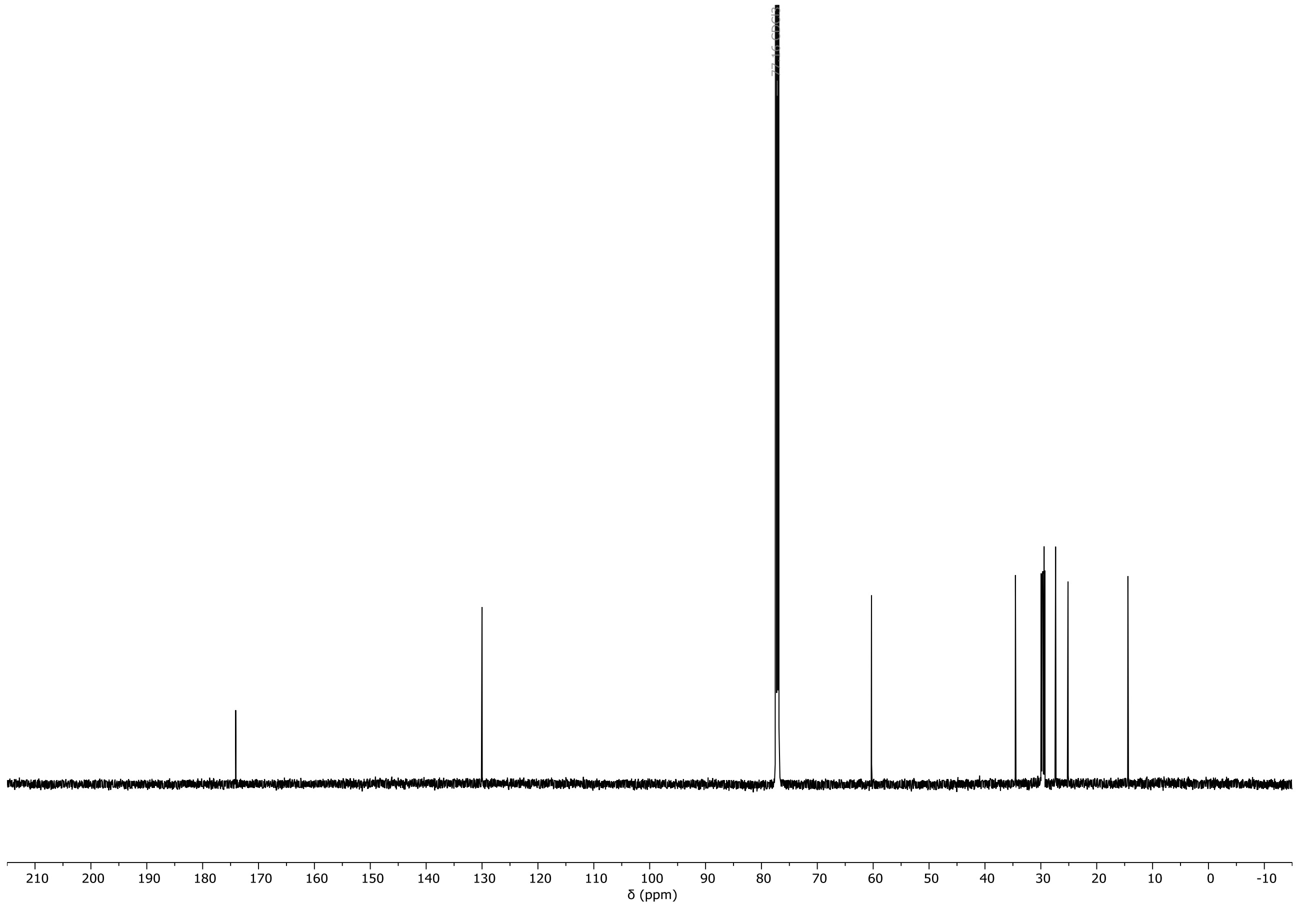

Spectrum S9: ^{13}C NMR (151 MHz, 298 K, $CDCl_3$) of **S2**

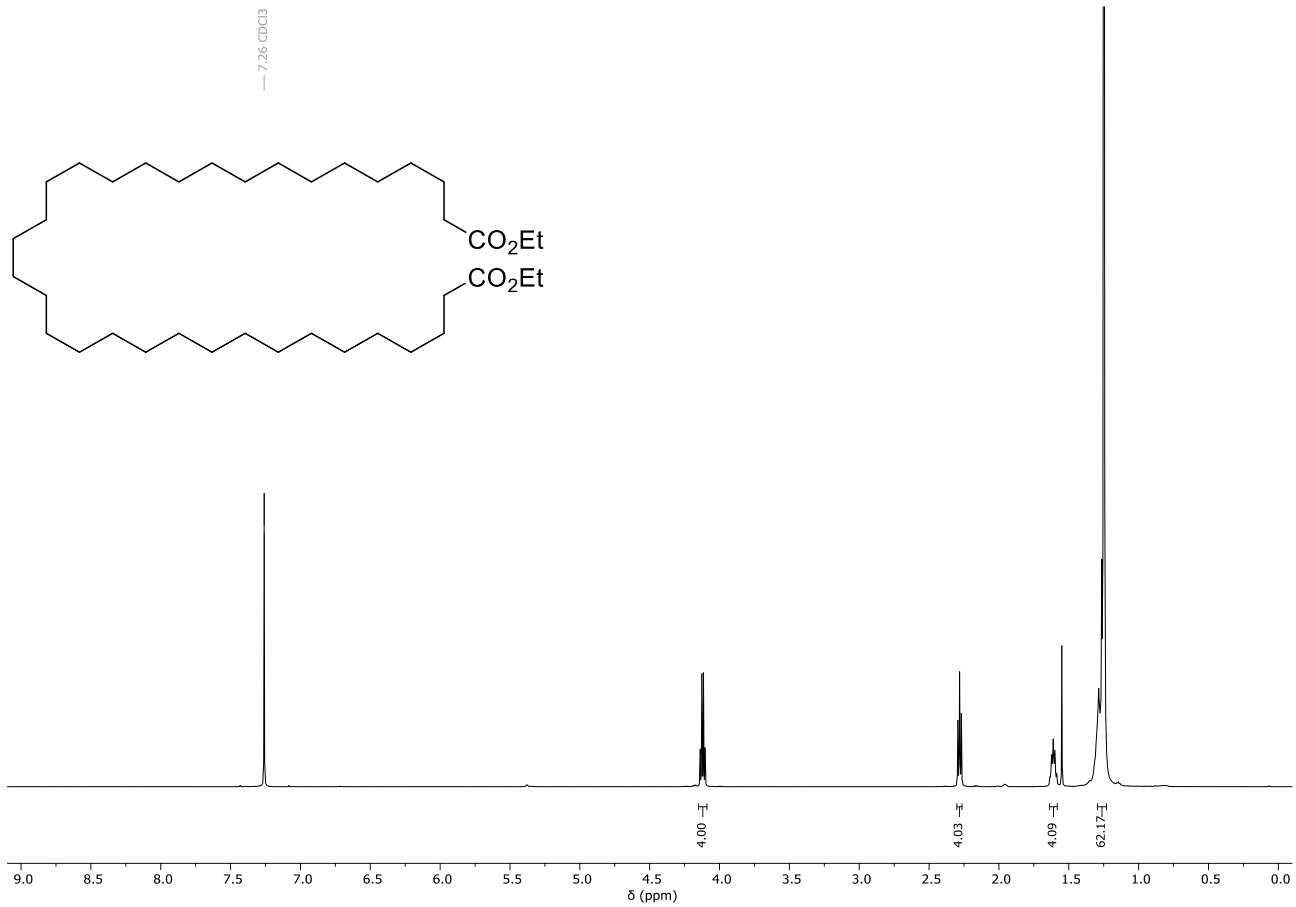

Spectrum S10: ^{1}H NMR (600 MHz, 298 K, $CDCl_3$) of **2**

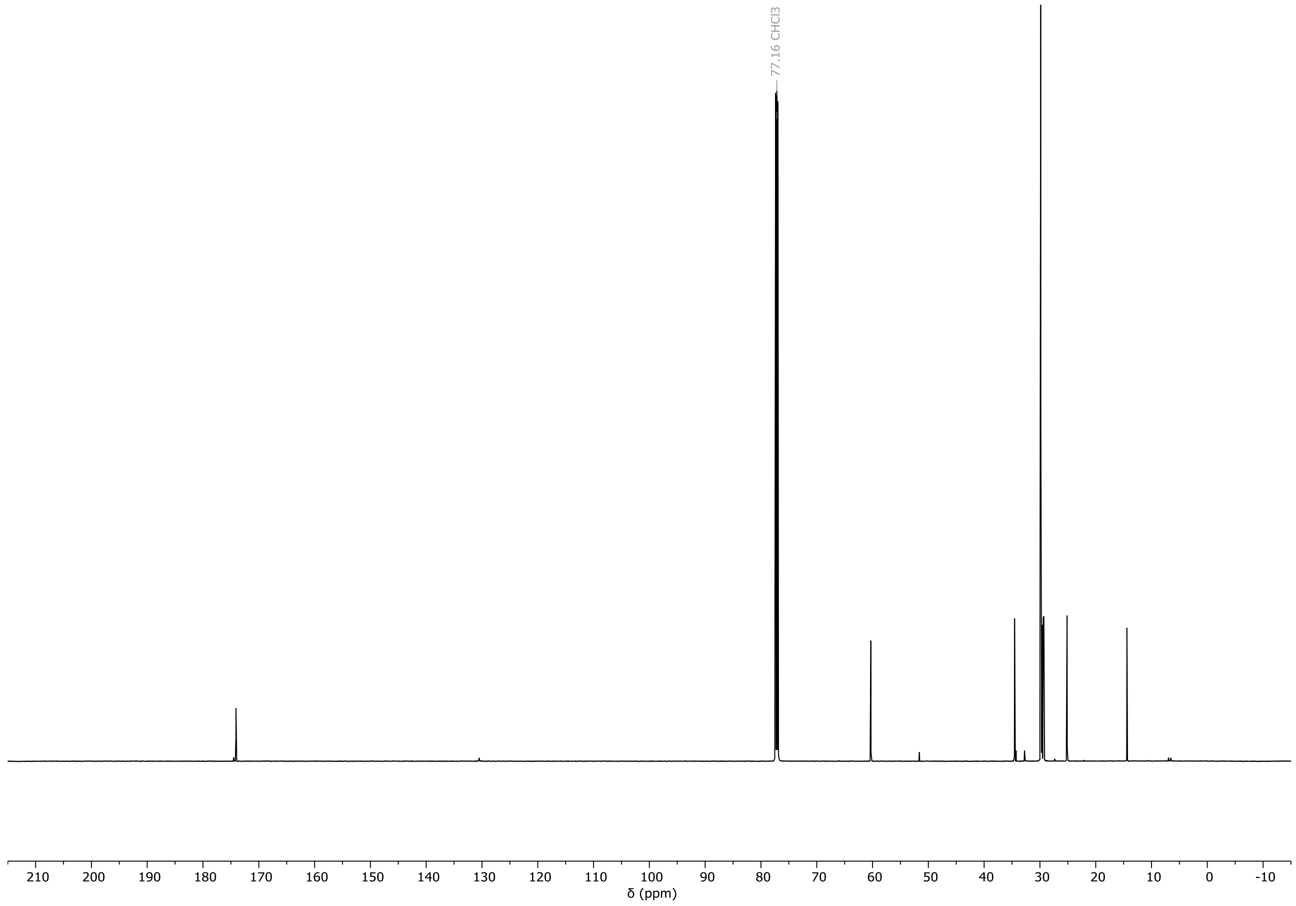

Spectrum S11: ^{13}C NMR (151 MHz, 298 K, $CDCl_3$) of **2**

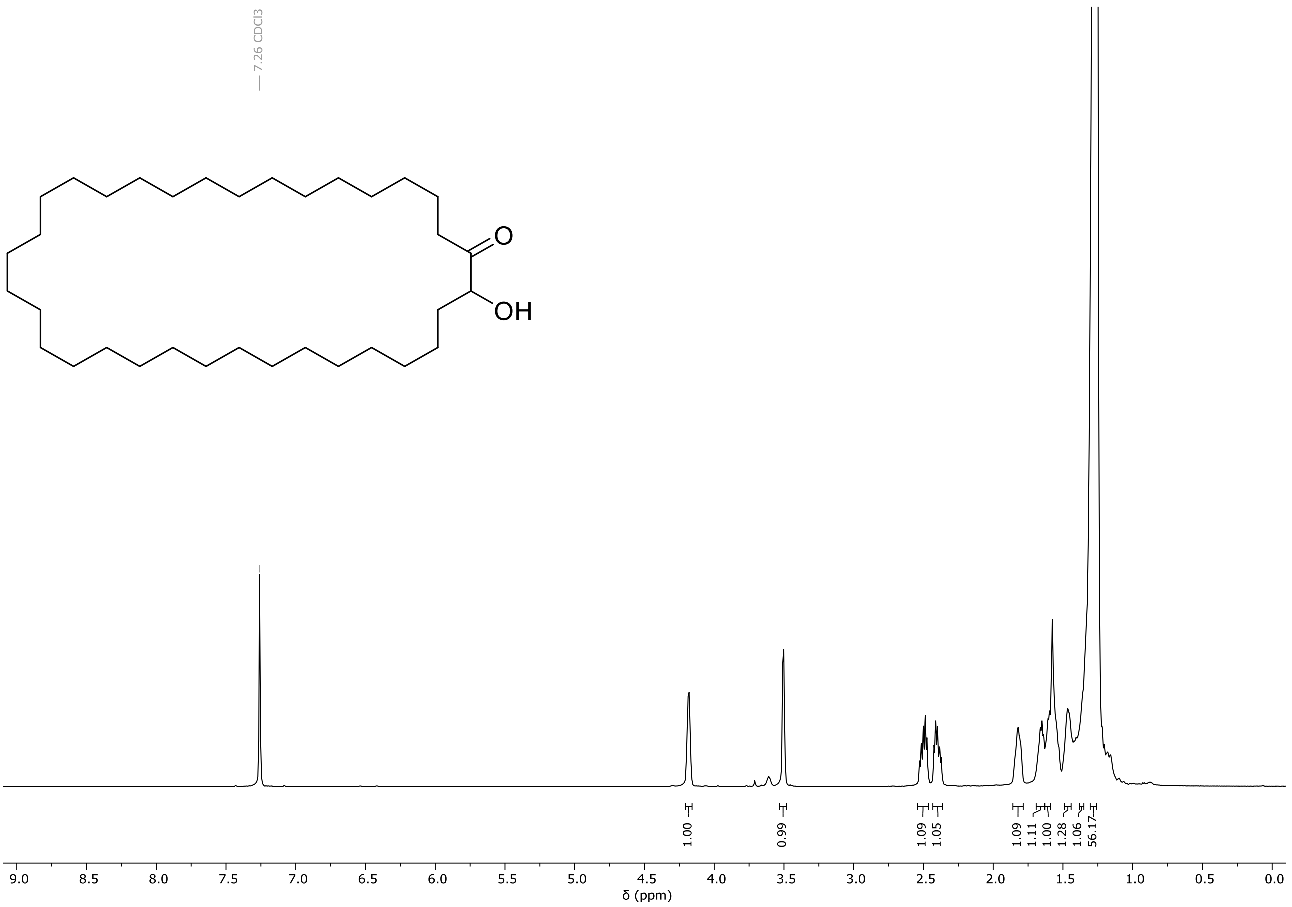

Spectrum S12: ^{1}H NMR (600 MHz, 298 K, $CDCl_3$) of **4**

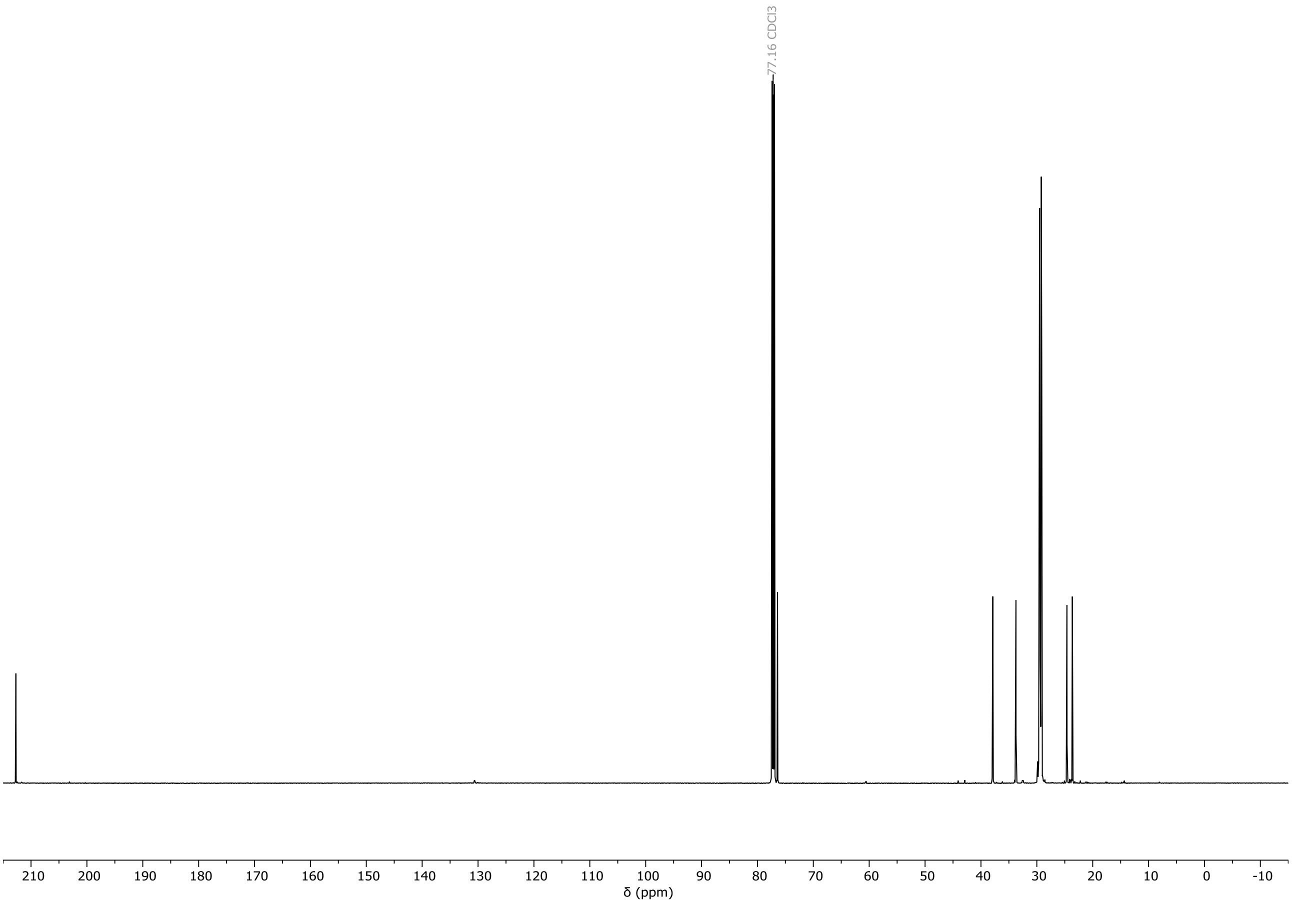

Spectrum S13: ^{13}C NMR (151 MHz, 298 K, $CDCl_3$) of **4**

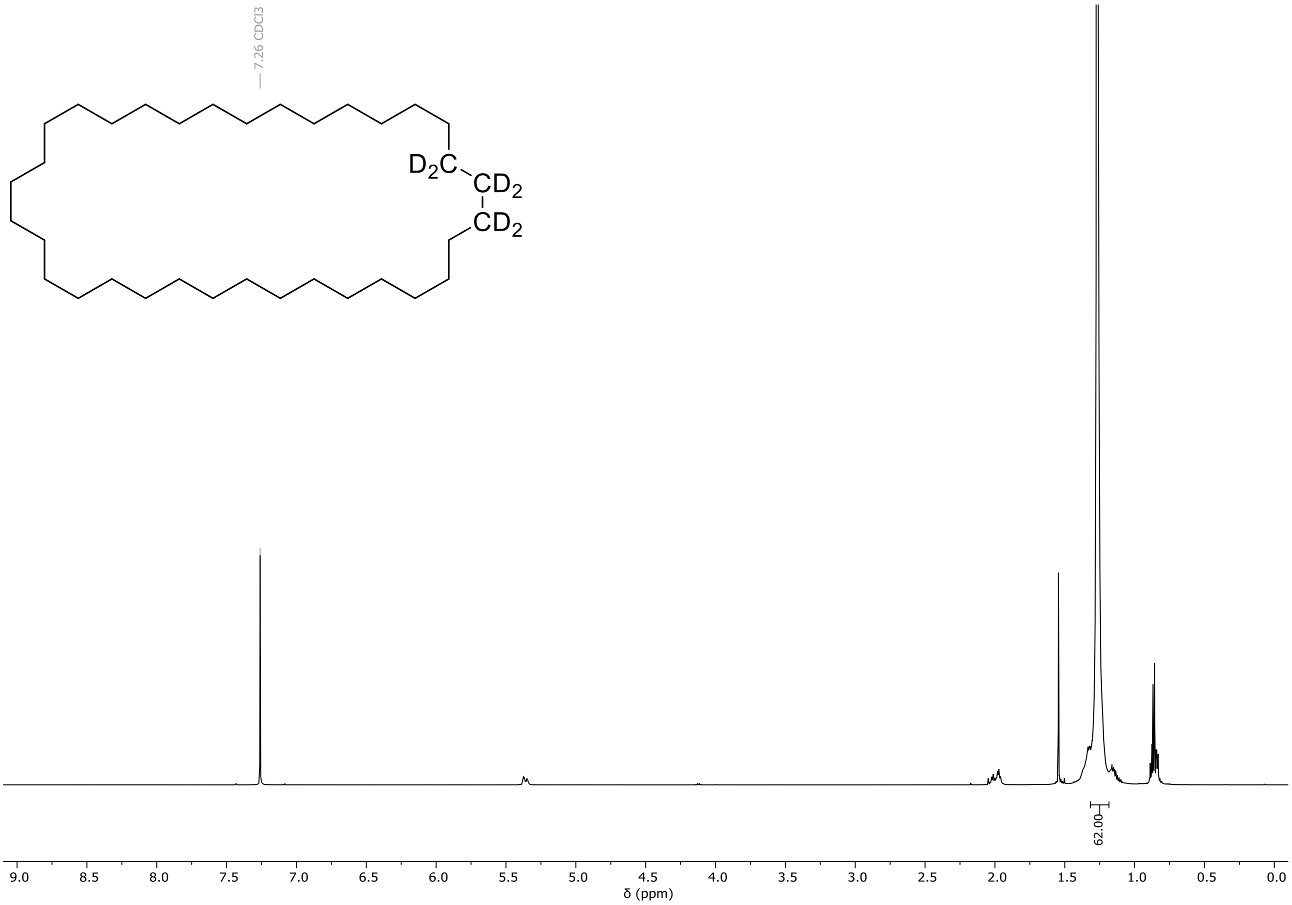

Spectrum S14: ^{1}H NMR (600 MHz, 298 K, $CDCl_3$) of **3/10**

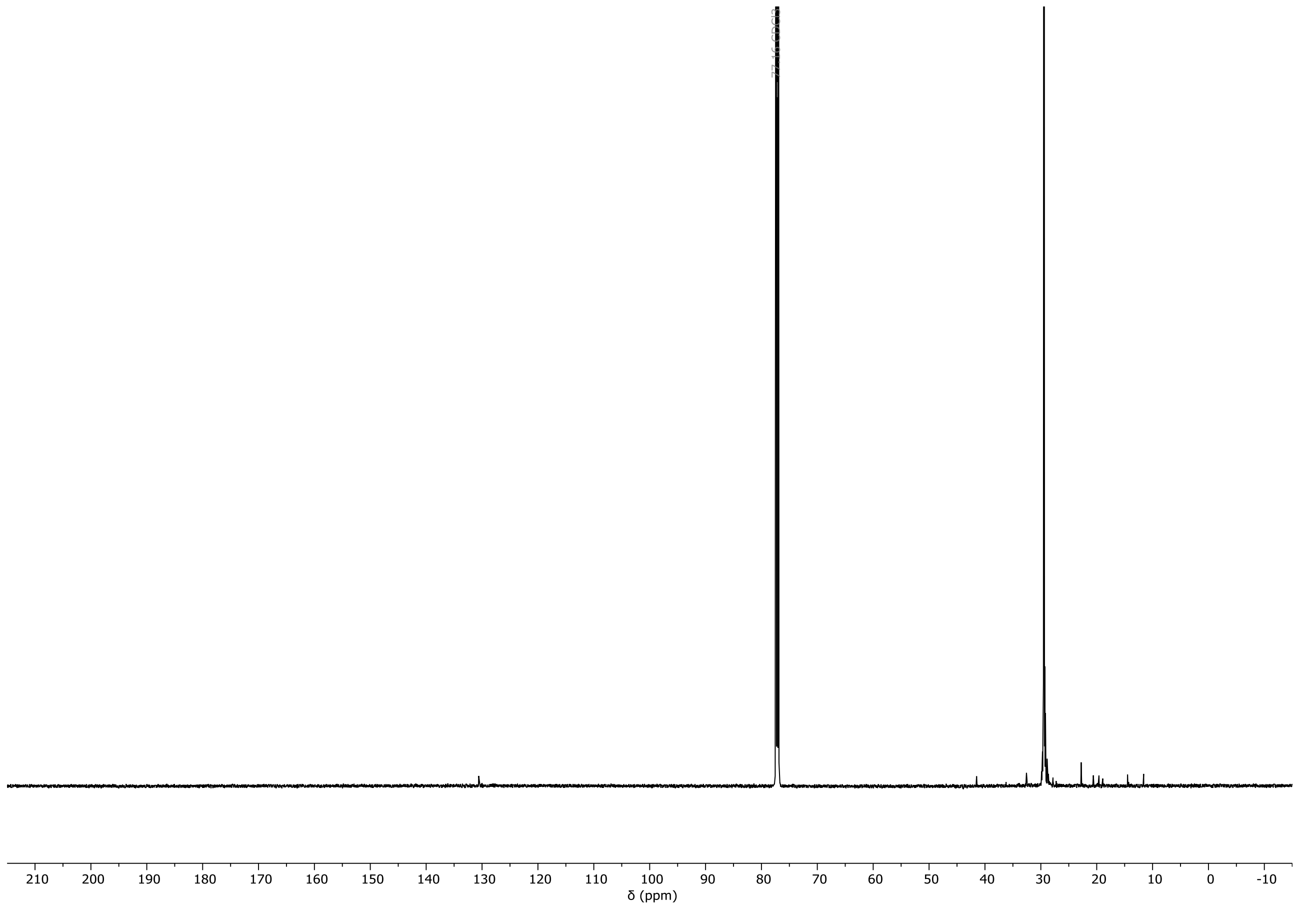

Spectrum S15: ^{13}C NMR (151 MHz, 298 K, $CDCl_3$) of **3/10**

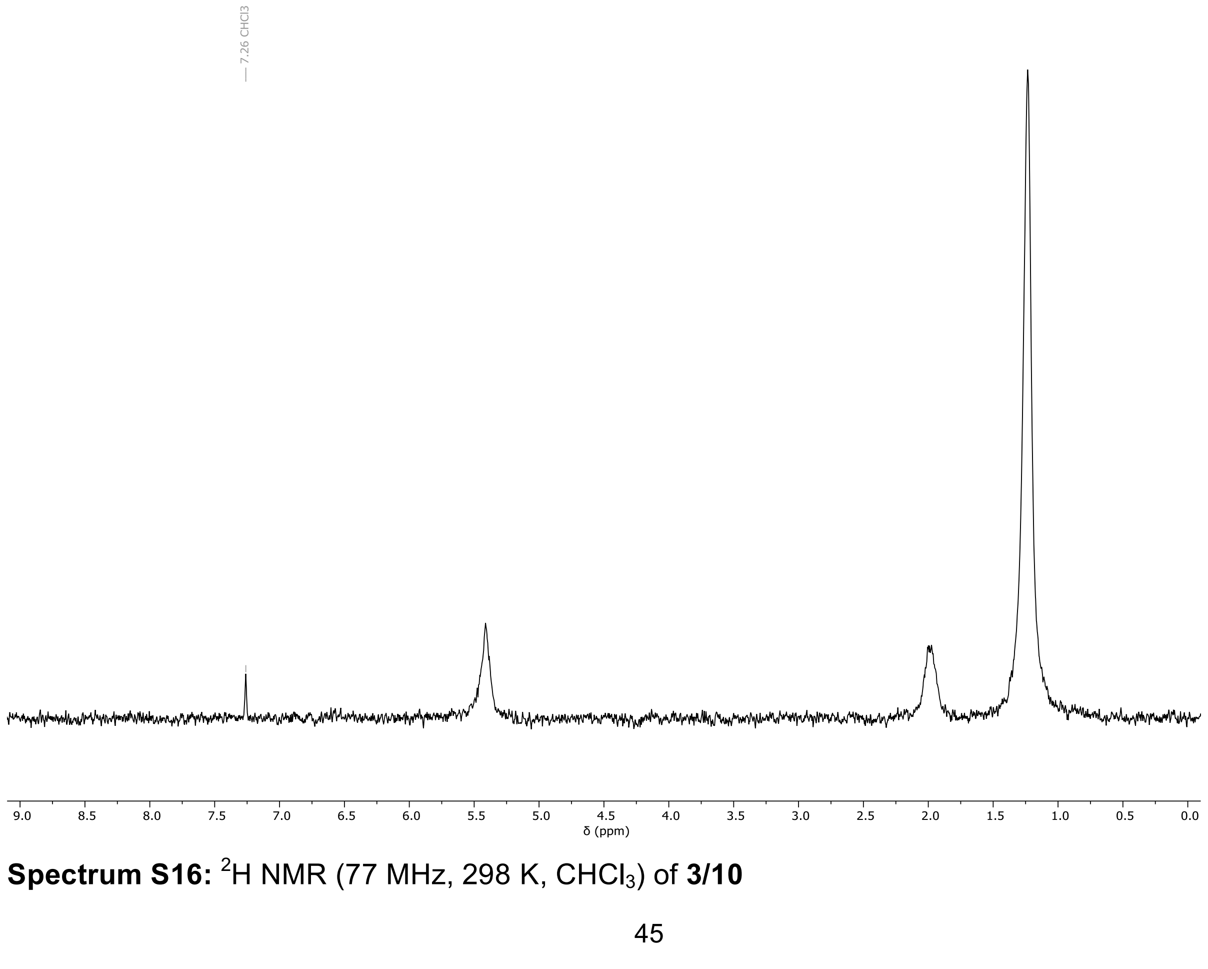

Spectrum S16: ^{2}H NMR (77 MHz, 298 K, $CHCl_3$) of **3/10**

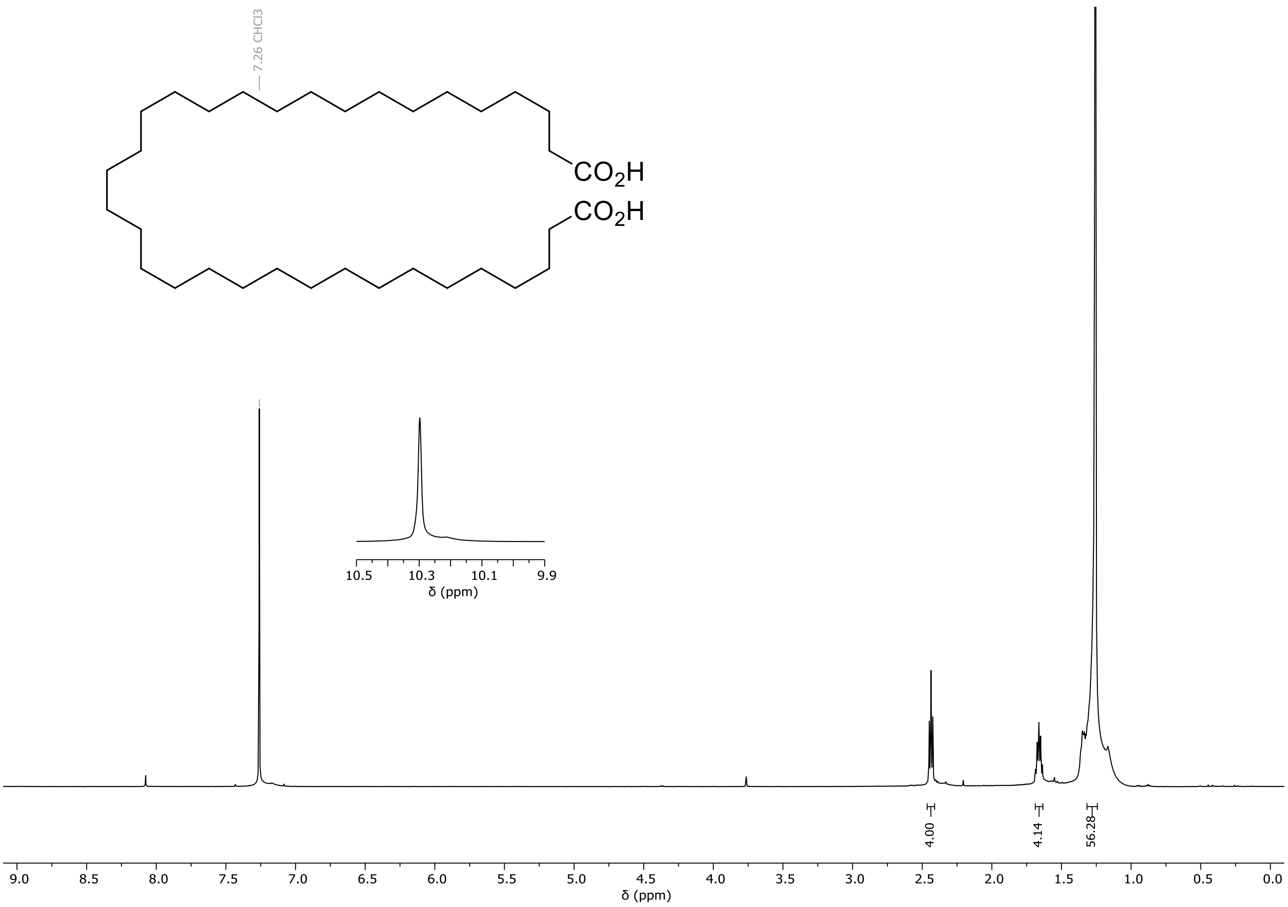

Spectrum S17: ^{1}H NMR (600 MHz, 298 K, $CDCl_3/CF_3CO_2H$) of **5**.

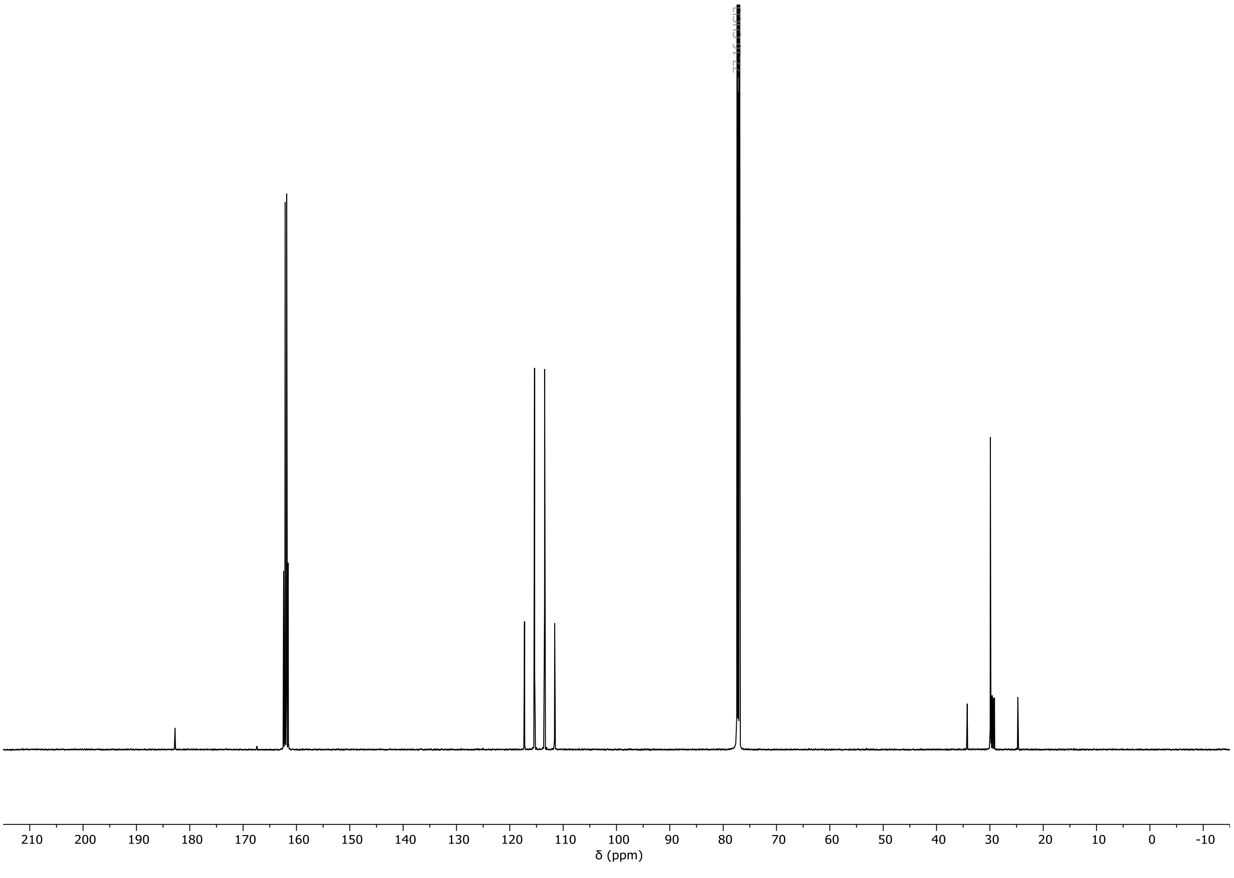

Spectrum S18: ^{13}C NMR (151 MHz, 298 K, $CDCl_3/CF_3CO_2H$) of **5**.

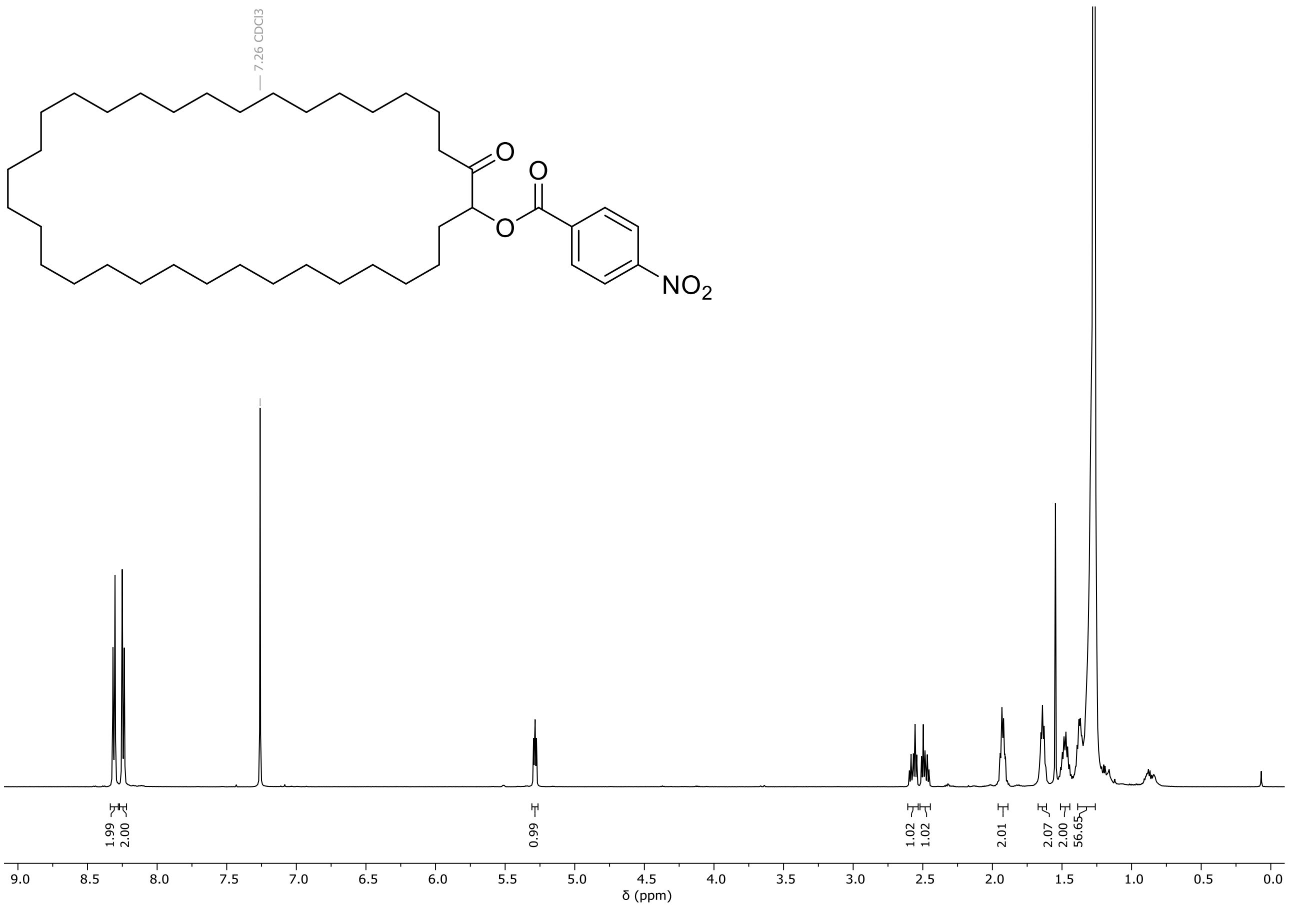

Spectrum S19: ^{1}H NMR (600 MHz, 298 K, $CDCl_3$) of **S3**.

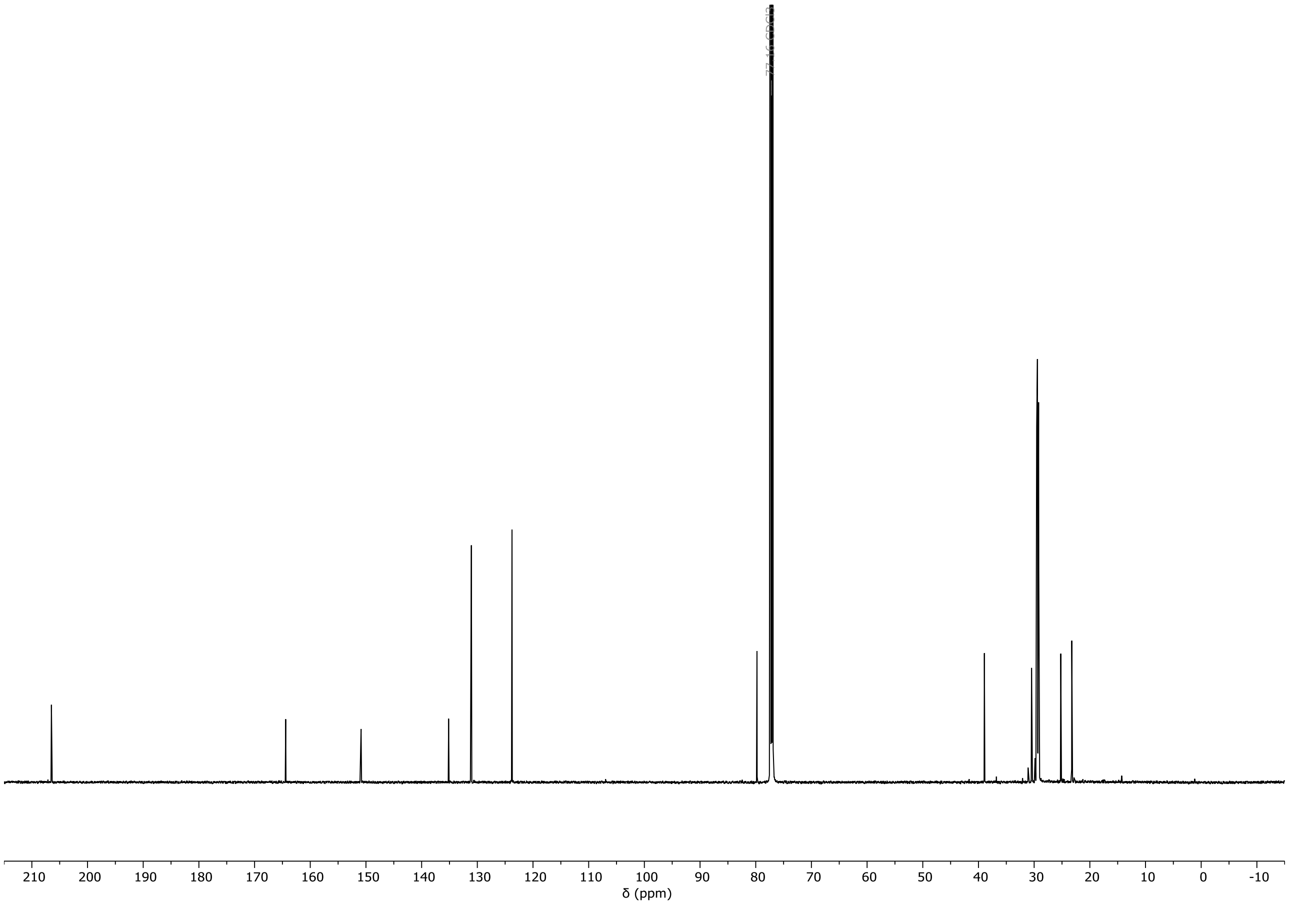

Spectrum S20: ^{13}C NMR (151 MHz, 298 K, $CDCl_3$) of **S3**.

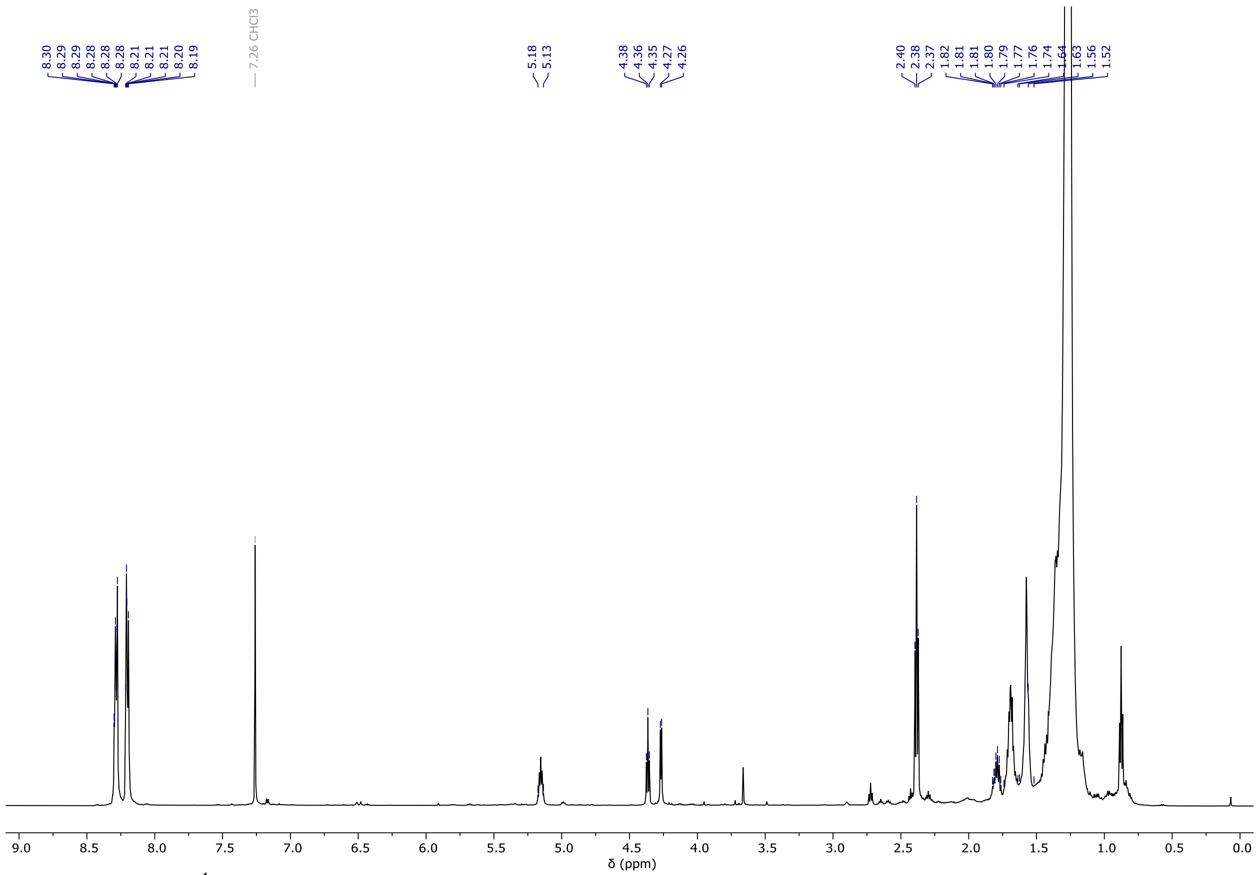

Spectrum S21. ^{1}H NMR (600 MHz, 298 K, $CDCl_3$) of product mixture **S5**, **S10a**, **S11** and **S12**.

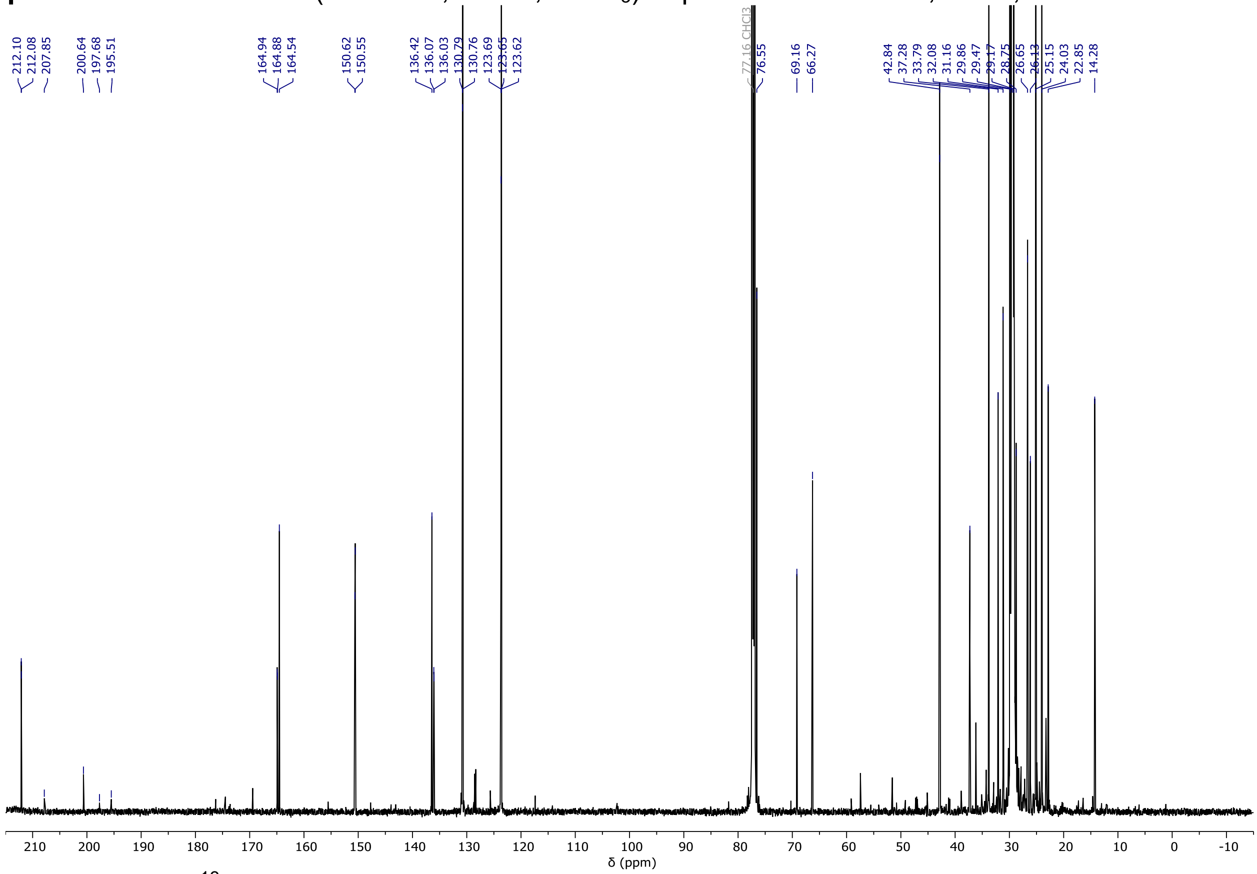

Spectrum S22. ^{13}C NMR (151 MHz, 298 K, $CDCl_3$) of product mixture **S5**, **S10a**, **S11** and **S12**.

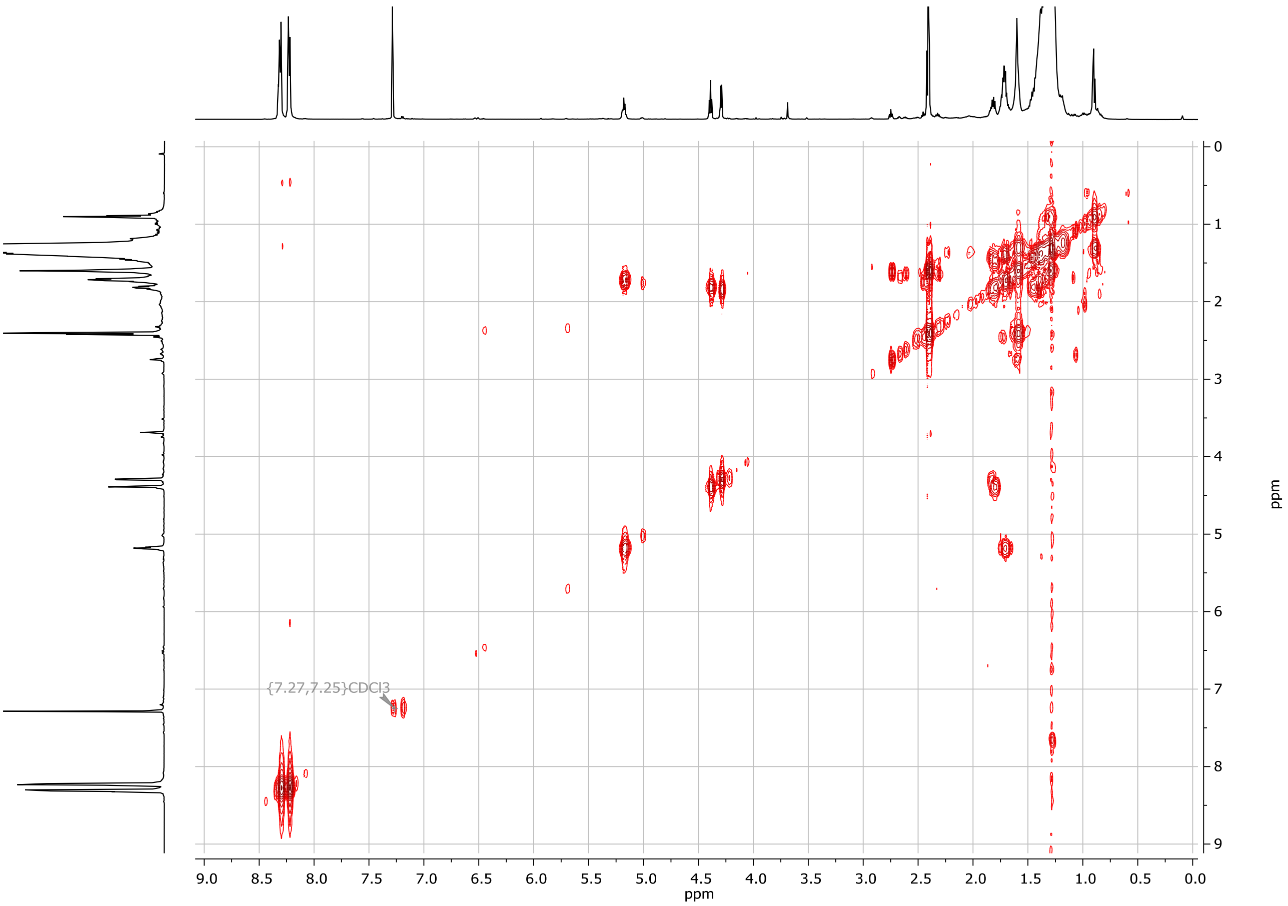

Spectrum S23. COSY of product mixture **S5**, **S10a**, **S11** and **S12**.

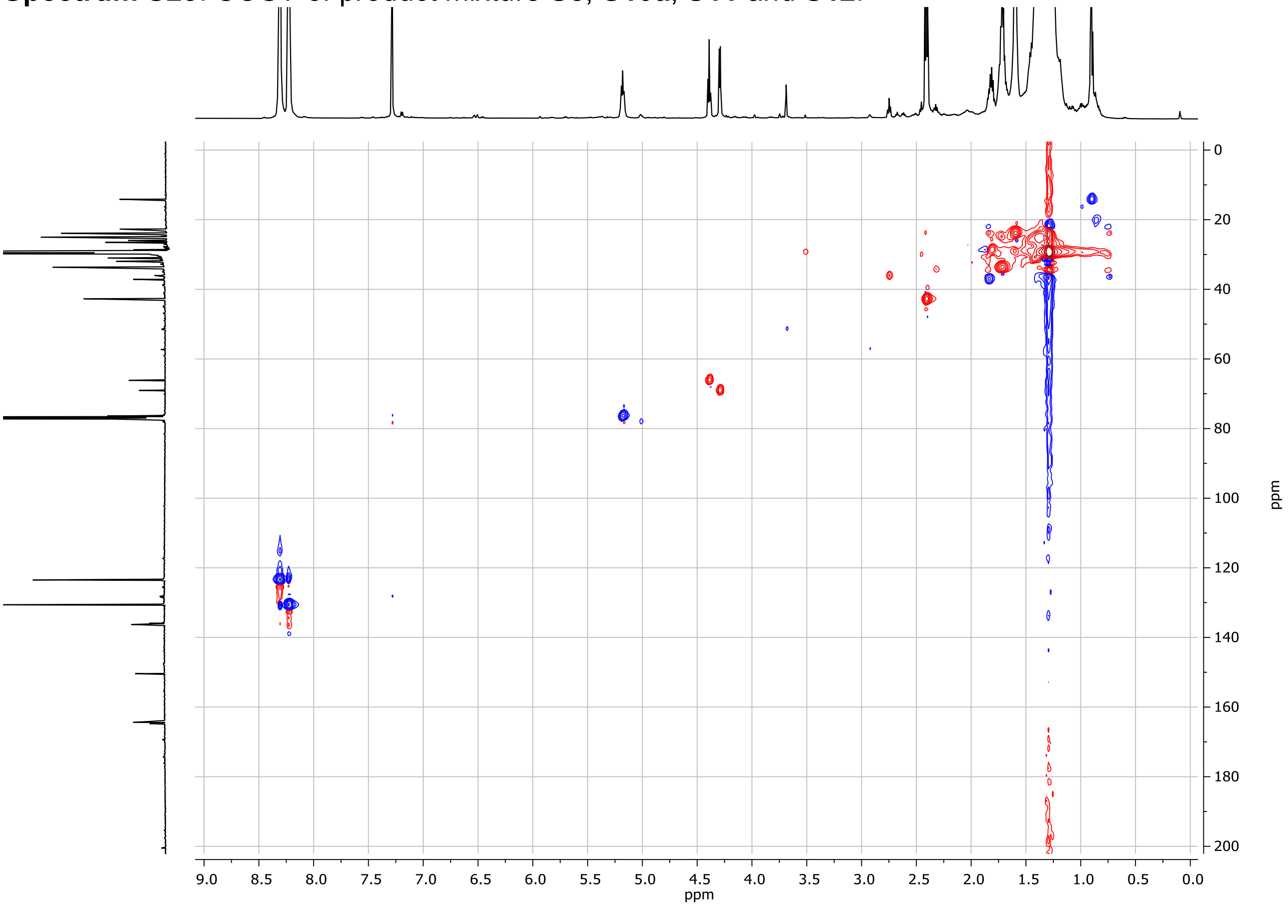

Spectrum S24. HSQC of product mixture **S5**, **S10a**, **S11** and **S12**.

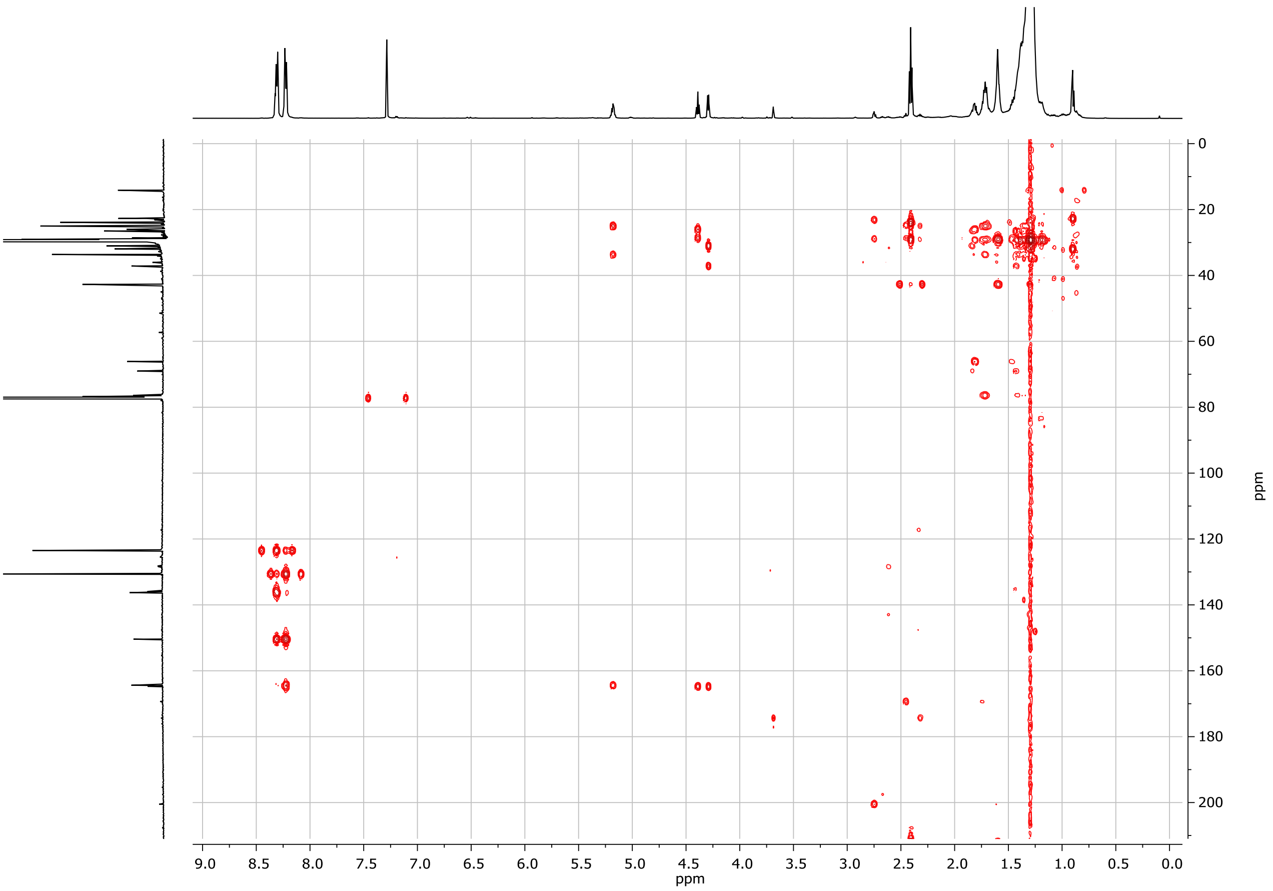

Spectrum S25. HMBC of product mixture **S5**, **S10a**, **S11** and **S12**.

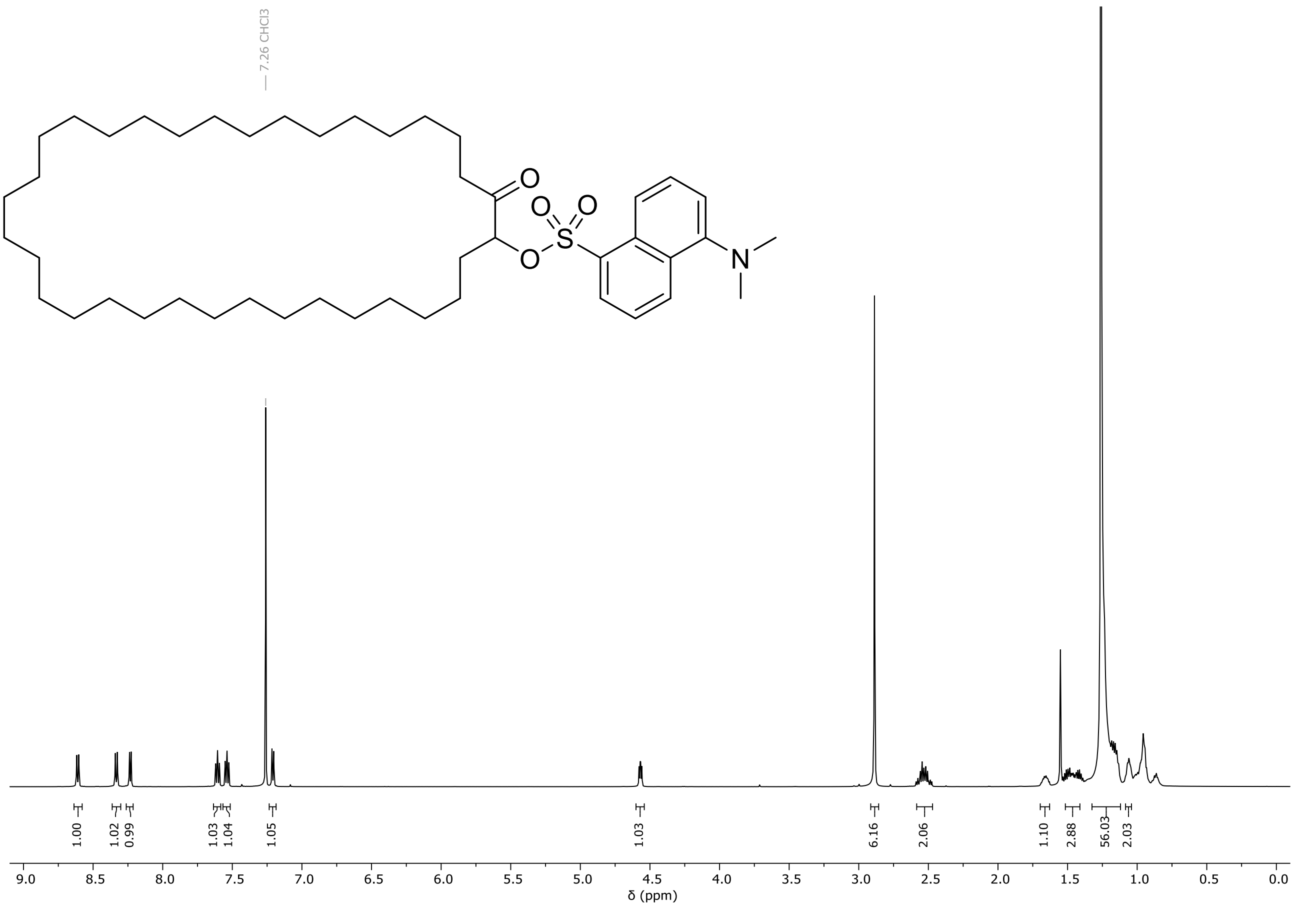

Spectrum S26: ^{1}H NMR (600 MHz, 298 K, $CDCl_3$) of **S4**.

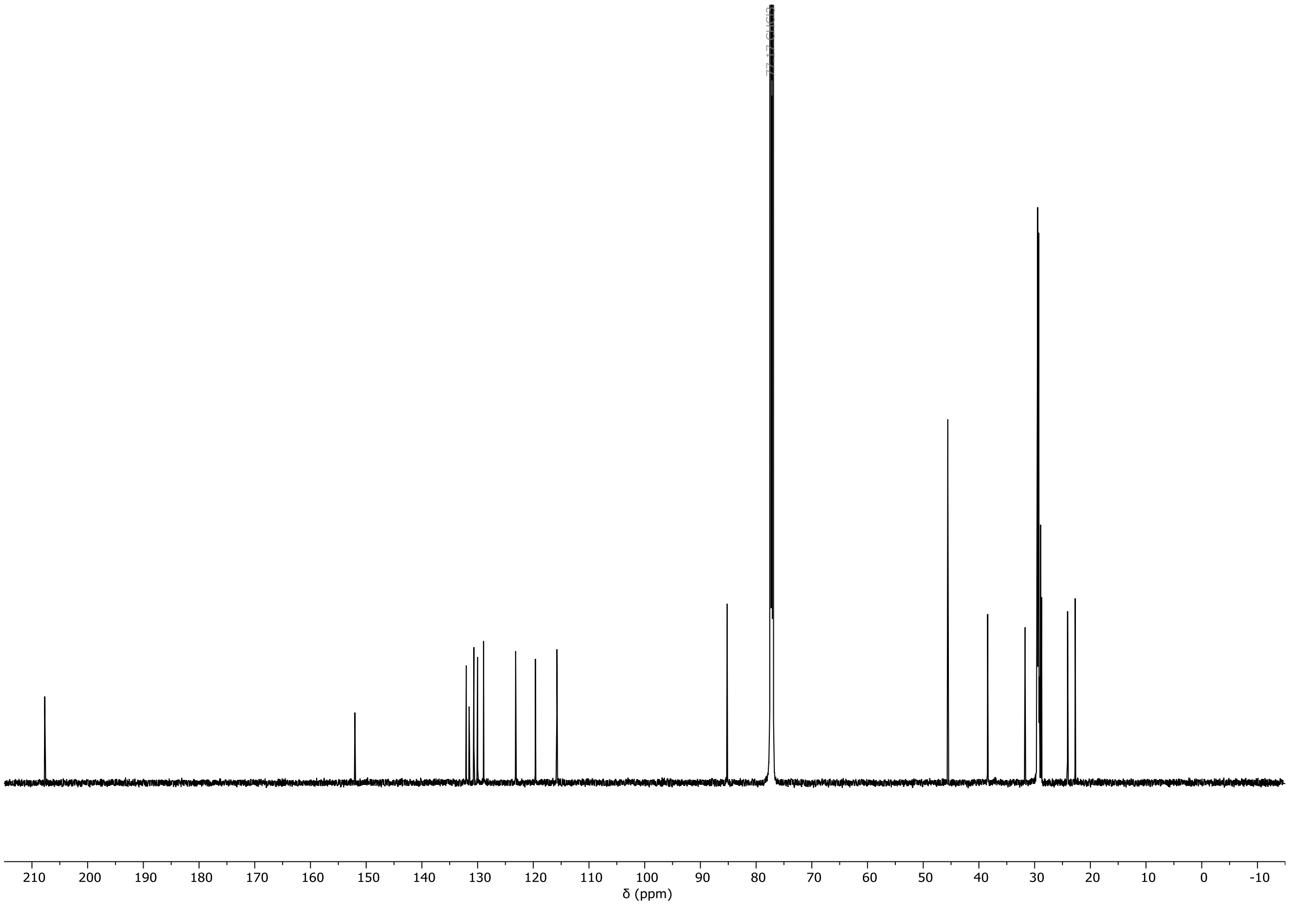

Spectrum S27: ^{13}C NMR (151 MHz, 298 K, $CDCl_3$) of **S4**.

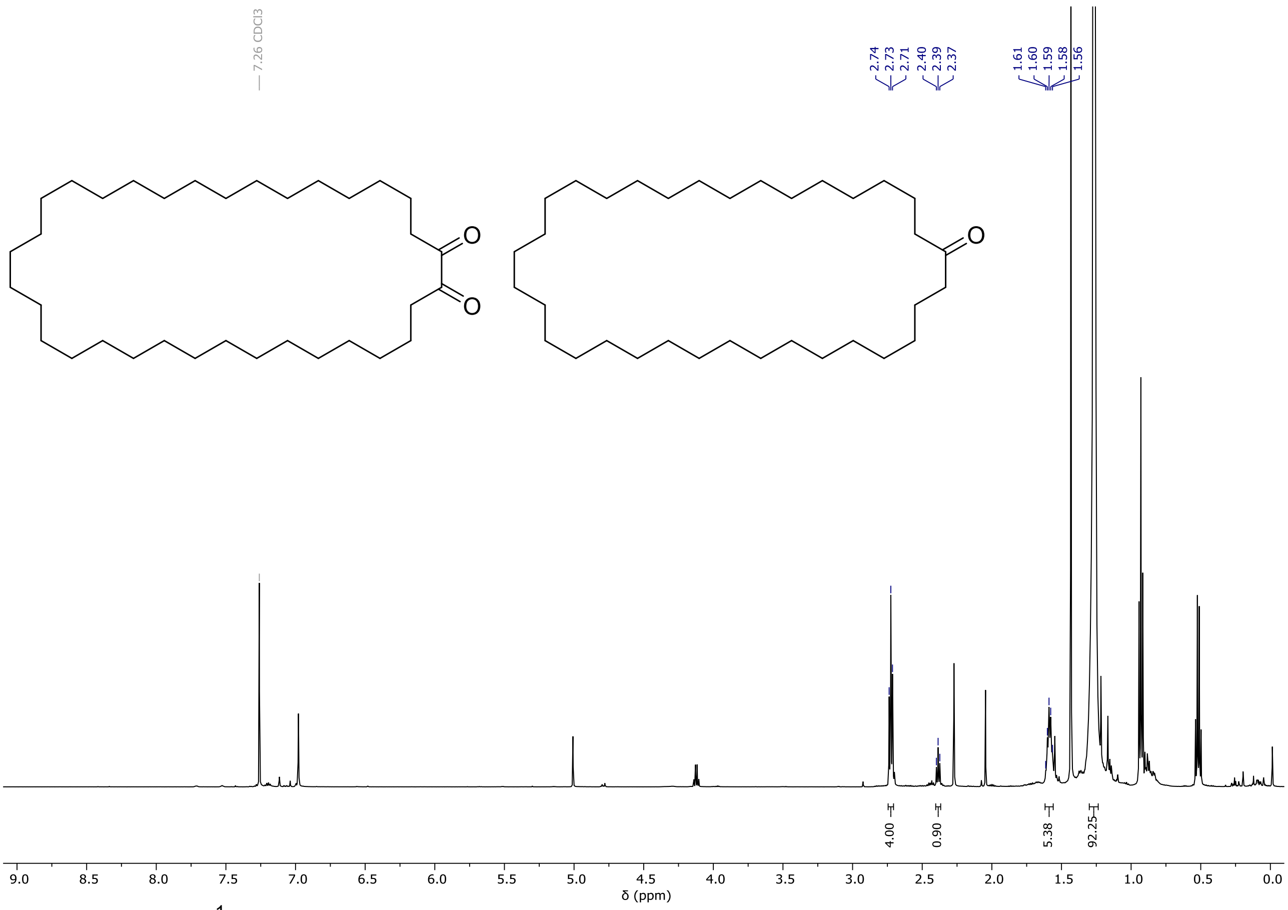

Spectrum S28: ^{1}H NMR (600 MHz, 298 K, $CDCl_3$) of **S5** and **S6**.

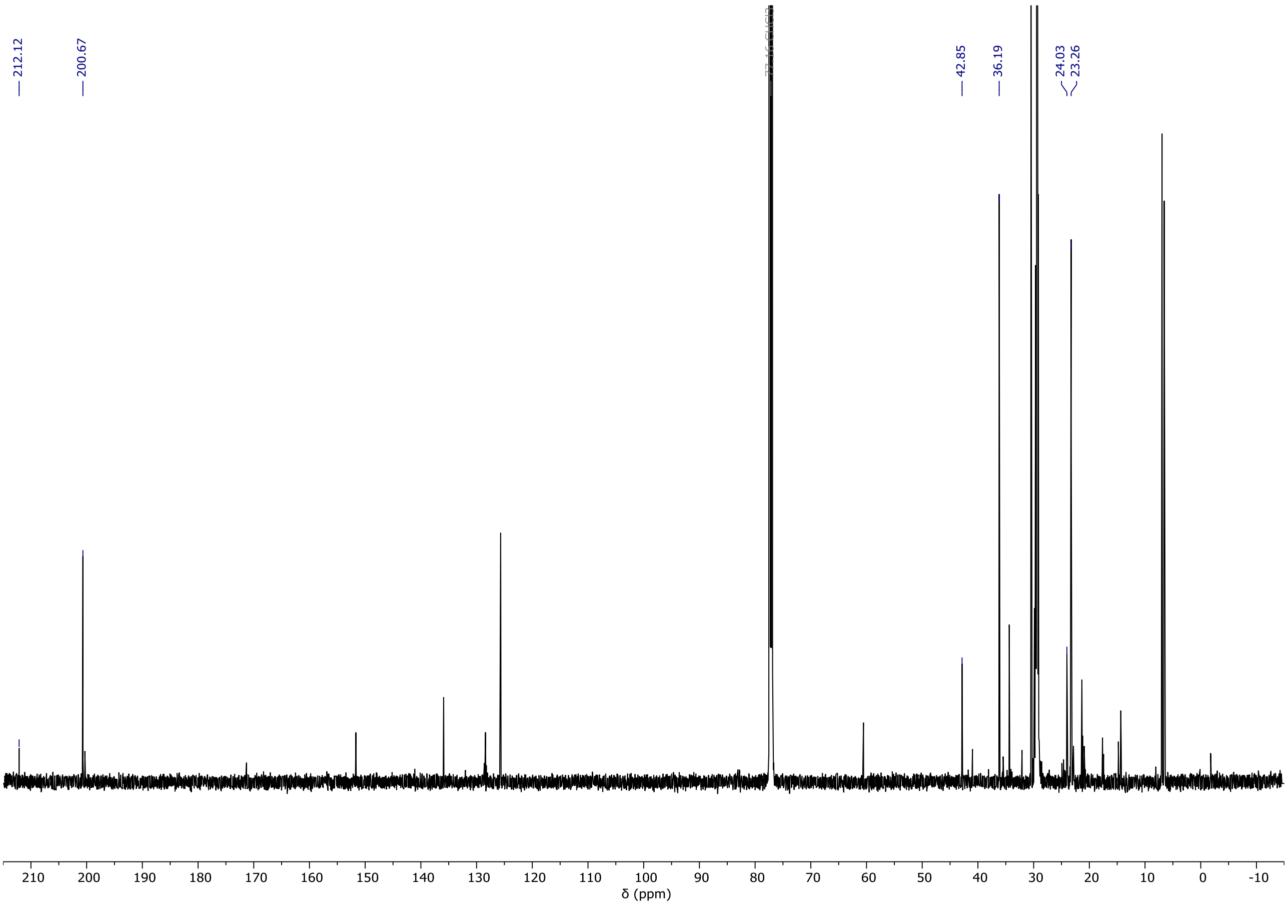

Spectrum S29: ^{13}C NMR (151 MHz, 298 K, $CDCl_3$) of **S5** and **S6**.

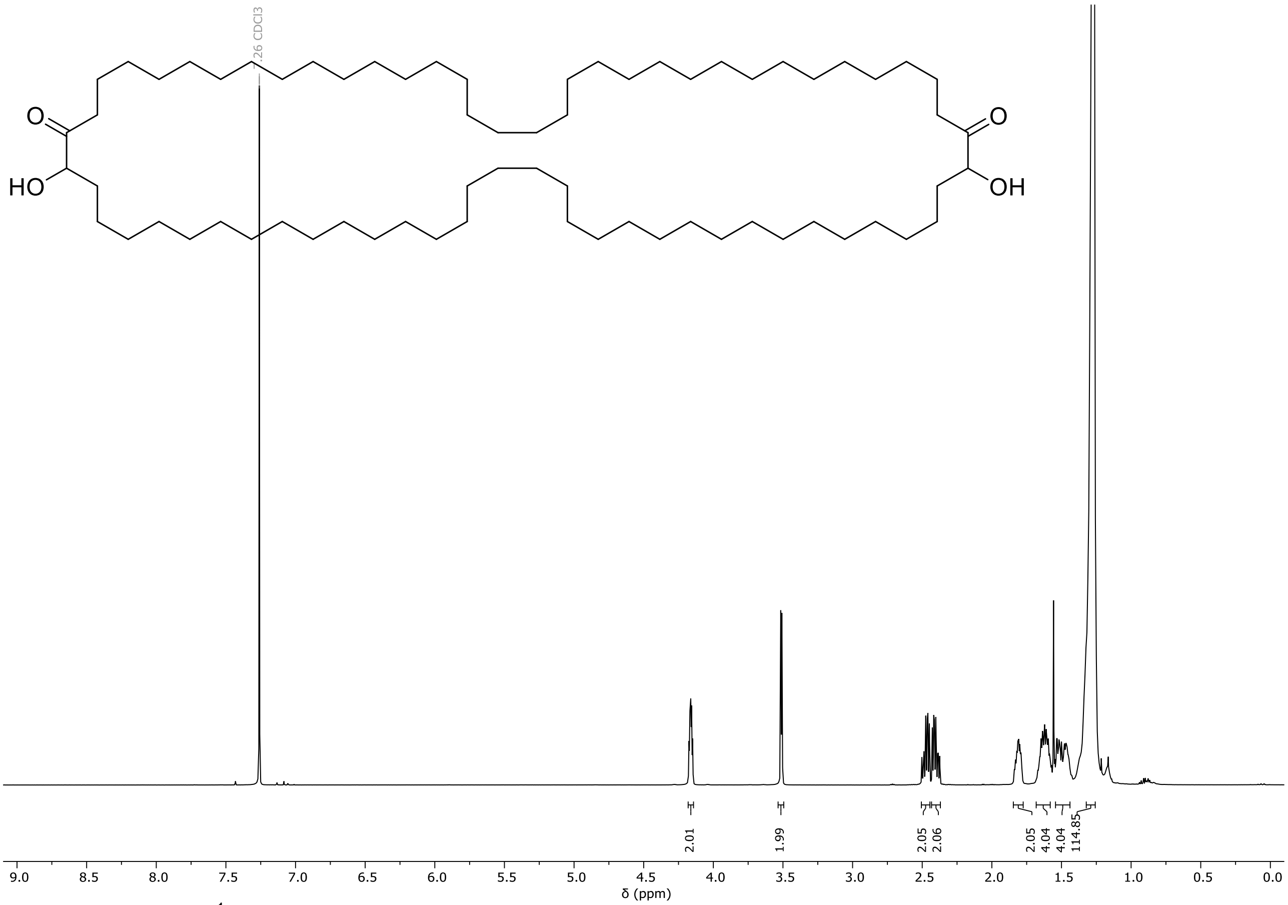

Spectrum S30: ^{1}H NMR (600 MHz, 298 K, $CDCl_3$) of **S7**.

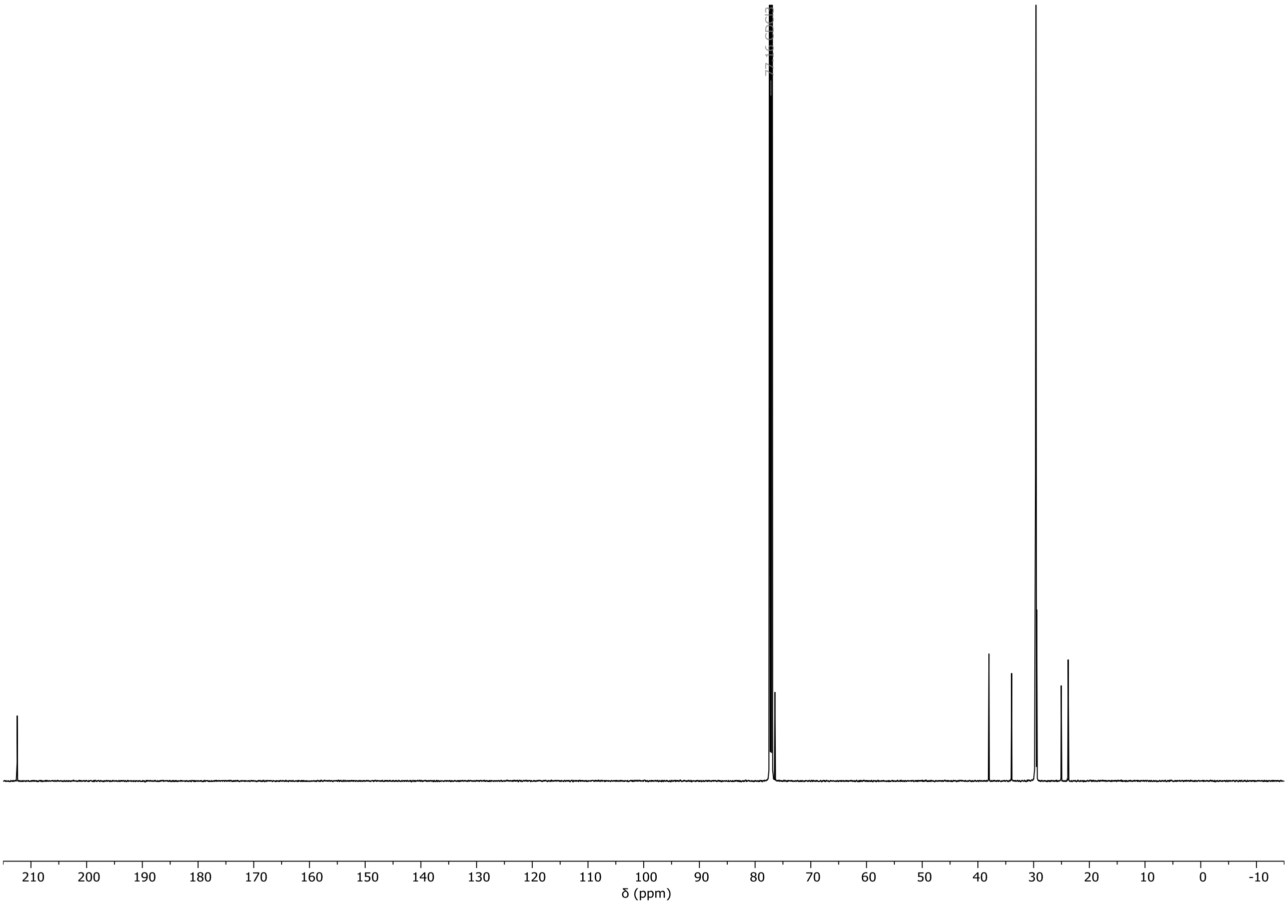

Spectrum S31: ^{13}C NMR (151 MHz, 298 K, $CDCl_3$) of **S7**.

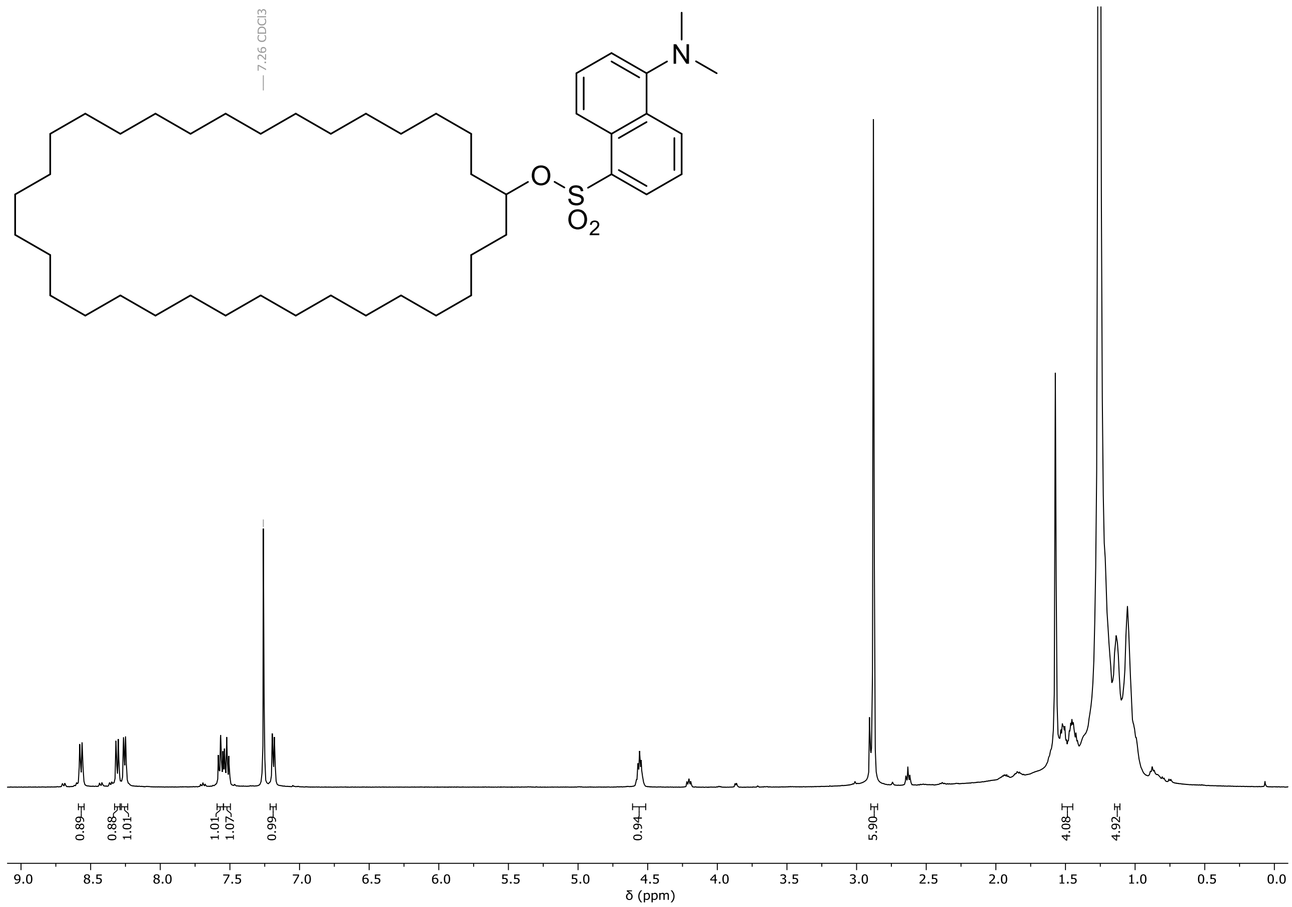

Spectrum S32: ^{1}H NMR (600 MHz, 298 K, $CDCl_3$) of **S10b**.

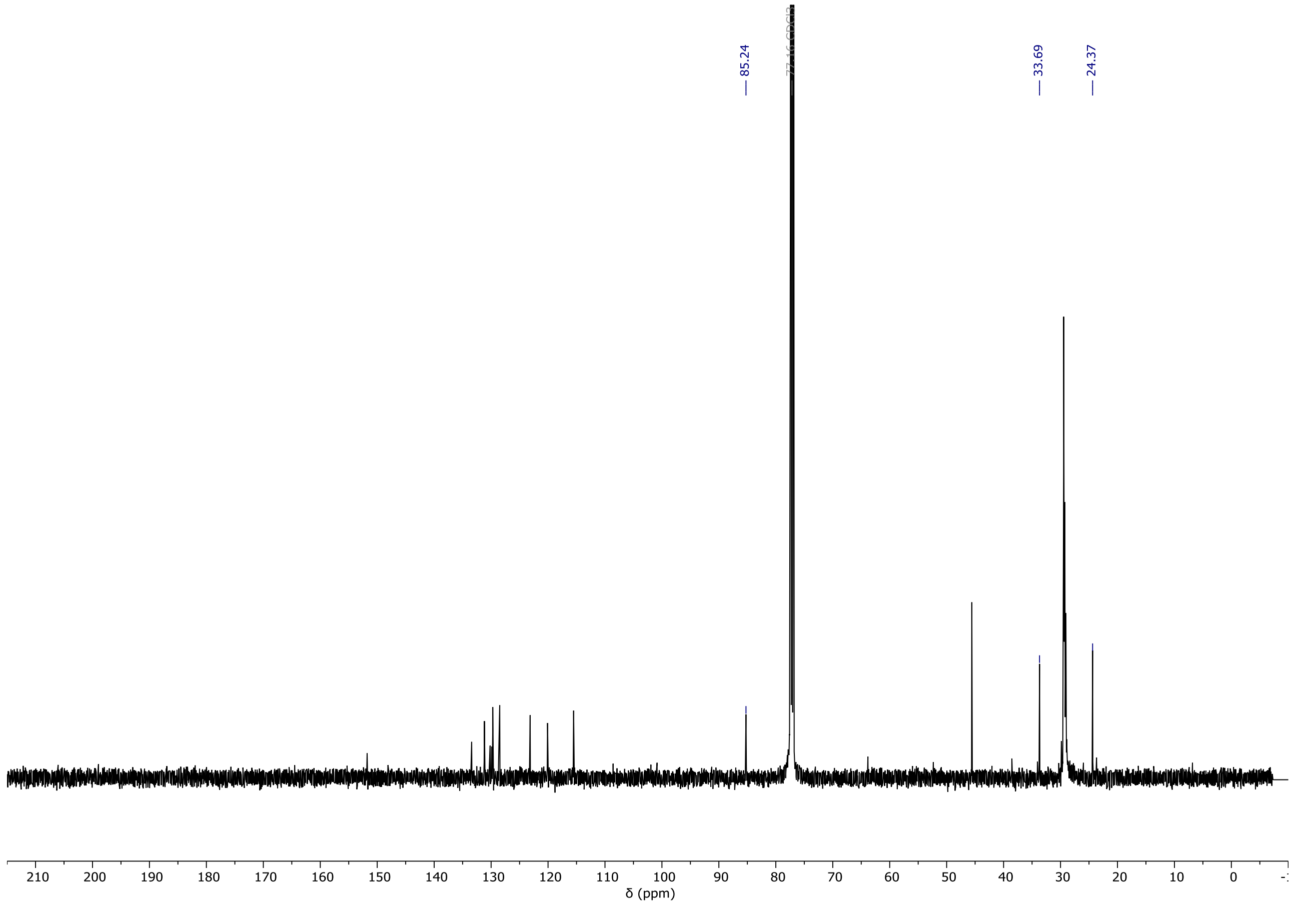

Spectrum S33: ^{13}C NMR (151 MHz, 298 K, $CDCl_3$) of **S10b**.

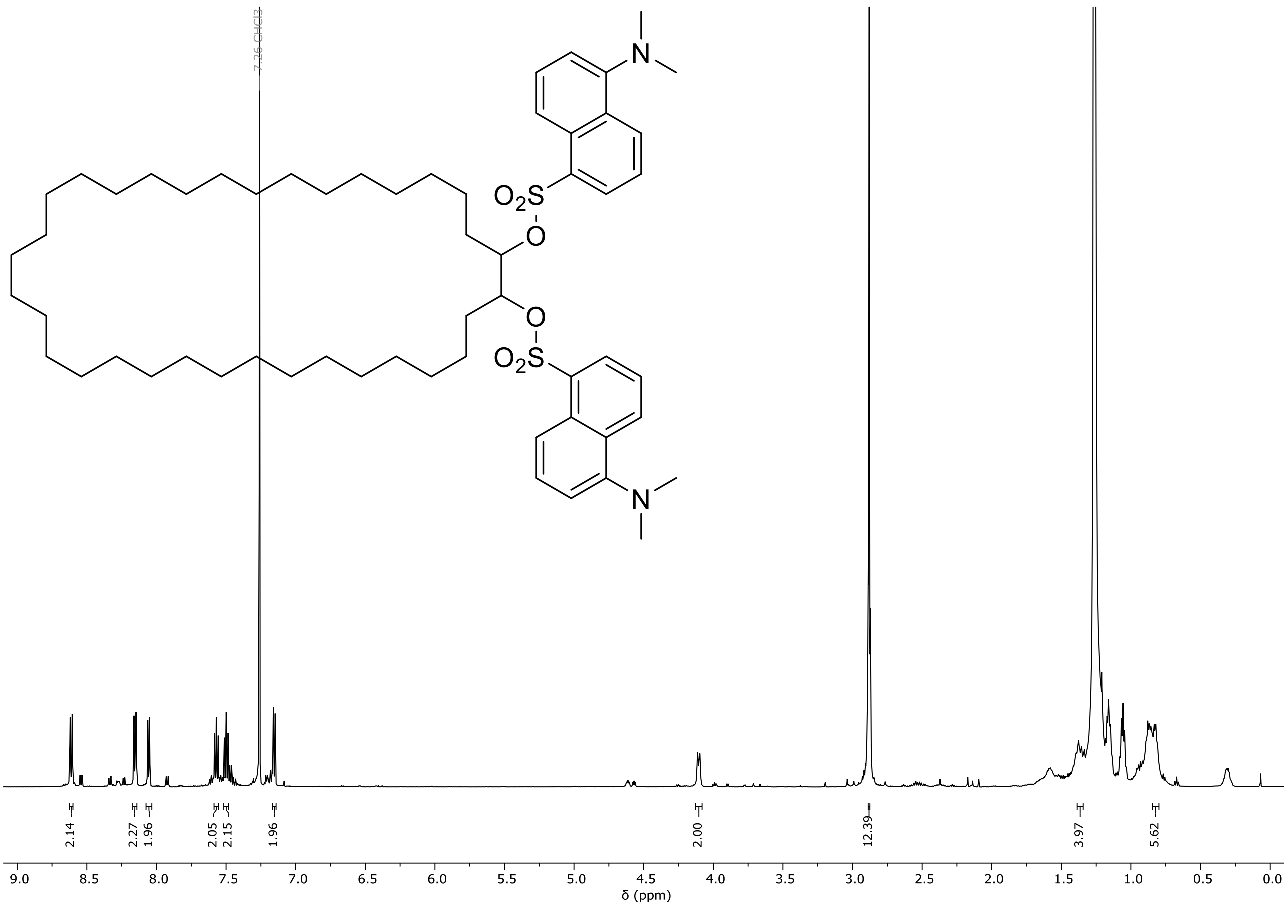

Spectrum S34: ^{1}H NMR (600 MHz, 298 K, $CDCl_3$) of **S13**.

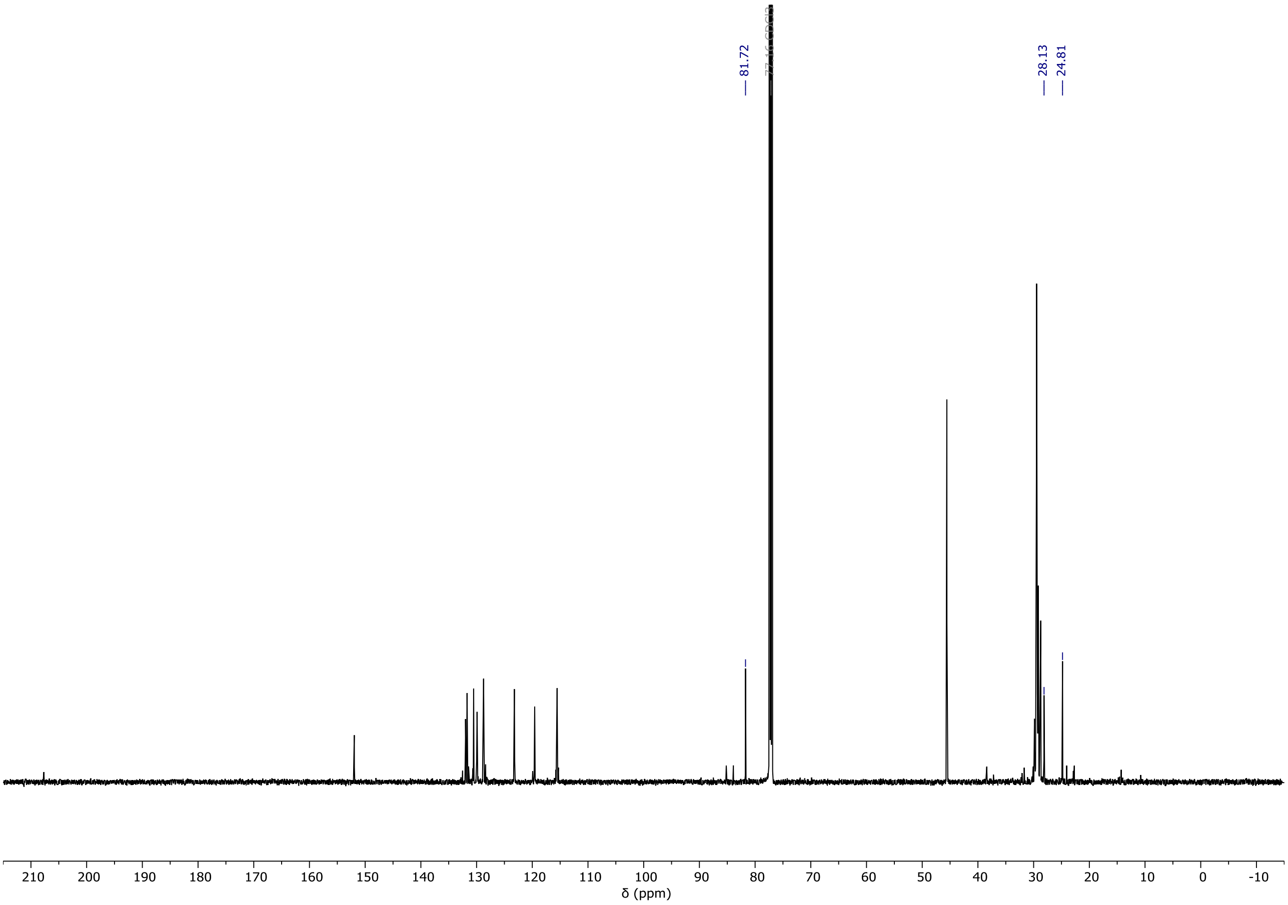

Spectrum S35: ^{13}C NMR (151 MHz, 298 K, $CDCl_3$) of **S13**.

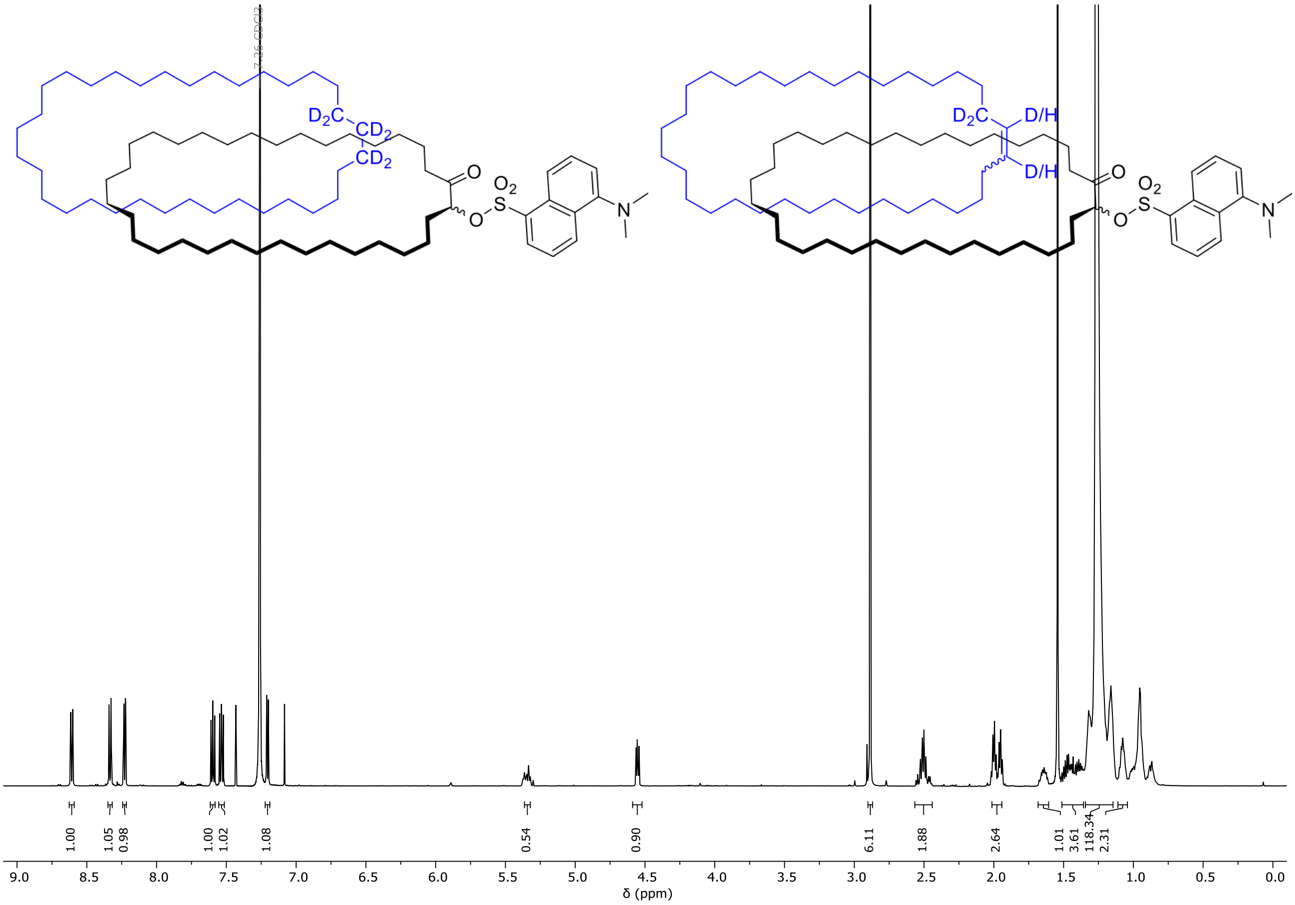

Spectrum S36: ¹H NMR (600 MHz, 298 K, $CDCl_3$) of **12a/12b**.

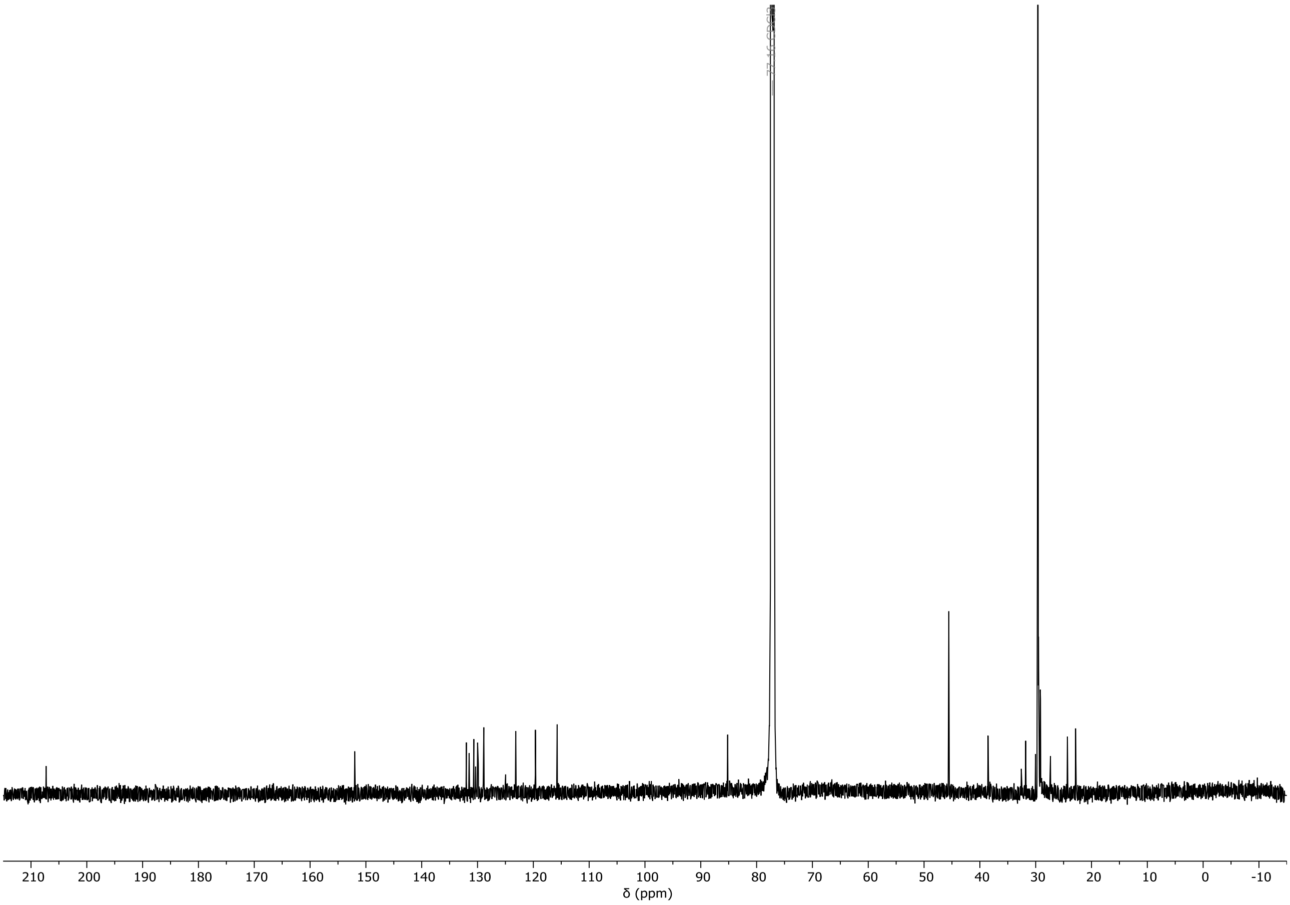

Spectrum S37: ¹H NMR (151 MHz, 298 K, $CDCl_3$) of **12a/12b**.

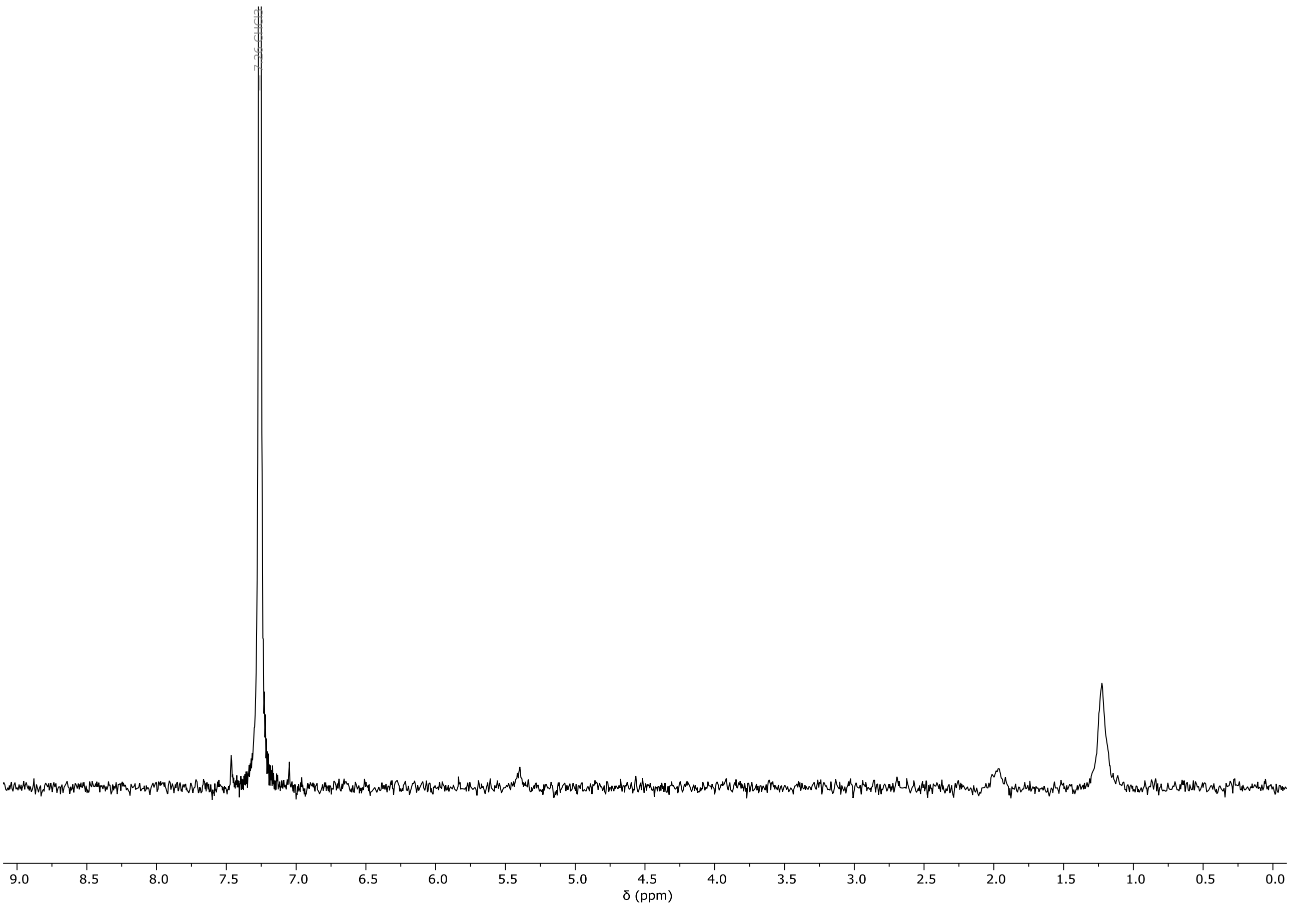

Spectrum S38: ^{2}H NMR (77 MHz, 298 K, $CHCl_3$) of **12a/12b**

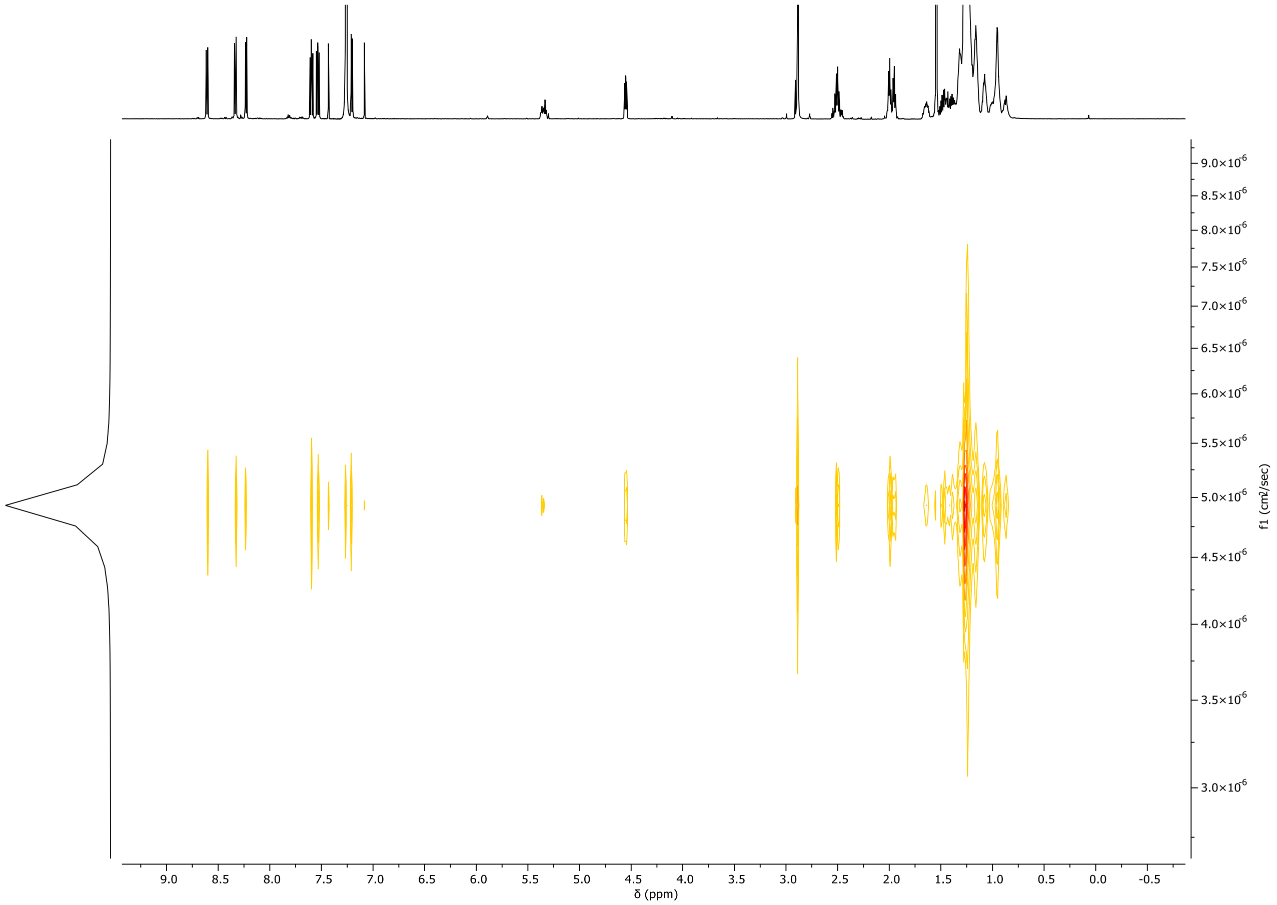

Spectrum S39: DOSY NMR (600 MHz, 298 K, $CDCl_3$) of **12a/12b**.

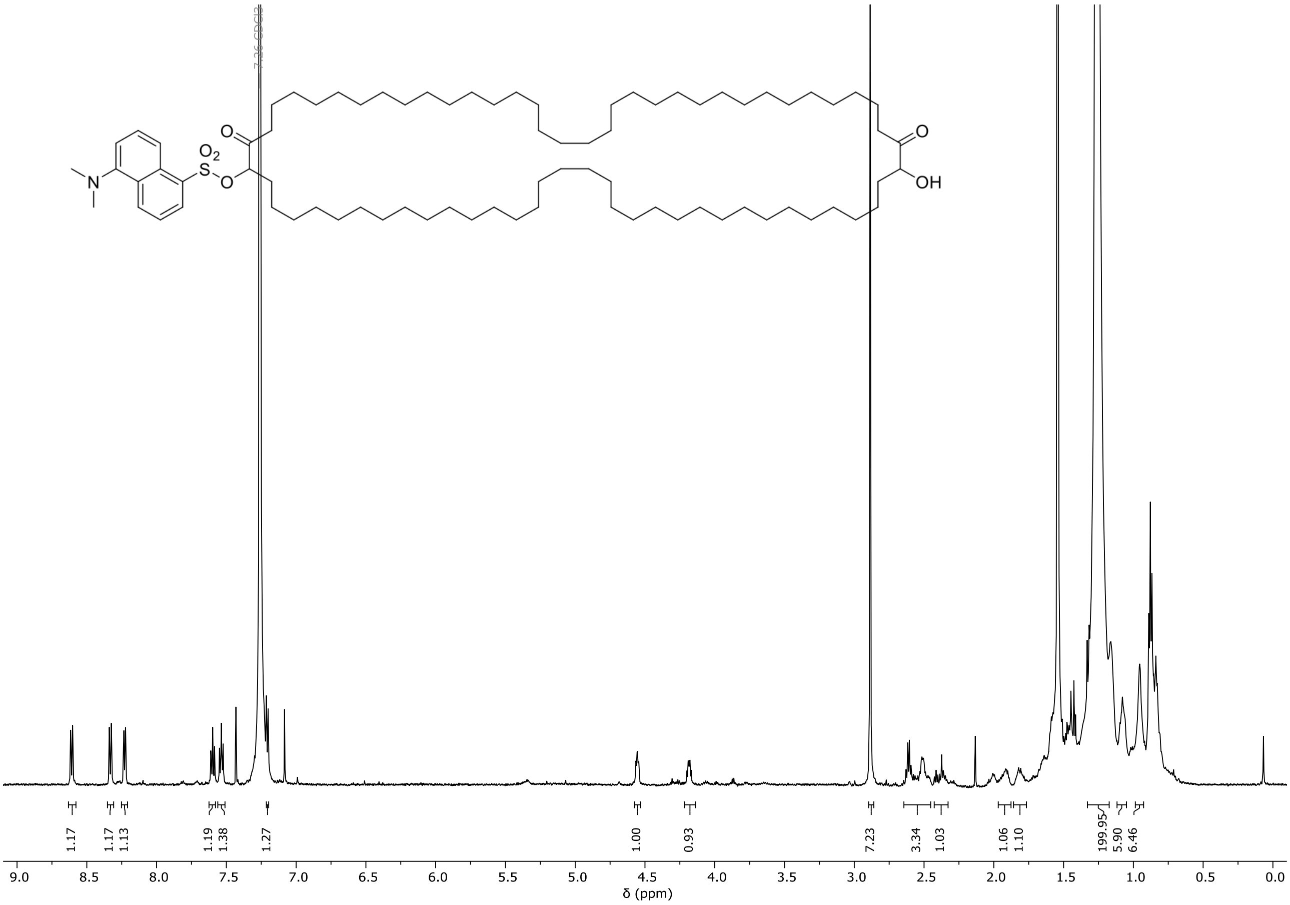

Spectrum S40: ^{1}H NMR (600 MHz, 298 K, $CDCl_3$) of **S15**.

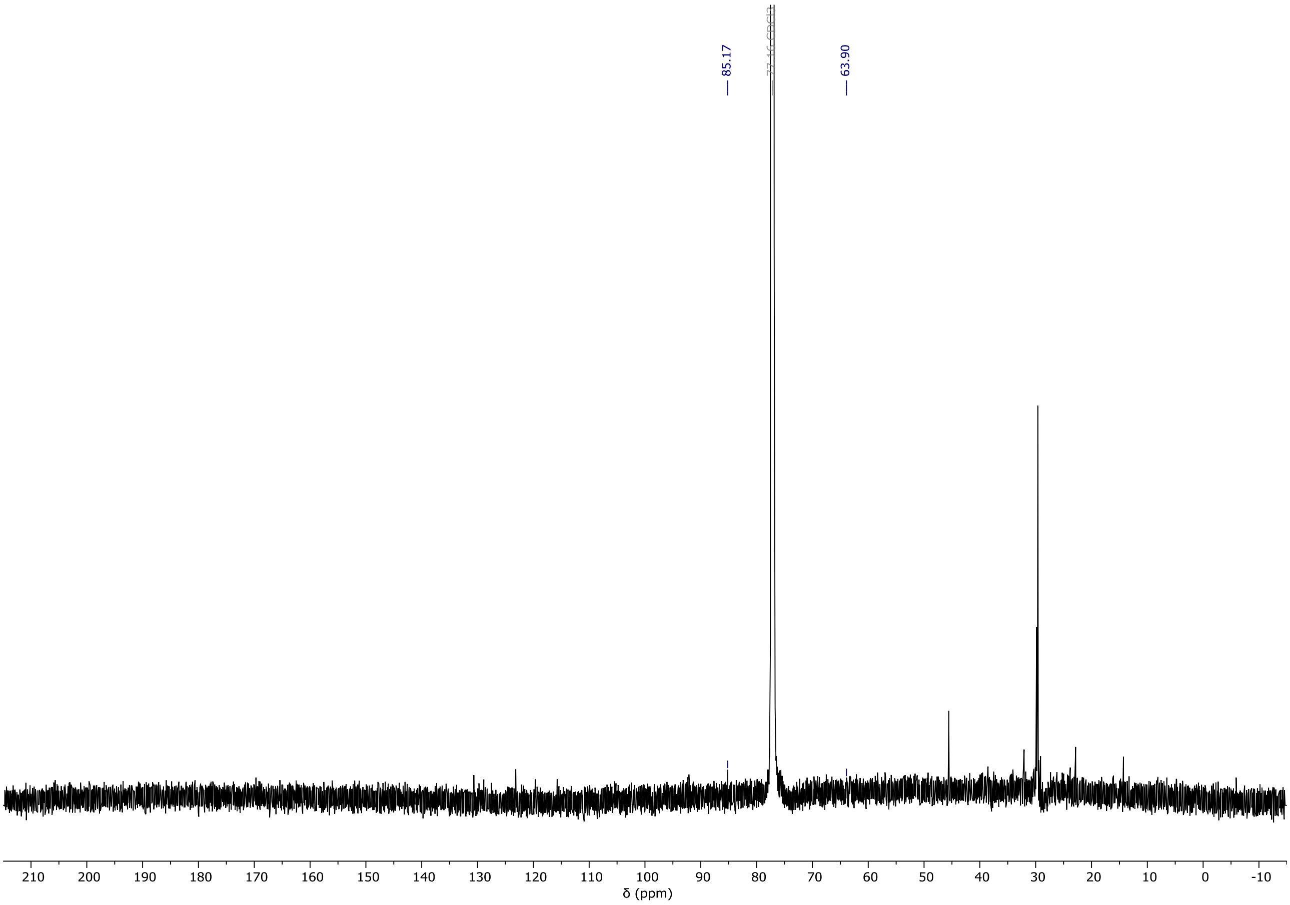

Spectrum S41: ^{13}C NMR (151 MHz, 298 K, $CDCl_3$) of **S15**.

10. X-ray data

Data collection: X-ray diffraction data were collected for compounds **3** and **4** on a dual source Rigaku FR-X rotating anode at 100 K with Cu-Kα (1.54184 Å) radiation, equipped with a Hypix000HE detector and Oxford cryosystem. All data were collected using CrysAlisPro software.

Crystal structure determination and refinements: X-ray data were processed and reduced using CrysAlisPro. Absorption correction was performed using empirical methods (SCALE3 ABSPACK) based upon symmetry-equivalent reflections combined with measurements at different azimuthal angles. The crystal structure was solved and refined against all F^2 values using the SHELX and Olex2 suite of programmes[7.8] All atoms were refined anisotropically. Hydrogen atoms were placed in calculated positions and refined using idealized geometries and assigned fixed isotropic displacement parameters. The location of deuterium atoms in compound **3** couldn't be determined crystallographically. Therefore, the deuterium atoms were placed in atoms C1-3 with 50 % occupancy, in order to correct the formula of compound **3**. Data for crystal structure **4** was found to be twinned.

Crystallographic data have been deposited with the CCDC (2218853-2218854).

Table S1. Crystallographic parameters for crystal structures of compounds **3** and **4**

Identification code	**3**	**4**
Empirical formula	$C_{34}H_{62}D_6$	$C_{34}H_{66}O_2$
Formula weight	482.92	506.86
Temperature/K	240.15	100.00(10)
Crystal system	triclinic	triclinic
Space group	P-1	P-1
a/Å	5.4067(3)	5.28040(10)
b/Å	8.0631(6)	8.2105(3)
c/Å	18.9608(11)	38.033(2)
α/°	83.618(5)	84.969(4)
β/°	87.116(5)	86.267(3)
γ/°	76.598(6)	75.648(3)
Volume/Å^3	798.84(9)	1589.68(11)
Z	1	2
ρ_{calc}g/cm^3	1.004	1.059
μ/mm^{-1}	0.388	0.466
F(000)	272.0	572.0
Crystal size/mm^3	0.6 × 0.5 × 0.2	0.25 × 0.09 × 0.08
Radiation	Cu Kα (λ = 1.54184)	Cu Kα (λ = 1.54184)
2Θ range for data collection/°	4.692 to 151.832	7.006 to 155.386

Index ranges	$-4 \le h \le 6$, $-10 \le k \le 9$, $-23 \le l \le 23$	$-6 \le h \le 6$, $-10 \le k \le 10$, $-48 \le l \le 47$
Reflections collected	6833	9560
Independent reflections	3172 [R_{int} = 0.0217, R_{sigma} = 0.0332]	9560 [R_{int} = 0.088, R_{sigma} = 0.0386]
Data/restraints/parameters	3172/0/154	9560/0/327
Goodness-of-fit on F^2	1.085	1.075
Final R indexes [I>=2σ (I)]	R_1 = 0.0485, wR_2 = 0.1447	R_1 = 0.0887, wR_2 = 0.2606
Final R indexes [all data]	R_1 = 0.0587, wR_2 = 0.1515	R_1 = 0.1160, wR_2 = 0.2928
Largest diff. peak/hole / e $Å^{-3}$	0.18/-0.14	0.34/-0.36

11. References

1. Song, S.; Rudick, J. G. Efficient Syntheses of Star-Branched, Multifunctional Mesogens. *Org. Lett.*, **2015**, *17*, 3244–3247.
2. Weichert, L.; Schwendner, C. Potential tumor- or organ-imaging agents. 26. Polyiodinated 2-substituted triacylglycerols as hepatographic agents. *J. Med. Chem.*, **1986**, *29*, 1674–1682.
3. Koyanagi, T.; Leriche, G.; Onofrei, D.; Holland, G. P.; Mayer, M.; Yang, J. Cyclohexane Rings Reduce Membrane Permeability to Small Ions in Archaea-Inspired Tetraether Lipids. *Angew. Chem. Int. Ed.*, **2016**, *55*, 1890–1893.
4. Schaubach, S.; Gebauer, K.; Ungeheuer, F.; Hoffmeister, L.; Ilg, M. K.; Wirtz, C.; Fürstner, A. A Two-Component Alkyne Metathesis Catalyst System with an Improved Substrate Scope and Functional Group Tolerance: Development and Applications to Natural Product Synthesis. *Chem. Eur. J.*, **2016**, *22,* 8494–8507.
5. Bell, S. E.; Ewing, R. G.; Eiceman, G. A. Atmospheric pressure chemical ionization of alkanes, alkenes, and cycloalkanes. *J. Am. Soc. Mass Spectrom.*, **1994**, *5*,177–185.
6. Manheim, J. M.; Milton, J. R.; Zhang, Y.; Kenttämaa, H. I. Fragmentation of Saturated Hydrocarbons upon Atmospheric Pressure Chemical Ionization Is Caused by Proton-Transfer Reactions. *Anal. Chem.*, **2020**, *92*, 8883–8892.
7. Sheldrick, G. M. Crystal structure refinement with SHELXL. *Acta Crystallogr. Sect. C Struct. Chem.* **2015**, *71*, 3–8;
8. Dolomanov, O. V.; Bourhis, L. J.; Gildea, G. J.; Howard, J. A. K.; Puschmann, H. OLEX2: a complete structure solution, refinement and analysis program. *J. Appl. Crystallogr.*, **2009**, *42*, 339–341.
